# Hsp70 Forms Antiparallel Dimers Stabilized by Post-translational Modifications to Position Clients for Transfer to Hsp90

**DOI:** 10.1016/j.celrep.2015.03.063

**Published:** 2015-04-23

**Authors:** Nina Morgner, Carla Schmidt, Victoria Beilsten-Edmands, Ima-obong Ebong, Nisha A. Patel, Eugenia M. Clerico, Elaine Kirschke, Soumya Daturpalli, Sophie E. Jackson, David Agard, Carol V. Robinson

**Affiliations:** 1 Department of Chemistry, University of Oxford, South Parks Road, Oxford OX1 3QZ, UK; 2 Department of Biochemistry and Molecular Biology, University of Massachusetts, Amherst, MA 01003, USA; 3 Department of Biochemistry and Biophysics, Howard Hughes Medical Institute, University of California, San Francisco, San Francisco, CA 94158, USA; 4 Department of Chemistry, University of Cambridge, Lensfield Road, Cambridge CB2 1EW, UK

## Abstract

Protein folding in cells is regulated by networks of chaperones, including the heat shock protein 70 (Hsp70) system, which consists of the Hsp40 cochaperone and a nucleotide exchange factor. Hsp40 mediates complex formation between Hsp70 and client proteins prior to interaction with Hsp90. We used mass spectrometry (MS) to monitor assemblies formed between eukaryotic Hsp90/Hsp70/Hsp40, Hop, p23, and a client protein, a fragment of the glucocorticoid receptor (GR). We found that Hsp40 promotes interactions between the client and Hsp70, and facilitates dimerization of monomeric Hsp70. This dimerization is antiparallel, stabilized by post-translational modifications (PTMs), and maintained in the stable heterohexameric client-loading complex Hsp90_2_Hsp70_2_HopGR identified here. Addition of p23 to this client-loading complex induces transfer of GR onto Hsp90 and leads to expulsion of Hop and Hsp70. Based on these results, we propose that Hsp70 antiparallel dimerization, stabilized by PTMs, positions the client for transfer from Hsp70 to Hsp90.

## Highlights

• Antiparallel dimerization of Hsp70 is stabilized by PTMs
• Hsp40 catalyzes Hsp70 dimerization and client transfer to Hsp70
• Hsp70 antiparallel dimerization is maintained in the client-loading complex
• Addition of p23 induces transfer of GR onto Hsp90 and loss of Hop and Hsp70

## Graphical Abstract

**Figure:**
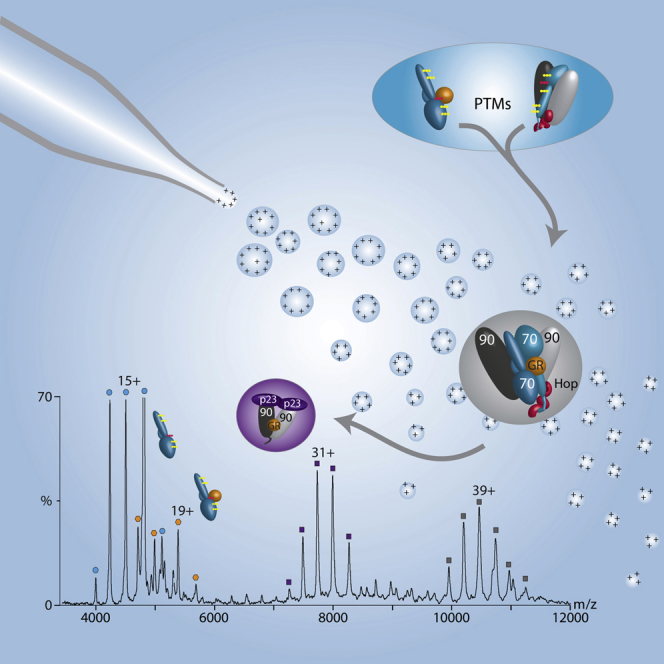

## Teaser

Morgner et al. combine native mass spectrometry and chemical crosslinking to define the interactions of the Hsp70/90 chaperone system. They show that Hsp70 dimerization is antiparallel and stabilized by PTMs. They monitor the formation of chaperone complexes and discover a hexameric client-loading complex containing an Hsp70 dimer.

## Introduction

Hsp70 and Hsp90 are essential and abundant molecular chaperones in the eukaryotic cytosol and are involved in the folding and maturation of a myriad of protein substrates, including many cancer-causing proteins (Brychzy et al., 2003, Taipale et al., 2010). The Hsp70/90 system requires a cohort of cochaperones to provide specificity and regulation of the chaperone interactions with their client proteins (Li et al., 2011, Picard et al., 1990, Young et al., 2001). Hsp70 binds extended hydrophobic peptide sequences and acts at an early stage to recognize partially folded client proteins. Hsp90 is thought to interact with near-native conformations of its substrates, and its clients include protein kinases and steroid hormone receptors, the latter being the most extensively studied (Jackson, 2013).

Hsp90 forms defined binary or ternary complexes with cochaperones to facilitate the maturation of client proteins (reviewed in Prodromou, 2012). Structurally, it consists of a C-terminal dimerization region, a middle domain, and an N-terminal nucleotide-binding domain (NBD) connected by a charged linker that provides the necessary flexibility for domain rearrangements (Tsutsumi et al., 2012). Hsp90 binds at its C-terminal MEEVD sequence to tetratricopeptide repeat (TPR) cochaperones, including the “Hsc70/Hsp90 organizing protein” Hop (Young et al., 1998).

In contrast to Hsp90, Hsp70 is predominantly monomeric, and high-resolution structures of the full-length eukaryotic protein have remained elusive. By analogy to the different ADP-bound states of DnaK, the *E. coli* Hsp70 homolog, the picture that is emerging shows independent movements of the N-terminal NBD and C-terminal substrate-binding domain (SBD) (Bertelsen et al., 2009, Swain et al., 2007). These movements are thought to be lost upon ATP binding when Hsp70 rearranges into a domain-docked structure (Mapa et al., 2010). Together with a previous NMR study (Zhuravleva et al., 2012), these structures define the allosteric control mechanism between the NBD and SBD.

In previous studies, we established that Hsp70 was essentially monomeric under our solution conditions (Ebong et al., 2011), although dimerization has been reported previously in solution and X-ray structures of DnaK (Qi et al., 2013). Recently, specific mutations of DnaK were designed to disrupt the dimer interface observed crystallographically and to probe its functional significance (Sarbeng et al., 2015). Results showed that mutations on the dimer interface compromise both chaperone activity and Hsp40 interactions.

It is established that the Hsp40 cochaperones regulate ATP-dependent substrate binding of Hsp70 (Laufen et al., 1999, Mayer and Bukau, 2005) via interaction of the N-terminal J domains of Hsp40 with an acidic groove located in the NBD of Hsp70 (Jiang et al., 2007). The C-terminal domain of Hsp40 from yeast contains an EEVD peptide-binding site (Li et al., 2006) and the dimerization domain (Li et al., 2003). Hsp40s are typically expressed at lower levels than Hsp70, consistent with their catalytic function and crucial role in Hsp70 function (Young, 2010). In addition, numerous reports have proposed an Hsp40-induced oligomerization of human Hsp70 or DnaK (Benaroudj et al., 1995, Cyr and Douglas, 1994, Hernández et al., 2002, Thompson et al., 2012). Participation of the Hsp70/Hsp40 chaperone system is also required to regulate client binding to Hsp90 (Pratt and Toft, 1997) and to load Hsp90 with a client protein (Hernández et al., 2002). Hop bridges the Hsp70 and Hsp90 chaperone systems (Chen and Smith, 1998), and also inhibits the ATPase activity of Hsp90, stabilizing the client-loading conformation and facilitating the handover of clients (Richter et al., 2003, Southworth and Agard, 2011).

For this study, we selected the transcription factor GR as the client protein because it is well characterized, particularly with respect to its associations with the Hsp90/Hsp70 systems (Sanchez, 2012). GR has to bind to Hsp90 as a prerequisite to attain a high-affinity ligand-binding conformation prior to its import into the nucleus (Dittmar et al., 1997, Picard et al., 1990, Smith, 1993). Here, we used a structure of its ligand-binding domain (LBD) in complex with the agonist dexamethasone (Bledsoe et al., 2002). During the course of this work, two cryo-electron microscopy (cryo-EM) studies (Alvira et al., 2014, Kirschke et al., 2014) revealed the formation of an Hsp90/Hsp70/Hop/GR complex proposing unfolding/inactivation of GR by Hsp70 and refolding/reactivation of GR by Hsp90, and revealing the location of GR with respect to Hop, which is located on the opposite side of the Hsp90. These complexes provide new insight into the location of GR, but also raise the question of whether they are primed for transfer of GR from Hsp70 to Hsp90.

In common with many chaperone systems, Hsp70/90-client interactions have proven challenging to study with traditional biophysical techniques due to their dynamic nature and compositional heterogeneity. For this reason, we applied mass spectrometry (MS) to probe the composition of stable complexes formed on these reaction pathways. The use of MS to study protein complexes is gaining momentum (Heck, 2008, Hilton and Benesch, 2012, Wyttenbach and Bowers, 2007). Pertinent to such studies is the ability of MS to determine the masses and hence the subunit stoichiometry of multi-protein complexes that form simultaneously within dynamic and heterogeneous assemblies (Benesch et al., 2006, Stengel et al., 2010). When coupled with chemical crosslinking (XL) strategies (Schmidt and Robinson, 2014), this approach is particularly powerful because it allows not only the stoichiometry but also the interaction interfaces to be defined (Schmidt et al., 2013).

By incubating subsets of proteins involved in the assembly of Hsp90/Hsp70/Hop/GR complexes, and by varying the order of addition and concentrations of the proteins and the level of nucleotides, we allow the Hsp90/Hsp70/Hsp40/GR complexes to assemble in solution. We then define their composition and interactions by recording mass spectra of the intact complexes. In addition, by employing chemical XL, we identify the interfaces between the complex components. Starting with Hsp70, we explore the extent of its oligomerization in solution with respect to its post-translational modification (PTM) status, and employ comparative chemical XL to compare dimeric interfaces in ATP- and ADP-bound states. Increasing the complexity to the Hsp70/Hsp40/GR system, we show that we can detect stable Hsp70-client complexes in the presence of catalytic quantities of Hsp40. We find that the predominant client-loading complex formed with Hsp90 and Hop contains dimeric Hsp70 and Hsp90, as well as one Hop and one GR. Chemical XL locates GR in close proximity to Hsp90 and enables us to identify roles for Hsp40 and Hsp70 in transferring clients to the Hsp90 cycle, and to propose a role for Hsp70 dimerization in this client-loading complex.

## Results

### PTMs Stabilize Hsp70 Dimers

**Figure 1:**
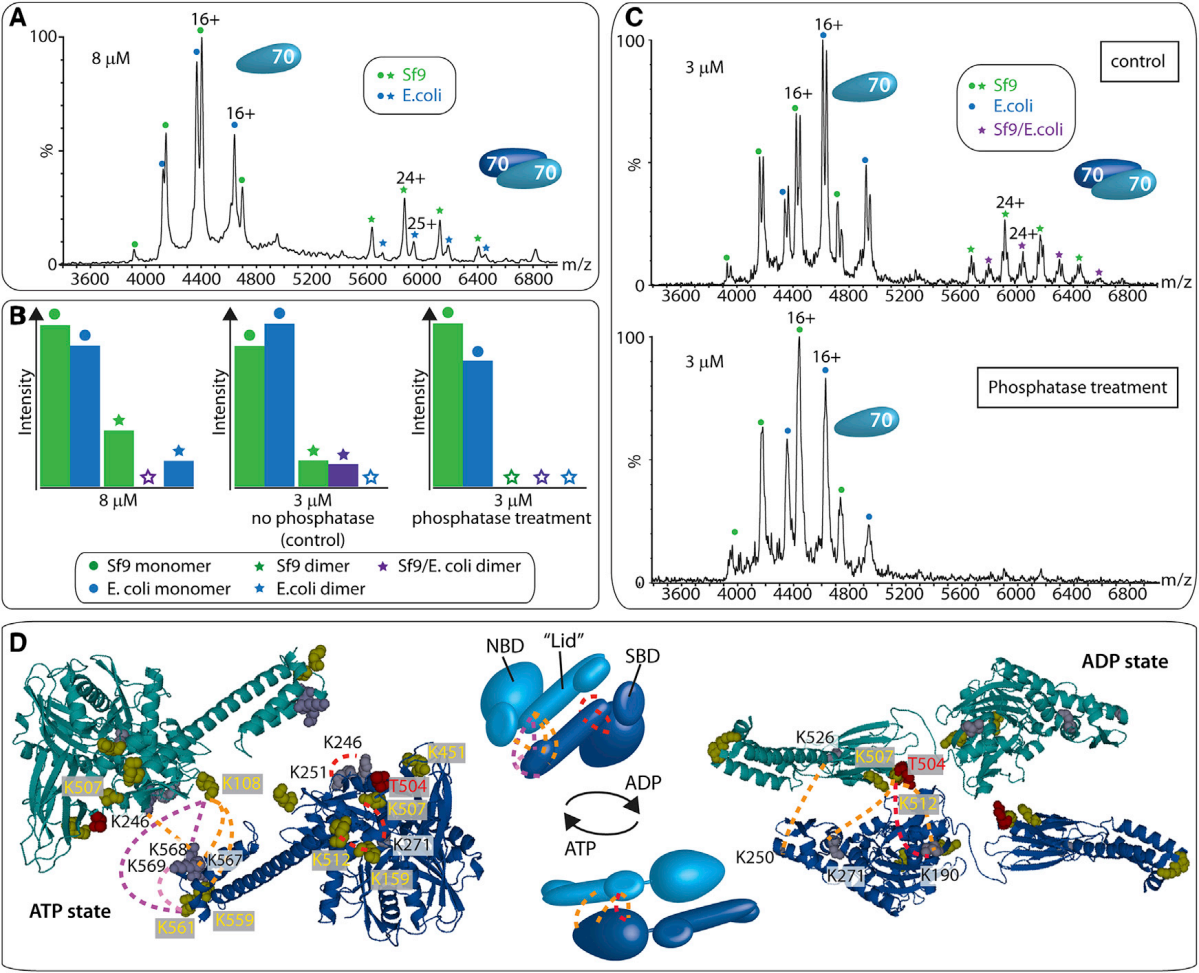
PTMs Promote Dimerization of Hsp70 (A) Mass spectrum of an 8 μM solution containing a 1:1 ratio of ^13^C-labeled Hsp70_*E. coli*_ and Hsp70_Sf9_ with natural abundance isotopes. At these concentrations, both are predominantly monomeric, but a higher population of Hsp70 dimers is observed for the Sf9-expressed protein. (B) Bar charts comparing the intensities of monomeric and dimeric Hsp70. Green, Sf9; blue, *E. coli*; purple, Sf9/*E. coli*. Monomers and dimers are labeled with circles and stars, respectively. (C) Mass spectrum recorded at high backing pressure to promote dimer formation (Hernández and Robinson, 2007) of a 1:1 ratio of 3 μM ^13^C- labeled Hsp70_*E. coli*_ and Hsp70_Sf9_ with natural abundance isotopes, incubated overnight in the absence of phosphatase (“control,” top) or in the presence of phosphatase (“phosphatase treatment,” bottom). Both spectra were acquired under the same conditions. Note the absence of dimer under the phosphatase condition. (D) XLs are highlighted on structures of Hsp70 subunits in ATP and ADP states. The ADP state was manually aligned with the human Hsp70 sequence. Inter-subunit XLs are highlighted on antiparallel dimers of both states (shown schematically). In the ATP state, XLs K108-K561/569 (purple) orient two Hsp70 subunits in an antiparallel dimer. The phosphosite T504 in Hsp70_Sf9_ (red) and the lysine-binding pockets are located both adjacent to and across the interface (gray and yellow). Comparative XL of the two Hsp70 proteins (expressed in *E. coli* and Sf9) shows that the K108-K569/561 is 5-fold more intense in Hsp70_Sf9_ than in Hsp70_*E. coli*_, consistent with the stronger interface in Hsp70_Sf9_ (purple). Less dimerization for the *E. coli-*expressed Hsp70 is consistent with the observed 3-fold increase of the K569-561 intra-XL. Inter-subunit XLs (red) are consistent with the ATP state. See also Figures S1–S4 and Tables S1, S2, and S3.

To investigate interactions in Hsp70 and to probe the existence of higher oligomeric states proposed previously (Aprile et al., 2013, King et al., 1999), we examined by MS the propensity of Hsp70 to form oligomers. We expressed and purified human Hsp70 in two expression systems: Sf9 insect cells and *E. coli.* We then compared the peak intensities of the oligomers in the same spectrum using Hsp70 expressed in *E. coli* (Hsp70_*E. coli*_) labeled with heavy isotopes (^13^C), separating it by mass from Hsp70 expressed in Sf9 cells (Hsp70_Sf9_). A 1:1 mixture of the two proteins at a high protein concentration (8 μM) under experimental parameters designed to preserve non-covalent interactions (Hernández and Robinson, 2007) reveals dimerization (Figure 1A). Interestingly the mass spectrum shows that for the Hsp70_Sf9_:Hsp70_*E. coli*_ mixture, the Hsp70_Sf9_ dimer has higher intensity than its *E. coli*-expressed counterpart, implying that the Hsp70_Sf9_ dimer is more stable than Hsp70_*E. coli*_ (Figure 1B). Given that the amino acid sequences of Hsp70_Sf9_ and Hsp70_*E. coli*_ are identical, but expression in *E. coli* does not allow for PTMs, we hypothesized that this enhanced interaction was due to acetylation and phosphorylation, which often combine to stabilize subunit interfaces (van Noort et al., 2012). We first investigated the occurrence of acetylation in Hsp70_Sf9_ and identified seven lysine acetylation sites, two of which had been reported previously (Table S1). We then applied a phosphopeptide enrichment strategy and found a phosphosite (T504) in Hsp70_Sf9_, at a known phosphorylation hotspot (Beltrao et al., 2012). This site is not phosphorylated in Hsp70_*E. coli*_ and, together with the multiple acetylation sites, provides a plausible rationale for the enhanced stability of the Hsp70_Sf9_ dimer.

As this particular phosphosite is highly conserved in different eukaryotic species (Beltrao et al., 2012), we probed its significance for dimerization and incubated the Hsp70_Sf9_ dimer with a phosphatase. We prepared two aliquots containing a 1:1 solution of Hsp70_Sf9_ and ^13^C -labeled Hsp70_*E. coli*_ (one with buffer and one with phosphatase). Following overnight incubation without phosphatase, Hsp70_Sf9_ retained a population of dimers (Figures 1B and 1C). Interestingly, a mixed heterodimer, Hsp70_Sf9_:Hsp70_*E.coli*_, formed under these incubation conditions, consistent with subunit exchange occurring within a 16 hr timescale. By contrast, no Hsp70_*E. coli*_ homodimer was observed, supporting enhanced dimerization of Hsp70_Sf9_. Peak splitting of monomeric Hsp70 (Figures 1A and 1C) was attributed to *apo* and nucleotide-bound forms of the proteins, implying loss of nucleotide and ATP turnover during the overnight incubation. The second aliquot, to which phosphatase was added, showed peak splitting due to the loss of nucleotide, but importantly, no dimers were observed (Figures 1B and 1C). To confirm that the Hsp70_*E. coli*_ dimer was not affected by incubation with phosphatase, we repeated these experiments at a higher Hsp70_*E. coli*_ concentration under which dimerization occurs. We found that the dimer was stable for 48 hr with phosphatase present (Figures S1A and S1B). We conclude that since the Hsp70_Sf9_:Hsp70_*E. coli*_ heterodimer and Hsp70_Sf9_ homodimer are weakened by addition of phosphatase, the phosphorylation site in Hsp70_Sf9_ not only stabilizes the homodimer but also promotes formation of the heterodimer.

Given the presence of PTMs and their potential role in stabilizing the dimer interface, we anticipated that the Hsp70_Sf9_ dimer interface might be affected by changes in ionic interactions. Therefore, we recorded mass spectra for solutions containing 2 μM Hsp70_Sf9_, 0.5 μM Hsp40, and 200 μM ATP with increasing ionic strength from 50 to 300 mM potassium chloride (Figure S2). The results show a decrease in the population of dimers at higher ionic strength, consistent with ionic interactions maintaining the dimer interface.

To confirm the proposed role of T504 in enhancing the dimer interface, we prepared a phosphomimic mutant, replacing T504 in Hsp70_*E. coli*_ with a glutamic acid residue to form a T504E variant. A comparison of the mass spectra of the labeled wild-type Hsp70_*E. coli*_ with those of the Hsp70_*E. coli,*T504E_ variant shows an increase in the population of dimers for the phosphomimic when examined in a 1:1 ratio (Figure S1C). Together, these results show that the Hsp70_Sf9_ dimer interface is strengthened by ionic interactions and that a key phosphosite, supported by multiple acetylation sites, contributes to its stability.

### Chemical XL Defines an Antiparallel Hsp70 Dimer

We defined the interface of the Hsp70_Sf9_ dimer by XL with bis(sulfosuccinimidyl)suberate (BS3) first in the presence of excess ATP. SDS-PAGE analysis confirmed the presence of monomers and crosslinked dimers, showing that populations of dimers formed under these conditions (Figure S3A). Gel bands assigned to the monomer and dimer were then subjected to tryptic digestion and liquid chromatography-tandem MS (LC-MS/MS) analysis. We found 170 potential XLs by a database search. We manually validated 154 of these XLs, giving a false-discovery rate (FDR) of 9.61% (Table S2).

We considered a number of high-resolution structures for generation of homology models, including one in which the SBD binds to the hydrophobic linker (Chang et al., 2008) and the *Bos taurus* Hsc70 structure (Jiang et al., 2005). We also considered a model generated from *Geobacillus kaustophilus* (Chang et al., 2008, Wu et al., 2012). This model shows Hsp70 oligomerization via the SBD of one Hsp70 molecule binding to the flexible linker of a second Hsp70 (Figure S4A; Wu et al., 2012). To investigate this dimerization mechanism, we generated a substrate-binding-deficient variant of Hsp70_*E.coli*_ (V438F), which also includes the phosphomimic T504E and was shown to be unable to bind substrates in DnaK and Hsp70 (Mayer et al., 2000, Rohrer et al., 2014). However, the V438F/T504E mutant was able to dimerize despite its substrate-binding deficiency, ruling out the substrate-binding model for dimerization (Figure S4B).

The two high-resolution structures that best accommodate our XLs are the ADP- and ATP-bound states of DnaK (PDB IDs 2KHO and 4B9Q, respectively (Bertelsen et al., 2009, Kityk et al., 2012). We generated a homology model for the ATP state using the structure 4B9Q as a template. For the ADP state, we could not obtain a good homology model, and therefore we manually aligned the human Hsp70 sequence with the solution structure of *E. coli* DnaK (Bertelsen et al., 2009; Figure 1D). Of the multiple intra-subunit XLs derived from the band assigned to the monomer, two were of interest: K190-K507 and K190-K512. Both of these XLs are accommodated better in the ADP state (Bertelsen et al., 2009) than in the ATP state (Kityk et al., 2012). We also observed two intra-XLs consistent with the ATP conformation, but not the ADP state: K159-K512 and K246-K271. The fact that both ATP and ADP conformations are satisfied by our XL restraints implies that ATP hydrolysis takes place, giving rise to the two nucleotide-bound forms that readily interconvert in solution.

Two of the 11 Hsp70 XLs derived from the dimer band, K569-K108 and K561-K108 (both identified with ADP, ATP, or ATPγS) were particularly intriguing. These XLs cannot be assigned to intra-subunit XLs due to the distances required to link the NBD with the lid of the SBD. These crosslinked regions define a dimer interface that can be accommodated in either the ATP or ADP state, and clearly define an antiparallel orientation via interactions between the NBD and SBD of different subunits.

Given that XL provides an average ensemble populated in solution, we need a direct readout of the differences between the two distinct nucleotide-bound conformations of Hsp70 in the dimer. Therefore, using a previously described comparative XL strategy (Schmidt and Robinson, 2014, Schmidt et al., 2013), we added BS3-d_0_ and BS3-d_4_ to ADP- and ATP-containing solutions of Hsp70, respectively. After XL, a 1:1 molar ratio of the two solutions was digested with trypsin prior to LC-MS/MS analysis. This allowed for a quantitative comparison of XLs under different nucleotide conditions (Table S2). We found that in the presence of ADP, the XL K190-K507 was enhanced, defining close interactions in the ADP state. In excess ATP, however, K159-K512 and K246-K271/251 were significantly increased relative to XLs observed in the presence of ADP. These three XLs can only be accommodated in the ATP state. Since it was not possible to obtain XLs exclusive to one conformation (docked or undocked) in the presence of excess ATP, ADP, or ATPγS, and given the likely hydrolysis of ATP, we conclude that the dimer exists in a dynamic equilibrium perturbed by nucleotides but without a single defined conformer.

If we place the PTMs defined above within the context of the antiparallel dimer, then we find that the phosphosite (T504) is close to the hinge region between the SBD β-domain and the SBD α-helical lid, in a lysine-rich pocket that orients it toward the subunit interface for interactions with multiple lysine residues (K561, K567, K568, and K569) in the ATP state (Figure 1D). This network of lysine interactions in the ADP state, close to the phosphosite, could also stabilize the ADP conformation in the antiparallel dimer. The seven acetylation sites identified here also align along the dimer interface in the ATP state, implying that they combine with the phosphosite for signal propagation. These hydrogen-bonding interactions provide a rationale for increased dimerization of Hsp70_Sf9_ and the conservation of the phosphosite (Beltrao et al., 2012), and the correspondence of the amino acid residues involved in acetylation (http://www.uniprot.org) in eukaryotes suggests their functional relevance in vivo.

### Transient Interactions with Hsp40 Promote Hsp70 Dimerization

**Figure 2:**
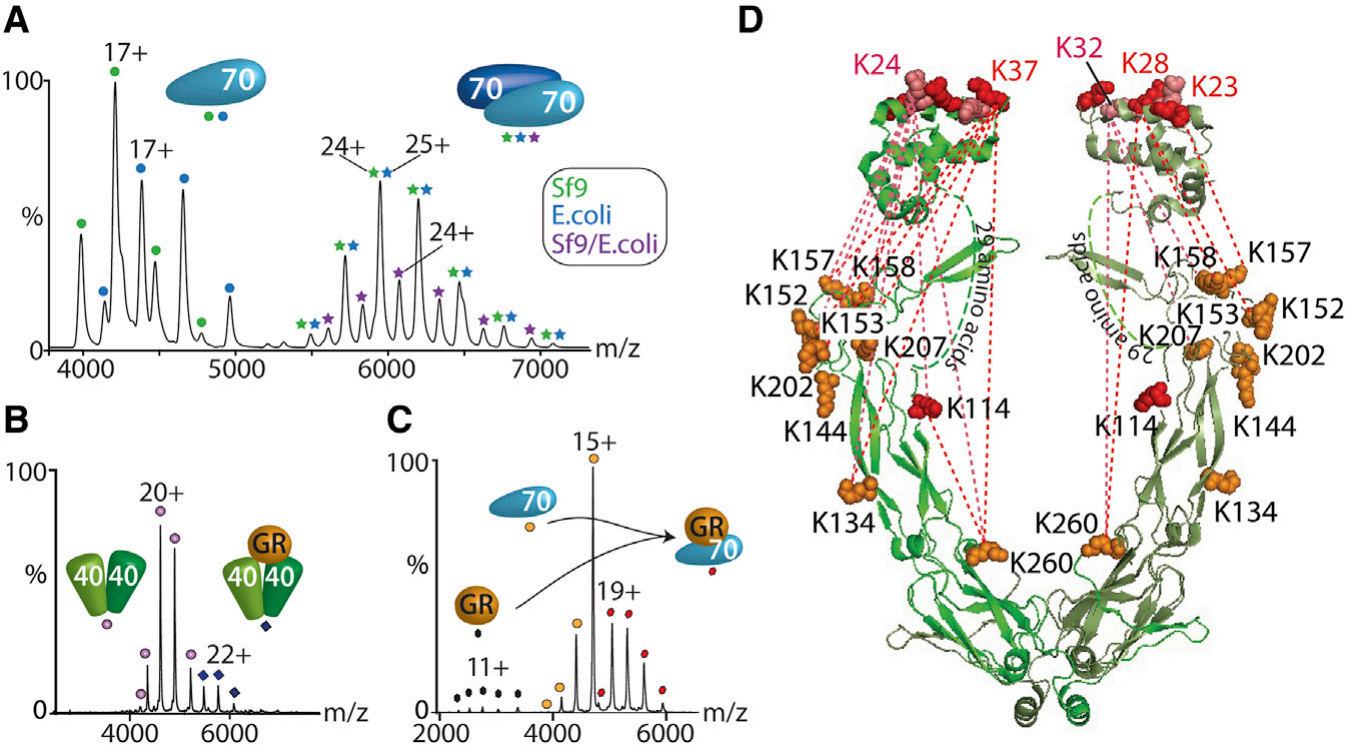
Hsp40 Promotes Interactions with Hsp70 and GR through Its J Domain (A) Mass spectrum of a 1:1 ratio of ^13^C-labeled Hsp70_*E. coli*_ and Hsp70_Sf9_ with natural abundance isotopes in the presence of Hsp40 and ATP. An increase in the population of the Hsp70_Sf9_ dimer and formation of a heterodimer are observed. (B and C) The mass spectrum of GR in the presence of Hsp40 shows only a low population of Hsp40_2_GR (B), but a larger population of Hsp70_*E. coli*_ GR is formed when catalytic amounts of Hsp40 are added (C). Hsp70_*E. coli*_ contains a His-tag. (D) Chemical XL highlights the dynamics of the Hsp40 dimer. Lysine residues in the region of 23–37 in the J domains make multiple interactions with the C-terminal and middle domains of Hsp40. For this symmetrical dimer, we expect XLs to be present on both subunits. For clarity, the two subunits are shown with different XLs. See also Figure S5 and Table S2.

We investigated the effects of Hsp40 on the extent of dimer formation in both Hsp70_*E. coli*_ and Hsp70_Sf9_ using a 1:1 ratio of the two Hsp70s and catalytic amounts of Hsp40 (Figure 2A). In the presence of Hsp40, an increase in the population of the non-covalent dimer was observed for Hsp70_Sf9_ and even for Hsp70_*E.coli*_, albeit at a lower intensity (Figures S5A and S5B). An Hsp70_*E. coli*_:Hsp70_Sf9_ heterodimer was observed as above, consistent with the greater propensity of Hsp70_Sf9_ (compared with Hsp70_*E. coli*_) to form dimers.

To probe potential differences in the dimer interfaces of Hsp70_Sf9_ and Hsp70_*E. coli*_, we employed the comparative XL strategy described above in the presence of excess ATP and cochaperone Hsp40 to enhance dimer formation. To avoid subunit exchange and to compare directly the strengths of the different interfaces, we employed BS3-d_0_ and Hsp70_Sf9_ for Hsp70_*E. coli*_ and BS3-d_4_, respectively, and crosslinked them individually. Equal aliquots of the crosslinked proteins were pooled, digested, and analyzed by LC-MS/MS. We identified 466 XLs after a database search and validated 392 of these manually, giving an FDR of 15.9% (Table S2). Rejecting XLs with peptides of three or fewer amino acids results in 74 XLs (59 Hsp40-Hsp40, 12 Hsp70-Hsp70, and three Hsp40-Hsp70). Differences in subunit interactions can then be related to the extent of dimer formation by changes in the intensity ratio of the inter-protein XLs.

When we compared the intensities of the light and heavy crosslinked peptides, we found that two were noticeably different for the Hsp70_Sf9_ and Hsp70_*E. coli*_ dimers. The K569-K108 (assigned to the inter-subunit XL above) intensity ratio was 5.3:1.0 for the Hsp70_Sf9_ and Hsp70_*E. coli*_ peptides, respectively. This represents a 5-fold increase in the intensity of the crosslinked Hsp70_Sf9_ dimer and is assigned to enhanced interactions in the Hsp70_Sf9_ dimer interface. Interestingly, we identified a second XL, also involving K569 but this time crosslinked to K561, assigned to an intra-XL due to its close proximity. This XL showed a difference in the intensity ratio in the opposite direction, with an increase in Hsp70_*E. coli*_:Hsp70_Sf9_ to 3.4:1.0. Therefore, we conclude that a significant increase in intensity in the XL K569-K561 results from reduced dimer formation in Hsp70_*E. coli*_, thereby promoting intra-molecular XL.

Hsp70 dimerization is significantly enhanced in the presence of sub-stoichiometric quantities of Hsp40, and yet no Hsp40-containing complexes were observed with low Hsp40 concentrations (Figure 2A). This supports the current view of a transient catalytic interaction (Kampinga and Craig, 2010). To address how this transient interaction with Hsp40 enhances dimerization, we first determined the oligomeric state of Hsp40 and observed a dimeric state (Figure S5C; Table S3). Given that Hsp70 and Hsp40 are known to promote client interactions (Kampinga and Craig, 2010), we reasoned that it might be necessary to include a client to stabilize the interactions between them. Using apo GR or apo MBP-GR (GR-fused maltose-binding protein to enhance solubility), both monomeric (Figure S5D), we probed client interactions with Hsp40. Interestingly Hsp40 with GR showed only minimal binding in a 1:1 stoichiometry, independent of the presence of nucleotides (Figure 2B). Incubating Hsp70 with GR in the presence of catalytic amounts Hsp40 and ATP revealed the formation of an Hsp70GR complex (Figure 2C). Significantly, Hsp40 was not incorporated into the Hsp70 complex, even in the presence of the client.

### Dynamic Interactions between Hsp70 and Hsp40

Next, we probed interactions within and between Hsp40 and Hsp70_*E. coli*_ using XL in the presence of GR and nucleotides. To eliminate stabilization by PTMs, we employed Hsp70_*E. coli*_. We identified 57 intra-XLs, 42 of which were assigned to intra-Hsp40 interactions. Aligning the XLs with the structural elements of Hsp40 (Li et al., 2003; Table S4) places many of the interactions between the J domains (K23, K24, K28, K32, and K37) and the adjacent β-sheet region, which is linked by an unstructured 29 amino acid linker (Figure 2D). The very high level of inter-XL observed for Hsp40 (Figure 2D) indicates its open, flexible structure.

**Figure 3:**
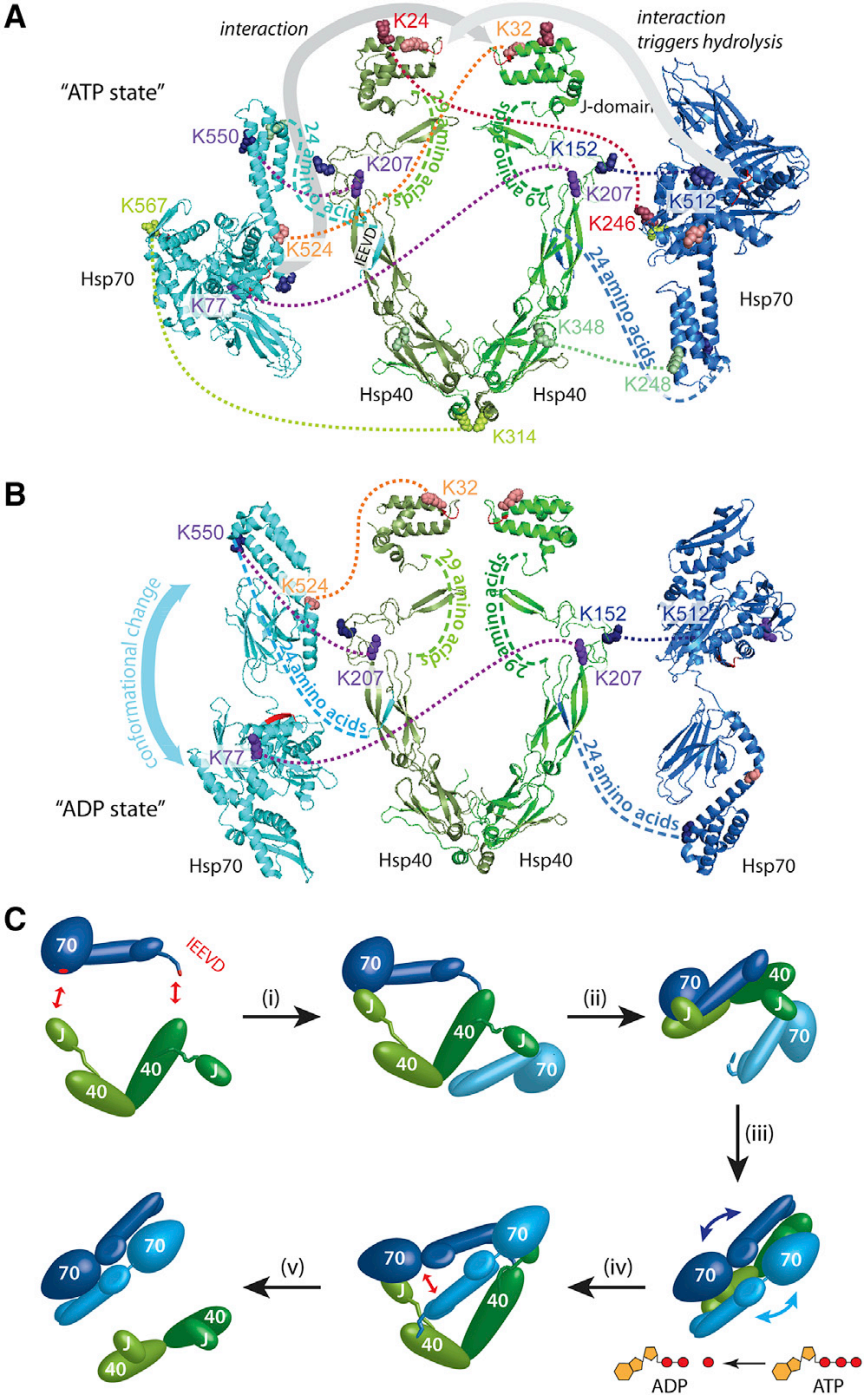
Proposed Dimerization Model of Hsp70 following Interactions with Hsp40 (A) XL reveals seven critical inter-subunit interactions that allow alignment of the Hsp70 dimer in an antiparallel ATP state with Hsp40 (missing C-terminal residues of Hsp70 are indicated). Following stimulation of the ATPase activity of Hsp70 through interactions with Hsp40 J domains, the predominant conformation is likely the undocked ADP state. (B) The same XLs as in (A) can be rationalized in this structure, with the exception of Hsp70:40 XLs 567-314 and 248-348, which are better accommodated in the ATP state of Hsp70. (C) Schematic of the J domains binding Hsp70 to bring the C-terminal IEEVD motif of Hsp70 into close proximity to the binding site on Hsp40, placing Hsp70 in a well-defined position across the Hsp40 dimer. The second Hsp40 J domain can bind to a second Hsp70, positioning it in close proximity to the first Hsp70 and triggering the formation of an antiparallel Hsp70 dimer. Hsp40 stimulates ATPase hydrolysis of Hsp70 and induces a conformational change from the docked to the undocked form. Following Hsp70 dimerization, the Hsp40 dimer dissociates from Hsp70.

Seven inter-XLs define interactions between Hsp40 and Hsp70_*E. coli*_. One XL is formed between the J domain and the Hsp70 SBD (K32-K524). The SBD of Hsp70 is further aligned via K512-K152 in Hsp40. An Hsp40 residue (K207) crosslinks to two distal residues (K77 and K550) on Hsp70 in the NBD and lid, respectively (Figure 3A). These XLs are not compatible with a single Hsp70-Hsp40, but are consistent with one Hsp70 bridging an Hsp40 dimer, allowing interactions with the two K207 residues in the Hsp40 dimer with both the lid and NBD of Hsp70. This is in accord with previous results that locate Hsp40 at the IEEVD motif of Hsp70 and between the Hsp40 J domains and an acidic groove in the NBD of Hsp70 in the ATP state (Qi et al., 2013; Figure 3A).

Given that Hsp40 stimulates the ATPase activity of Hsp70, and the observation of the J domain close to the nucleotide-binding site in Hsp70 (residues 24–246), we also considered Hsp70 in the undocked ADP form. Four XLs can be accommodated with Hsp70 molecules in the ADP conformation (Figure 3B). The fact that a subset of XLs can be accommodated in docked and undocked states of Hsp70 is consistent with the equilibrium that exists in solution. Moreover, the same inter-Hsp70 XLs defined above confirm the antiparallel Hsp70 dimer in both docked and undocked conformations (Table S2).

As a consequence of these interactions, movement is restricted, with two Hsp70 subunits held in an antiparallel orientation for dimerization. Overall, these data provide molecular details of the interactions involved in bringing Hsp70 subunits together, with the J domains of Hsp40 binding to the NBD of Hsp70, stimulating ATPase activity and inducing conformational changes necessary to prime Hsp70 for substrate binding (Figure 3C).

### Antiparallel Dimers of Hsp70 Facilitate Client Transfer to Hsp90

**Figure 4:**
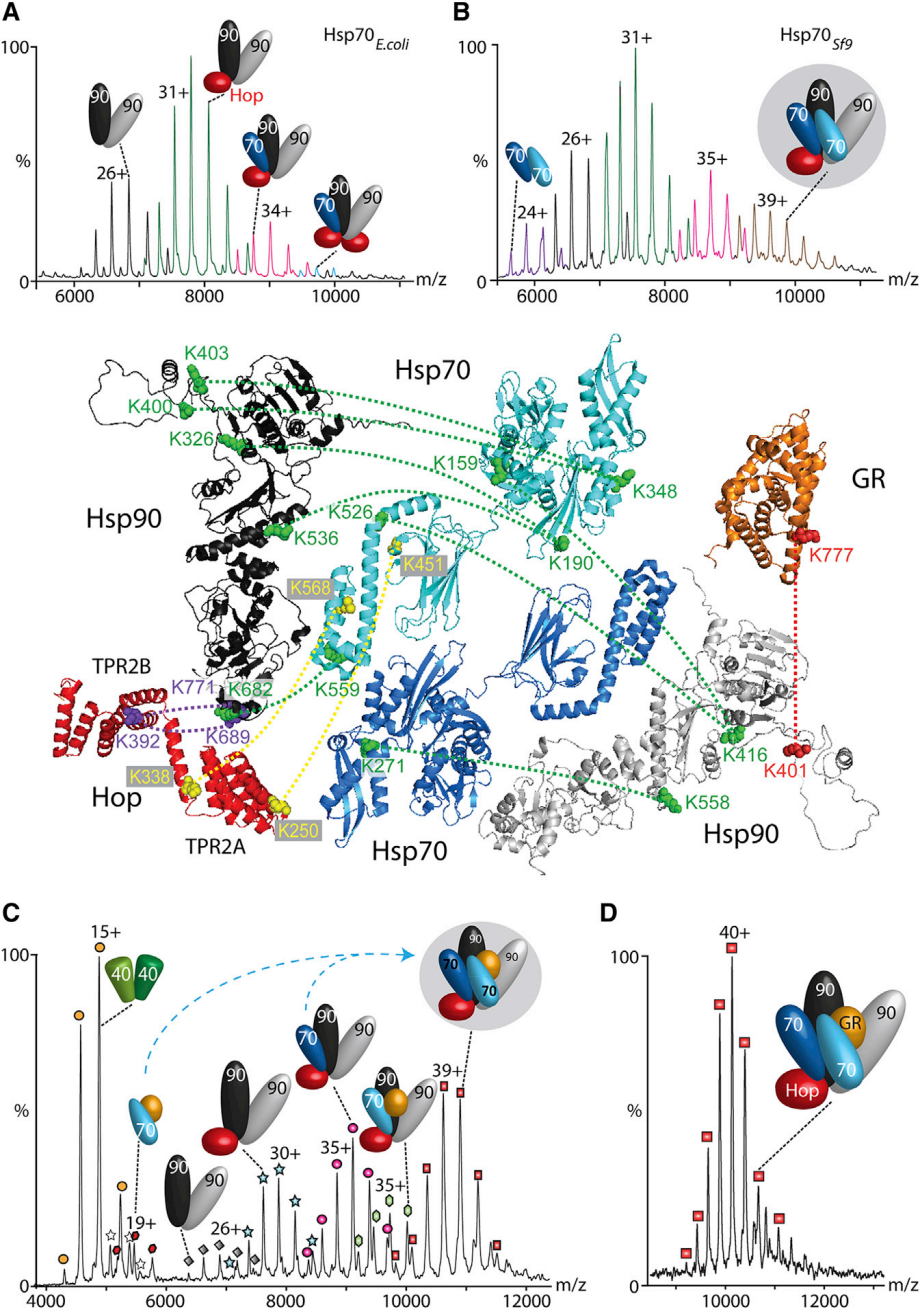
Hsp70 Binds Hsp90, GR, and Hop to Form a Highly Stable Heterohexamer (A and B) Mass spectra of solutions containing Hsp90 and Hop interacting with either Hsp70_*E. coli*_ (A) or Hsp70_Sf9_ (B), respectively. In the absence of Hsp40, a larger population of the Hsp90_2_HopHsp70_2_ complex is formed for the Sf9 protein. (C) With catalytic amounts of Hsp40 and one molar equivalent of Hsp70_*E. coli*_, a hexameric complex containing client protein is formed. (D) When the molar equivalence of Hsp70 is increased, in line with the Hsp90 dimer, a stable client-loading complex is formed. XLs define an Hsp90-Hsp70 interface and additional XLs locate Hop, Hsp70, and Hsp90. Only one XL was observed for GR binding to Hsp90, attributed to its protected position within the client-binding cleft (central panel). See also Figure S3 and Tables S2 and S3.

A key question prompted by the antiparallel arrangement of Hsp70 subunits is whether they are an integral part of chaperone complexes involving Hsp90. To address this question, we compared the effect of PTMs on Hsp70_Sf9_ versus Hsp70_*E. coli*_ on interactions with Hop and Hsp90 in the absence of Hsp40. We found that the predominant heterocomplex for Hsp70_*E. coli*_ is Hsp90_2_Hop, with only a low incorporation of Hsp70 monomer, and a second species at very low intensity containing an additional Hop, Hsp90_2_Hsp70Hop_2_ (Figure 4A). It was not possible to form complexes containing more Hsp70_*E. coli*_ than Hop subunits. For Hsp70_Sf9_, there is clear evidence that two molecules of Hsp70_Sf9_ were incorporated into the complex to form Hsp90_2_Hsp70_2_Hop, indicating that Hsp70_Sf9_ is likely incorporated as a dimer with only one Hop (Figure 4B). The formation of this complex is in accord with our earlier proposal that PTMs in Hsp70_Sf9_ promote dimerization. This holds true even in the absence of Hsp40, in complexes with Hsp90 and Hop.

To determine whether Hsp70 dimerization plays a role in forming the client-loading complex, we investigated interactions with GR. We saw no evidence of direct binding of GR to Hsp90_2_Hop, and therefore formed the Hsp70GR complex in the presence of Hsp40, as above, prior to incubation with the Hsp90_2_Hop complex. With catalytic quantities of Hsp40 and equimolar ratios of Hsp90, Hop, Hsp70, and GR, we observed two new complexes, Hsp90_2_Hsp70HopGR and Hsp90_2_Hsp70_2_HopGR, with the complex containing two Hsp70 molecules being predominant (Figure 4C). Less intense charge-state series were observed for complexes without the full cohort of subunits and assigned to intermediates populated during the assembly process. Our results confirm that pre-binding of GR to Hsp70 is favored over dimerization of Hsp70 in the presence of Hsp40, and is a prerequisite for binding GR to Hsp90. Since binding of monomeric Hsp70 to Hsp90_2_Hop is also favored, however, the observation that the predominant GR-chaperone complex incorporates two Hsp70s implies that dimerization of Hsp70 plays a major role in client binding to the Hsp90 complex.

To test this hypothesis, we increased the concentration of Hsp70 such that two Hsp70s were present per Hsp90_2_. Under these conditions, and after forming the Hsp70GR complex in the presence of Hsp40, we found that Hsp90_2_Hsp70_2_HopGR was formed almost exclusively (Figure 4D). No other sub-stoichiometric complexes were formed under these conditions. The observation of this highly stable complex, incorporating two copies of Hsp70 with Hop and Hsp90_2_ together with a client, suggests that this is an important mechanistic step in priming the later stages of the cycle with a complex predisposed to transfer the client from Hsp70 to Hsp90.

To define the location of subunits within the client-loading complex, we performed XL experiments using BS3-d_0_ and BS3-d_4_. To reduce complexity, we separated the crosslinked complexes by SDS-PAGE prior to digestion and LC-MS/MS analysis (Figure S3B). We identified 679 XLs from all protein bands after a database search and validated 366 of these manually, giving an FDR of 46.1%. Rejecting XLs for peptides with fewer than four amino acids yields 102 unique XLs. Disregarding intra-XLs, this leaves 31 inter-XLs. In the absence of high-resolution structures for human Hsp90 and Hop, we generated homology models using Swiss-Model (Table S4). Given that there are no PDB entries for full-length Hop, we used a yeast template comprising the TPR2A- and TPR2B-binding sites, which are known to bind the EEVD motif of Hsp70 and the MEEVD motif of Hsp90, respectively (Scheufler et al., 2000). We then assembled models of the interacting subunits and used these to display the XL restraints (Figure 4, main panel).

Five XLs locate the N-terminal and middle domains of Hsp90 in close proximity to the NBD (K326-K190, K416-K159, K558-K271, and K536-K190) and SBD (K416-K526) of Hsp70. One additional XL between the C-terminal regions of both proteins (K689-K559) defines the vertical alignment. Strikingly, we observed an additional XL between Hsp70 and Hsp90 (K271-K558) that cannot be reconciled with only one Hsp70, and can only be rationalized by antiparallel subunit interactions of a second Hsp70. Two XLs formed between Hop and Hsp90 (K392-K689/771) support the positioning of Hop with upward- and downward-facing TPR2B- and TPR2A-binding motifs, respectively, as suggested previously (Schmid et al., 2012), allowing for simultaneous binding of Hsp90 and Hsp70. Therefore, these 14 XLs confirm earlier findings of an antiparallel Hsp70 dimerization.

In our study, only one XL was found to link Hsp90 and GR, positioning the GR in close proximity to Hsp90. This XL (K401-K777) connects an unstructured linker of Hsp90 to the C-terminal residue of GR. However, given the location of the client (sequestered within a binding-site cleft formed from two copies of both Hsp90 and Hsp70), access to XL reagents is likely to be restricted. According to our results, GR is located in close proximity to the SBD of Hsp70 and the middle domain of Hsp90. This active-site “cleft” for client binding is positioned for interaction with further cochaperones and for transfer of the client from Hsp70 to Hsp90.

**Figure 5:**
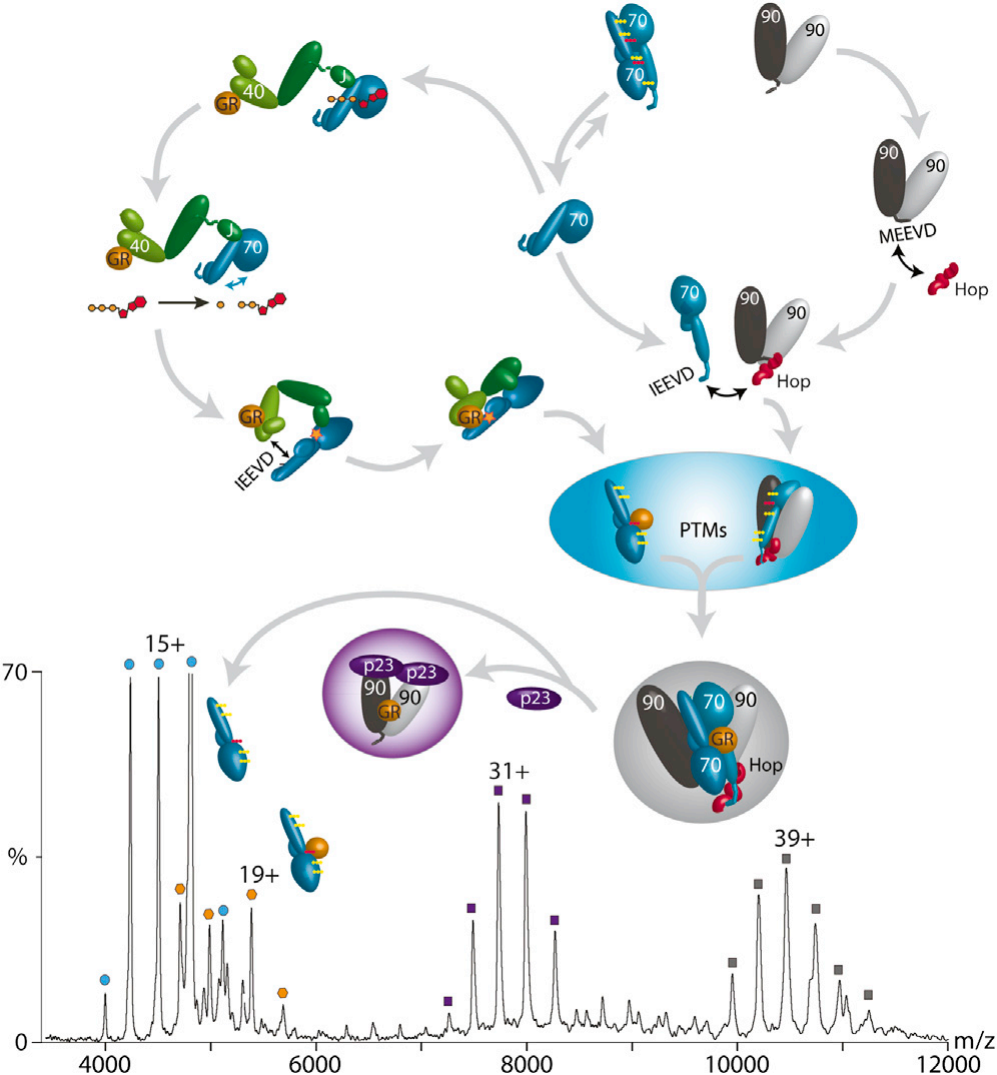
Joining of the Hsp70 and Hsp90 Cycles, and Addition of p23 to the Client-Loading Complex Hsp40 promotes client transfer to Hsp70, which exists in a monomer-dimer equilibrium. One Hsp40 binds to GR, while the J domain of the second Hsp40 associates with an Hsp70 monomer. This stimulates ATPase activity and induces a conformational change from the docked to the undocked state. Hsp90 binds to Hsp70 via Hop. Hsp40 binding one Hsp70 can then bind the second Hsp70 to form the antiparallel dimer, which is stabilized by PTMs. The joining of the two cycles is mediated by Hsp40 binding only transiently to catalyze the Hsp70 dimerization. Formation of the stable heterohexameric client-binding complex Hsp90_2_Hsp70_2_HopGR with antiparallel Hsp70 subunits primes the client for transfer to Hsp90. Subsequent interactions with p23, Aha, or immunophilins promote GR maturation and transport to the nucleus. The mass spectrum shows the effect of the addition of p23 to the client-loading complex. An Hsp90_2_GRp23_2_ complex is formed as Hop and Hsp70 are released, thus completing the transfer of GR to Hsp90. See also Tables S2 and S3.

To test whether this cleft is predisposed for handover of GR, we added stoichiometric amounts of the cochaperone p23 (Figure 5). Mass spectra revealed the formation of a new complex assigned as Hsp90_2_p23_2_GR. Interestingly, no Hop or Hsp70 remained in this complex. Therefore, our experiments reconstructed the ATP-driven transfer reaction of GR from Hsp70 to Hsp90, facilitated by the client-loading complex and effected by addition of the cochaperone p23.

## Discussion

We have shown that formation of the client-loading complex involves prior binding of Hsp40 and the client to Hsp70, both of which stimulate ATP hydrolysis to place monomeric Hsp70 in the undocked client-binding conformation. Binding of GR to Hsp40, and Hsp70 to the GR-bound Hsp40 dimer, positions GR and Hsp70 in close proximity, forming an Hsp70GR complex devoid of Hsp40. XL reveals that the Hsp40 dimer contacts an Hsp70 in two locations: (1) via its J domain with the acidic groove between the Hsp70 SBD and NBDs, as shown previously (Hennessy et al., 2005), and (2) close to K207 of the second Hsp40 molecule with the IEEVD binding motif of Hsp70. Up to two Hsp70 subunits can bind to an Hsp40 dimer in this way (Figure 5).

The fact that the Hsp70/Hsp40 heterotetramer is not observed and the cellular concentration of Hsp40 is significantly lower than that of Hsp70 implies that this complex has limited stability or is formed only transiently, as proposed previously (Young, 2010). Although a catalytic role of Hsp40 is widely recognized, the Hsp70 dimerization, binding across the two symmetric faces of Hsp40, provides new mechanistic insight. The effect of this binding is to orient the two Hsp70 subunits in such a way that an antiparallel dimer is formed, as evidenced by XL experiments and in agreement with a recent report of DnaJ, the bacterial homolog of Hsp40, and RepE forming a DnaJ_2_RepE_2_ complex (Cuéllar et al., 2013). In addition, antiparallel dimers of DnaK were shown to be important for interaction with Hsp40 (Sarbeng et al., 2015). However, our XL results offer the first molecular details of antiparallel Hsp70 dimerization catalyzed by Hsp40.

Enhanced dimerization of Hsp70 expressed in Sf9 cells instead of *E. coli* was unexpected and attributed to the presence of key PTMs in Hsp70_Sf9_. This is supported by several experiments in which (1) dimer interactions were abolished during dephosphorylation, (2) increasing ionic strength disrupted the dimer interface, (3) multiple acetylation sites aligned along the interface, and (4) a phosphomimic mutant strengthened the dimer interface.

Antiparallel Hsp70 dimers have been reported in X-ray structures of the related yeast protein Hsp110 (Liu and Hendrickson, 2007) and for the *E. coli* protein DnaK (Qi et al., 2013), although rotation around the interface axis was required to satisfy our XL constraints in these structures. It is also noteworthy that we used a DnaK template in which the ATPase activity was abolished and modifications to prevent self-association were introduced. Therefore, the differences between our model and the X-ray structure could arise from the heterogeneity of ATP/ADP-bound states in solution or from the use of the full-length wild-type Hsp70.

We also considered a model involving recognition of the inter-domain linker by the SBD of a second Hsp70 (Figure S4A). Employing a substrate-binding-deficient mutant, we found that dimerization was not impaired, confirming that dimerization does not occur through the flexible linker. The sensitivity to ionic strength (Figure S2) and the fact that dimerization occurs for both undocked and docked states excludes associations in which the inter-domain linker is accessible.

We propose that Hsp70 plays a key role in the Hsp70/Hsp40/GR chaperone system. The transient interactions of Hsp40 with Hsp70 “prime” it for interaction either with a second subunit (Figure 3C) or with GR (Figure 5). A recent study showing that dimerization of Hsp70 is important for interactions with Hsp40 supports this proposal (Sarbeng et al., 2015). The absence of Hsp70_2_GR complexes implies that the substrate and interface-binding sites are mutually exclusive. During the formation of the final client-loading complex defined here, Hsp90_2_Hsp70_2_HopGR, the Hsp40 dimer orients the Hsp70GR complex onto the second Hsp70 within the Hsp90_2_Hsp70Hop complex. We propose that it is this Hsp70 dimerization event, mediated by Hsp40, ATP, Hsp90, and Hop, that provides the driving force to join the two cycles (Figure 5).

The observation of an Hsp90_2_Hsp70_2_HopGR complex was at first surprising given early reports that only one Hsp70 is incorporated into the Hsp90/Hop complex (Dittmar et al., 1997, Pratt and Dittmar, 1998). Nonetheless, recent reports of Hsp90_2_Hsp70HopGR complexes in EM studies and our own data confirm that such a species can also form. The client-loading complex is remarkably stable, and this stability likely arises from the fact that the Hsp90_2_Hsp70_2_ heterotetramer is stabilized not only by Hop but also by GR bridging, as revealed by XL. The stability of this complex is likely necessary to protect client proteins prior to their transfer to Hsp90, as supported by recent EM studies (Kirschke et al., 2014), XL of Hsp90GR, and disassembly of the complex when challenged with p23.

Considering that chaperone concentrations are increased in various diseases, with Hsp70 being twice as high as Hsp90 (Kundrat and Regan, 2010), and an enhanced PTM status being linked to cancer (Dutta et al., 2013), an antiparallel dimer becomes relevant. However, the transient nature of this Hsp70 dimer suggests that it plays a role in larger complexes. The high stability of Hsp90_2_Hsp70_2_HopGR implies that it serves as a holding place, since it is rapidly disassembled by interactions with p23.

The joining of the two cycles through Hsp70 dimerization suggests novel therapeutic approaches. Inhibition of Hsp90 interactions plays a role in cancer therapy (Barrott and Haystead, 2013), and maintaining high levels of Hsp70/Hsp40 is thought to protect against aggregation diseases such as Huntington’s (Schaffar et al., 2004). Disrupting crucial interactions between these two cycles could therefore maintain high levels of Hsp70/Hsp40 while enabling inhibition of Hsp90. In this regard, signal propagation between the key acetyl and phospho sites could fine-tune the interface dynamics at the intracellular level. Preventing PTMs of the critical amino acids in the Hsp70 dimer interface could compromise formation of the client-loading complex. As such, inhibiting the respective kinase or acetylase could help regulate interactions within this vital interface, and therefore constitutes a promising avenue for therapeutic intervention.

## Experimental Procedures

### Proteins

All protein sequences were human except for Hsp40, which was from yeast. Proteins were overexpressed in *E. coli* with an N-terminal His-tag, except when stated otherwise, and purified as previously described (Southworth and Agard, 2011). ^13^C-labeled Hsp70_*E. coli*_ and ^15^N-labeled Hsp70_*E. coli*_ were expressed in *E. coli* using M9 media. The GRLBD construct (residues 521–777) contained a phenylalanine F602S mutation to enhance solubility. MBP-GR was used where noted to improve the quality of MS spectra. GR constructs were expressed and purified in the presence of dexamethasone followed by extensive dialysis to remove the ligand. p23 was purified as previously described (McLaughlin et al., 2006). Yeast Hsp40 and the GRLBD construct are referred to as Hsp40 and GR, respectively.

### Assembly of Hsp90-Client Complexes

Protein complexes were assembled in binding buffer (30 mM HEPES, 50 mM KCl, 2 mM dithiothreitol, pH 7.5). Individual proteins were analyzed in 100 mM ammonium acetate (AmAc; pH7.5). Binary complexes were formed at 1 μM final concentration unless otherwise stated. Nucleotides were added to a final concentration of 200 μM.

Ternary complexes were formed in binding buffer. Hsp70 and GR were added at equimolar concentrations of 1 μM to a solution containing 0.3 μM Hsp40 and 200 μM ATP-Mg, followed by incubation at room temperature for 45 min. Hsp90/Hsp70/Hop/GR complexes were assembled by incubating Hsp90, Hop, Hsp70, and GR, each at 1 μM with 0.3 μM Hsp40. For MS, the buffer was exchanged to 100 mM AmAc, pH 7.5, using micro Bio-Spin columns (Bio-Rad Laboratories) or Amicon 10 kDa MWCO (Millipore).

### MS of Intact Complexes

Spectra were acquired on a QToF II mass spectrometer (Waters) modified for high mass transmission (Sobott et al., 2002). Then, 2.5 μl of the solution was introduced into the mass spectrometer using a gold-coated capillary needle prepared in house (Hernández and Robinson, 2007). Spectra were acquired in the positive ion mode and MS conditions were kept constant while concentration effects were monitored. For instrument parameters, see the Supplemental Experimental Procedures. Spectra were processed with MassLynx V4.1 with minimal smoothing and analyzed using *Mass*ign (Morgner and Robinson, 2012) and Unidec (Marty et al., 2015) software.

### Phosphatase Treatment of Hsp70

A 1:1 mixture of 3 μM ^13^C-labeled Hsp70_*E. coli*_ and Hsp70_Sf9_ with natural abundance isotopes was incubated with and without phosphatase (alkaline calf intestine phosphatase on agarose beads; Sigma-Aldrich) in 100 mM AmAc (pH 7.5), at 4°C for 16 hr. Phosphatase beads were removed by filtration.

### Ionic Strength Titration

Hsp70_Sf9_ (2 μM) was incubated with 0.5 μM Hsp40 and 200 μM ATP/Mg^2+^ in binding buffer supplemented with 50, 100, or 300 mM KCl for 1 hr at room temperature. The buffer was exchanged to 100 mM AmAc for MS analysis.

### Tryptic Digestion

Proteins were digested with Trypsin in gel as previously described (Shevchenko et al., 1996).

### Phosphopeptide Enrichment

Phosphopeptides were enriched using TiO_2_. Eluted peptides were dried in a vacuum centrifuge and re-dissolved for LC-MS/MS analysis (Supplemental Experimental Procedures).

### Chemical XL

Protein complexes were crosslinked with BS3 in binding buffer. The protein and crosslinker concentrations are listed in Table S2. For experimental details, see the Supplemental Experimental Procedures.

### LC-MS/MS and Database Search

Peptides were separated by nano-flow liquid chromatography and directly eluted into an LTQ-Orbitrap XL hybrid mass spectrometer (Thermo Scientific). Potential XLs and phosphorylated/acetylated peptides were identified by searching the raw data against a database (see the Supplemental Experimental Procedures for details).

### Generation of Models

We arranged all protein structures/homology models in a possible quaternary structure that satisfied the distance constraints of BS3: 11.4 Å; crosslinked lysine side chains: 6.5 Å (twice); and ≈10 Å for conformational dynamics, resulting in ≈35–40 Å as the maximal distance for two crosslinked residues. Homology models for Hsp90, Hsp70, and Hop were generated using the Swiss-Model Workspace (http://swissmodel.expasy.org/) with yeast and *E. coli* templates (Table S4). For other proteins, we used available PDB files (Table S4).

## Author Contributions

V.B.-E. purified isotopically labeled proteins and prepared the V438F/T504E mutant. C.S. performed XL experiments. V.B.-E., I.-O.E., and N.A.P. performed MS experiments. N.M., C.S., V.B.-E., I.-O.E., N.A.P., E.M.C., and C.V.R. analyzed data. E.K., S.D., D.A., and S.E.J. contributed proteins and the phosphomimic variant. N.M. and C.V.R. designed research. N.M., C.S., V.B.-E., and C.V.R. wrote the paper with contributions from all authors.

## Supplemental Information

Document S1. Supplemental Experimental Procedures, Figures S1–S5, and Tables S1, S3, and S4

Table S2. Chemical Crosslinking of Hsp70-Hsp90 Complexes and Subcomplexes, Related to Figures 1, 2, 3, and 4

Document S2. Article plus Supplemental Information
