# Supplementary material for: Hsp70 Forms Antiparallel Dimers Stabilized by Post-translational Modifications to Position Clients for Transfer to Hsp90
Source: Cell Rep. 2015 Apr 23;11(5):759–69. doi: 10.1016/j.celrep.2015.03.063 (PMC4431665; doi:10.1016/j.celrep.2015.03.063)
Supplement: Document S1. Supplemental Experimental Procedures, Figures S1–S5, and Tables S1, S3, and S4 [file mmc1.pdf]

Cell Reports

Supplemental Information

Hsp70 Forms Antiparallel Dimers Stabilized by Post-translational Modifications to Position Clients for Transfer to Hsp90

Nina Morgner, Carla Schmidt, Victoria Beilsten-Edmands, Ima-obong Ebong, Nisha A. Patel, Eugenia M. Clerico, Elaine Kirschke, Soumya Daturpalli, Sophie E. Jackson, David Agard, and Carol V. Robinson

Supplemental Figures

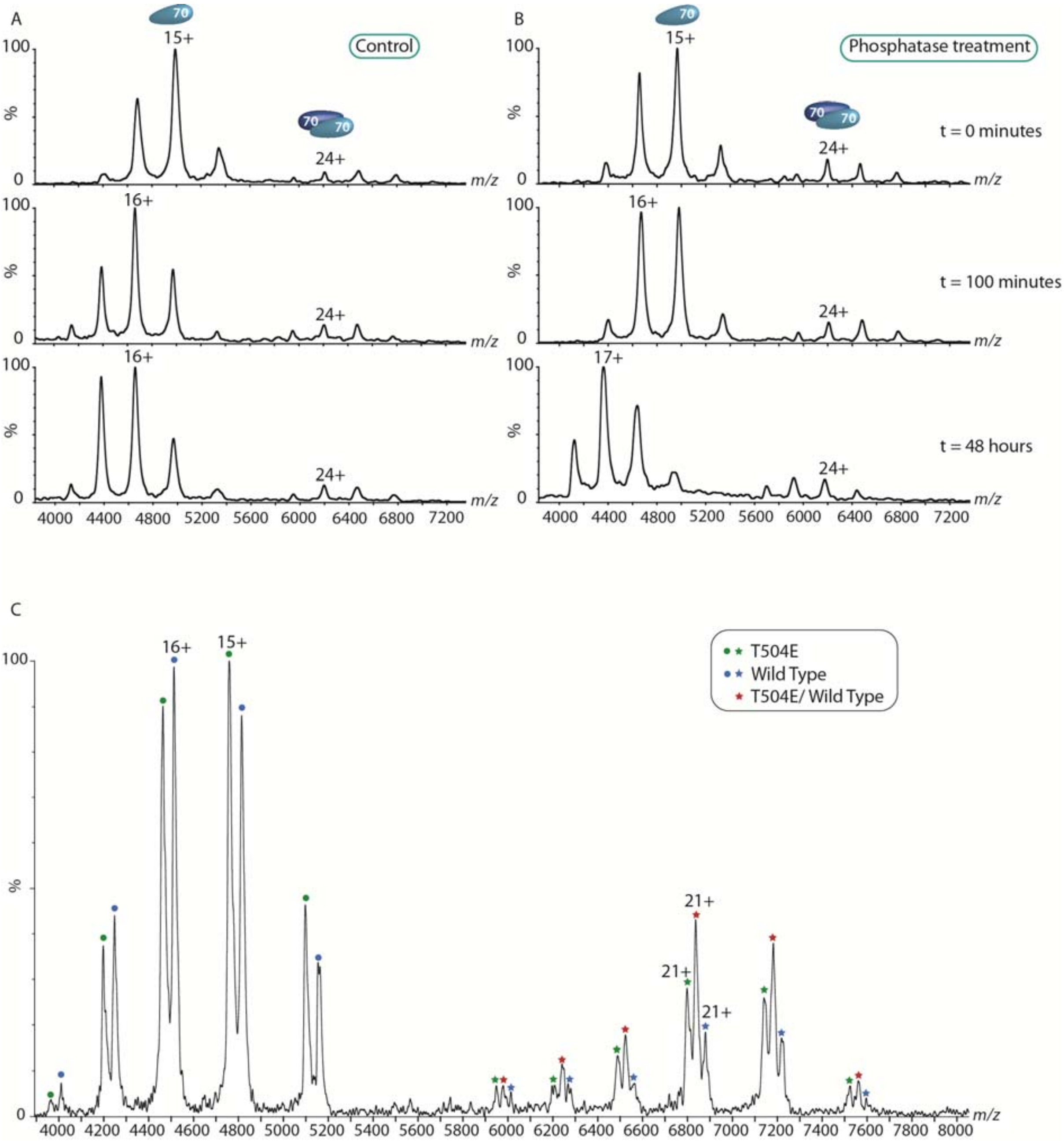

Figure S1 (related to Figure 1): Phosphatase treatment of Hsp70$_{E.\ coli}$ and dimerization of the phosphomimic variant of Hsp70$_{E.\ coli}$ T504E. Phosphatase treatment does not reduce dimerization in Hsp70$_{E.\ coli}$ over a 48 hour time period. 6 µM ^{13}C-labeled wild-type Hsp70$_{E.\ coli}$ incubated without phosphatase **(A)** or in the presence of phosphatase **(B)**. **(C)** The T504E variant was incubated in a 1:1 ratio with the ^{15}N-labeled wild type protein in the presence of ATP to compare directly the intensities of the Hsp70-dimer. The phosphomimic variant T504E shows a greater proportion of dimer compared to the wild type protein.

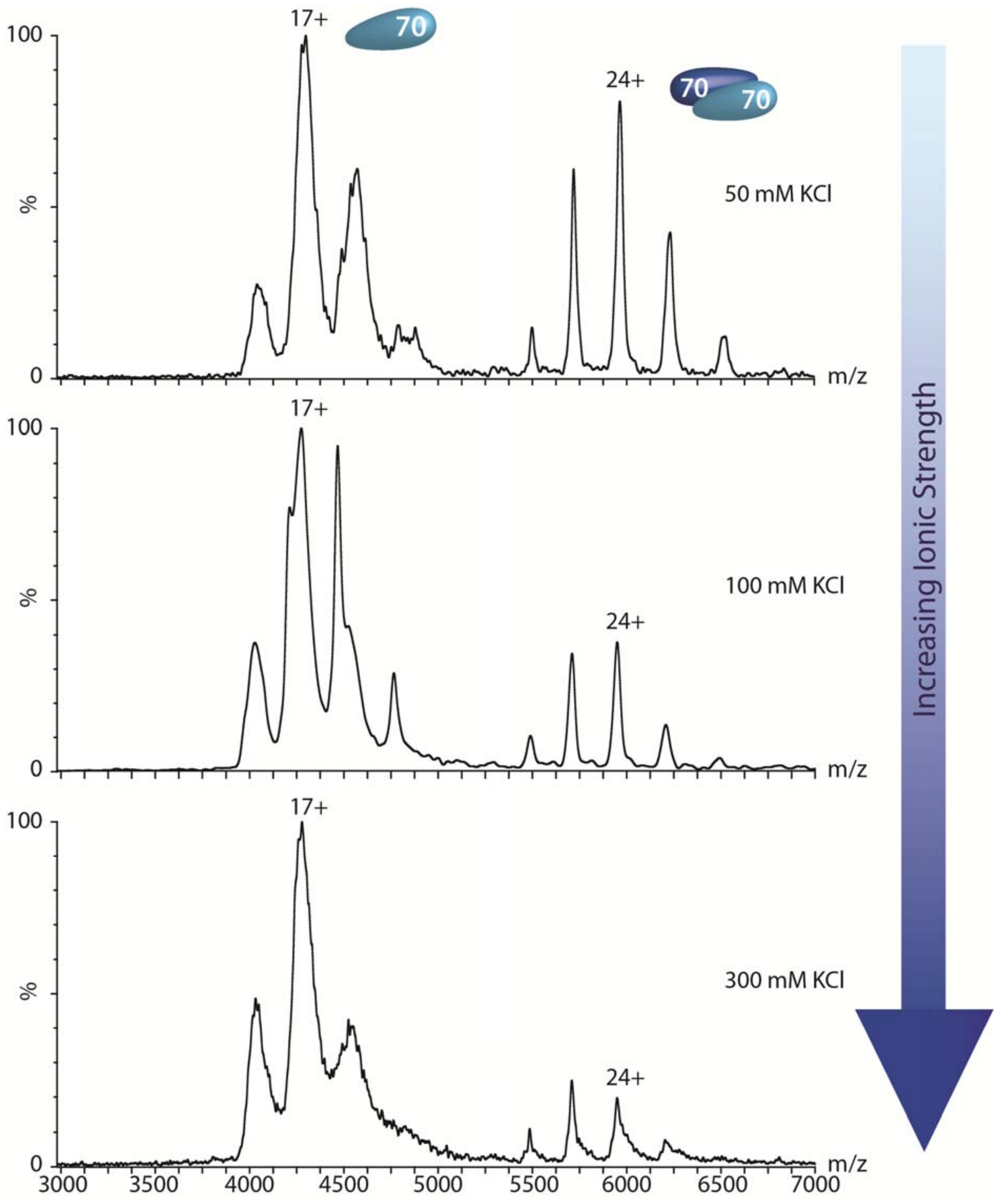

Figure S2 (related to Figure 1): The Hsp70-dimer is sensitive to increasing ionic strength. 2 µM Hsp70$_{Sf9}$ with 0.5 µM Hsp40 and ATP was analysed by mass spectrometry from three different solution conditions containing different ionic strength of the binding buffer (from 50 mM to 300 mM KCl). The mass spectra were recorded at high pressure to promote dimer formation. The intensity of the Hsp70-dimer peaks decreases at higher ionic strength.

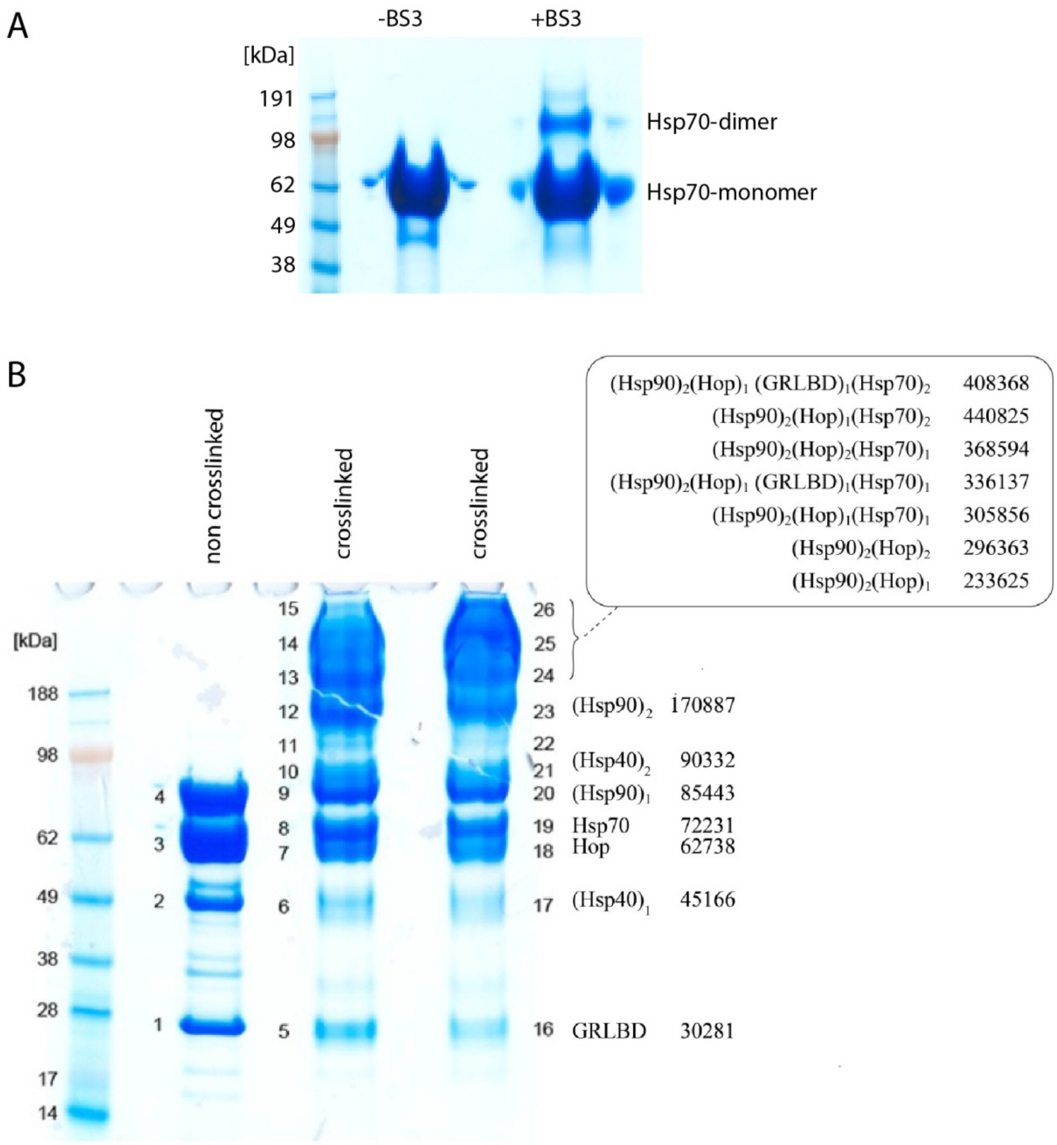

Figure S3 (related to Figures 1 and 4): SDS-PAGE of dimeric Hsp70$_{Sf9}$ and the client-loading complex. (A) Hsp70$_{Sf9}$ was incubated with or without BS3-d0/d4 and analysed by SDS-PAGE. The gel shows monomeric Hsp70$_{Sf9}$ in the control sample (-BS3) and monomeric and dimeric Hsp70$_{Sf9}$ in the cross-linked sample (+BS3). **(B)** Two different concentrations of BS3 cross-linker were used. Additional protein bands are visible after crosslinking. Bands 1-26 were excised for LC-MS/MS and cross-linked peptides were identified as described. Protein masses in Da are given for single protein subunits* as well as possible (sub-) complexes.

*Hsp70$_{E. coli}$ was used for this experiment.

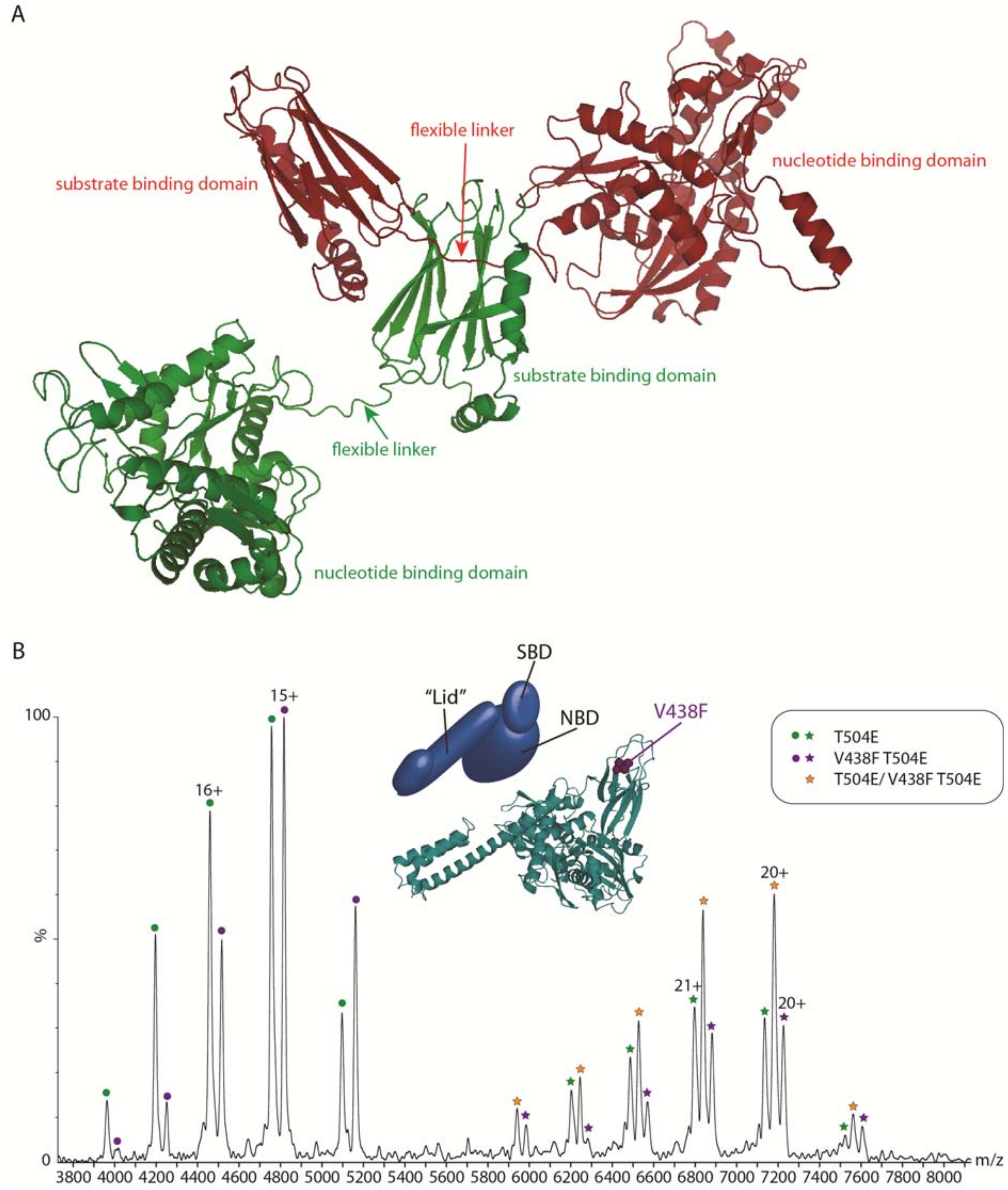

Figure S4 (related to Figure 1): Dimerization of Hsp70 in a substrate-like fashion and dimerization of the substrate binding-deficient variant of Hsp70$_{E.\ coli}$ V438F T504E. (A) One Hsp70 (green) is binding the linker between substrate and nucleotide binding domain of a second Hsp70 (red). The second Hsp70 (red) thus represents a substrate. PDB ID 4ANI (Wu et al., 2012). **(B)** The T504E variant was incubated with the ^{15}N-labeled V438F/T504E variant in the presence of ATP to compare directly the intensities of the Hsp70-dimer. The substrate binding-deficient variant dimerizes and this dimerization is at a similar level compared with the phosphomimic variant.

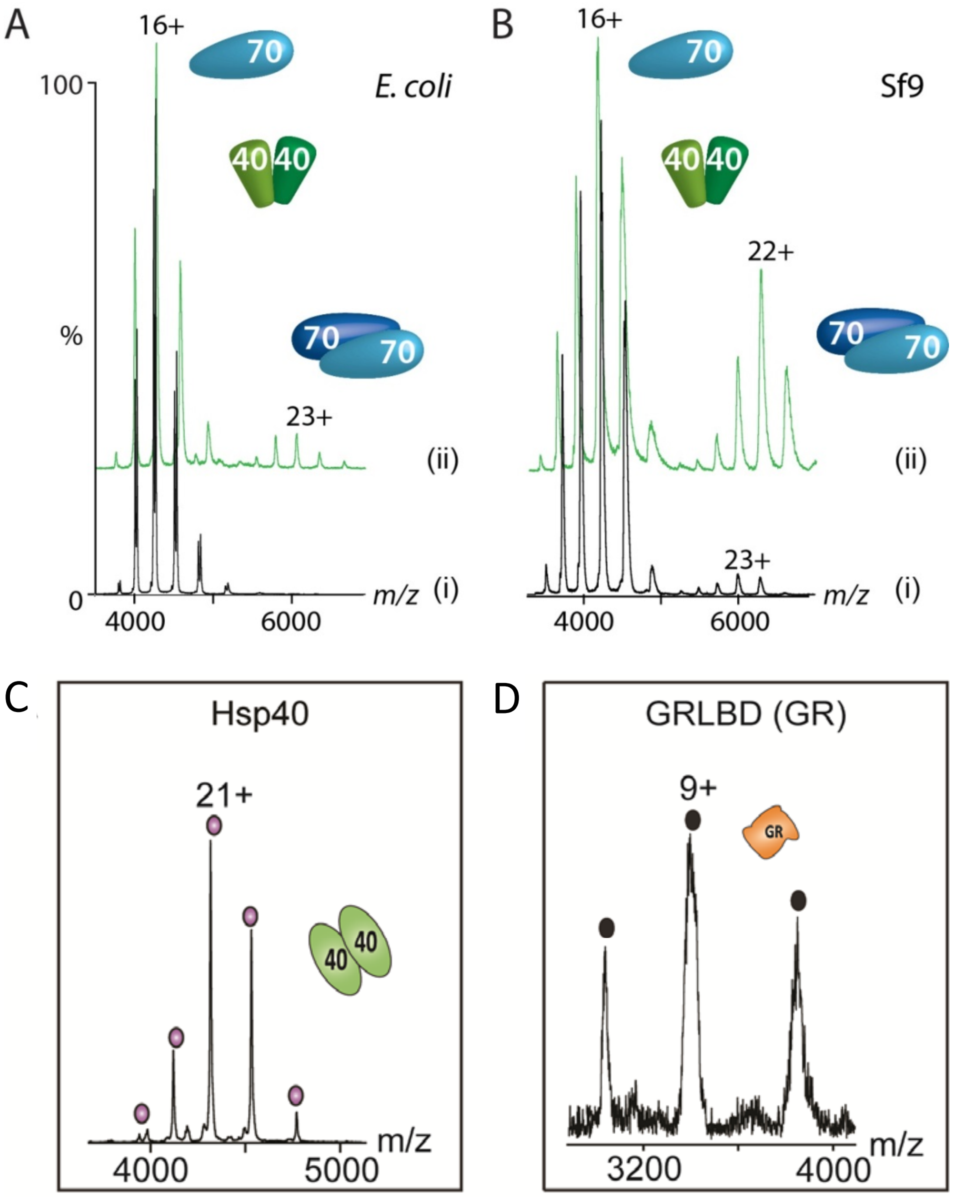

Figure S5 (related to Figure 2): Dimerization of Hsp70$_{E.\ coli}$ and Hsp70$_{Sf9}$ in the presence of Hsp40 and oligomeric states of Hsp40 and GR. (A) and **(B):** (i) Without Hsp40, (ii) in the presence of catalytic amounts of Hsp40. **(A)** Hsp70$_{E.coli}$ dimer is observed*. **(B)** Hsp70$_{Sf9}$ dimer intensity is increased. **(C)** and **(D):** The oligomeric states of Hsp40 and GRLBD were studied using mass spectrometry. Hsp40 was found to be predominantly dimeric under these conditions **(C)** while GRLBD is monomeric **(D)**.

*Hsp70$_{E.\ coli}$ contains His-tag

Supplemental Tables

Table S1 (related to Figure 1): Acetylation sites identified in ($Hsp70_{Sf9}$). Seven acetylation sites were identified by LC-MS/MS. Two of these sites were reported previously (Choudhary et al., 2009, Yang et al., 2013). The site, the peptide sequence, the number of spectra observed and the highest Mascot score for each acetylation site are given.

Acetylation site	Peptide sequence	# Spectra	Highest Mascot score	Identified previously
108	VQVSYK*GETK	8	44.57	Yes[1]
159	QATK*DAGVIAGLNVLR	7	89.72	Yes[2]
451	AMTK*DNNLLGR	40	58.48	No
507	ITITNDK*GR	4	40.27	No
512	LSK*EEIER	46	39.74	No
559	SAVEDEGLK*GK	3	60.40	No
561	GK*ISEADKK	4	36.74	No

Table S2 (related to Figures 1, 2, 3 and 4): Chemical cross-linking of Hsp70/Hsp90 complexes and subcomplexes. For every experiment in this study the protein concentration and volume, concentration and volume of the cross-linker (BS3), the number of cross-linked spectra, the number of unique cross-links and the FDR are listed. The peptide sequences of cross-linked di-peptides, the respective protein names and amino acid residues as well as the number of observed spectra and the maximum MassMatrix (MM) peptide score are given.

Table S3 (related to Figures 1, 2, 4 and 5). Masses of protein subunits and protein complexes observed in this study. Theoretical (expected) and experimentally measured masses of proteins and protein complexes are given in Da.

**Protein subunits**	**Expected mass (Da)**	**Measured mass (Da)**
GRLBD	30281	30291 ± 15
(Hsp40)$_2$	90332	90622 ± 13
(Hsp90)$_1$	85443	85521 ± 86
(Hsp90)$_2$	170887	170960 ± 70
Hsp70$_{E. coli}$* Hsp70$_{Sf9}$ Hsp70$_{E. coli}$ ^{13}C-labeled** Hsp70$_{E. coli}$ ^{15}N-labeled Hsp70$_{T504E}$ Hsp70$_{T504E\ V438F}$ ^{15}N-labeled	72231 70009 73774 71500 70667 71576	72656 ± 15 70922 ± 35 73843 ± 6 72170 ± 19 71256 ± 17 72250 ± 4
**Binary complexes**	**Expected mass (Da)**	**Measured mass (Da)**
(Hsp70$_{E. coli}$)$_2$* (Hsp70$_{Sf9}$)$_2$ (Hsp70$_{E. coli}$ ^{13}C-labeled)$_2$ (Hsp70$_{E. coli}$ ^{15}N-labeled)$_2$ (Hsp70$_{T504E}$)$_2$ (Hsp70$_{T504E\ V438F}$ ^{15}N-labeled)$_2$ (Hsp70$_{Sf9}$)(Hsp70$_{E. coli}$ ^{13}C-labeled) (Hsp70$_{T504E}$)(Hsp70$_{T504E\ V438F}$ ^{15}N-labeled) (Hsp70$_{T504E}$)(Hsp70$_{E. coli}$ ^{15}N-labeled)	144462 140018 147548 142999 141334 143151 143783 142243 142167	145435 ± 33 141822 ± 20 148844 ± 35 144447 ± 35 142724 ± 24 144517 ± 14 145010 ± 23 143611 ± 13 143539 ± 43
(Hsp90)$_2$(Hop)$_1$	233625	233825 ± 55
**Ternary complexes**	**Expected mass (Da)**	**Measured mass (Da)**
(Hsp90)$_2$(Hop)$_1$(Hsp70$_{E. coli}$)$_1$* (Hsp90)$_2$(Hop)$_1$(Hsp70$_{Sf9}$)$_1$ (Hsp90)$_2$(Hop)$_1$(Hsp70$_{Sf9}$)$_2$	305856 304253 374891	306587 ± 70 305253 ± 40 375422 ± 33
(Hsp90)$_2$(Hop)$_2$(Hsp70$_{E. coli}$)$_1$*	368594	370065 ± 61
**GR complexes**	**Expected mass (Da)**	**Measured mass (Da)**
(Hsp70$_{E. coli}$)$_1$(GRLBD)$_1$*	102512	103004 ± 20
(Hsp40)$_2$(GRLBD)$_1$	120613	121084 ± 19
(Hsp90)$_2$(Hop)$_1$(GRLBD)$_1$(Hsp70$_{E. coli}$)$_1$*	336137	337294 ± 62
(Hsp90)$_2$(Hop)$_1$(GRLBD)$_1$(Hsp70$_{E. coli}$)$_2$*	408368	410063 ± 89
(Hsp90)$_2$(Hop)$_1$(GRLBD)$_1$(Hsp70$_{Sf9}$)$_2$	405172	405963 ± 50

*Hsp70$_{E. coli}$ contains His-tag

**Hsp70$_{E. coli}$ nucleotide free-form

Table S4 (related to 'Methods'): Template PDB IDs used to generate homology models and PDB IDs of used high-resolution structures.

**Protein**	**Template PDB ID (organism)**	**Sequence identity with template**
Hsp90	2CG9 (*S. cerevisiae*)	58 %
Hsp70 (ATP state)	4B9Q (*E. coli*)	48 %
Hop (TPR2A and B)	3UQ3 (*S. cerevisiae*)	45 %
**Protein**	**PDB ID (organism)**	
Hsp70 (ADP state)	2KHO (*E. coli*)	
Hsp40	1NLT (*S. cerevisiae*)	
Hsp40 dimerization domain	1XAO (*S. cerevisiae*)	
J-domain of Hsp 40	2O37 (*S. cerevisiae*)	
GRLBD	3E7C (*H. sapiens*)	

Supplemental Methods

MS instrument parameters for intact protein complexes

Individual proteins: capillary voltage 1.7 kV, cone voltage 60 V, extractor 5 V, collision voltage 40 V, backing pressure 3.8 x 10^{-3} - 6.2 x 10^{-3} mbar.

Hsp70-Hsp40-GR complexes: capillary voltage 1.8 kV, cone voltage 100 V, extractor 5 V, collision voltage 80V, backing pressure 1.2 x 10^{-2} mbar.

Hsp90 complexes with Hop, Hsp70 and GR: capillary voltage 1.7-1.8 kV, cone voltage 100 V, extractor 5 V, collision voltage 100V, backing pressure 9.65 x 10^{-3} mbar.

For MSMS experiments, collision voltages varied up to 200 V.

Phosphopeptide enrichment

Phosphopeptides were enriched using titanium dioxide (TiO_2). Enrichment columns were packed into pipette tips using TiO_2 material (GL Sciences). The columns were washed with 5 % TFA/80 % ACN and reconstituted in 20 % 2,5-dihydroxybenzoic acid (DHB)/5 % TFA/80 % ACN. Peptides were dissolved in 20 % DHB/5 % TFA/80 % ACN and loaded onto the material. After washing with 20 % DHB/5 % TFA/80 % ACN and 5 % TFA/80 % ACN, phosphopeptides were eluted with 0.3 N ammonia solution (pH > 10.5). Eluted peptides were dried in a vacuum centrifuge for LC-MS/MS analysis.

Chemical XL

Protein complexes were cross-linked with BS3 in binding buffer. The protein and cross-linker concentrations are stated in Table S2. XL reactions were incubated for 1 hr at 25°C and 450 rpm in a thermomixer. Proteins were separated by SDS-PAGE and digested with trypsin in-gel as described (Shevchenko et al., 1996) or were precipitated with ethanol and digested in-solution using RapiGest SF Surfactant (Waters) according to manufacturer's protocols. Peptides obtained from in-solution digestion were re-dissolved in 20 % ACN, 4 % FA and further separated by cation exchange chromatography using SCX stage tips (Thermo Scientific) according to the manufacturer's protocol. Peptides were eluted with different concentrations of ammonium acetate (50 mM, 100 mM, 200 mM and 500 mM) and dried in a vacuum centrifuge. The mixture of cross-linked and non-cross-linked peptides was analysed by LC-MS/MS.

LC-MS/MS for identification of cross-linked peptides and phosphosites

Tryptic peptides were separated by nano-flow reversed-phase liquid chromatography (DionexUltiMate 3000 RSLC nano System, Thermo Scientific; mobile phase A, 0.1 % (v/v) formic acid (FA); mobile phase B, 80 % (v/v) ACN/0.1 % (v/v) FA) coupled to an LTQ-Orbitrap XL mass spectrometer (Thermo Scientific). Peptides were loaded onto a trap column (HPLC column Acclaim® PepMap100, C18, 100 µm I.D. particle size 5µm; Thermo scientific) and separated with a flow rate of 300 nL/min on an analytical C18 capillary column (50 cm, HPLC column Acclaim® PepMap100, C18, 75 µm I.D. particle size 3 µm; Thermo Scientific), with a gradient of 5-80 % (v/v) mobile phase B over 74 min. Peptides were directly eluted into the mass spectrometer.

MS conditions were: spray voltage of 1.8 kV; capillary temperature of 180 °C; normalized collision energy of 35% at an activation of q = 0.25 and an activation time of 30 ms. The LTQ-Orbitrap XL was operated in data-dependent mode. Survey full scan MS spectra were acquired in the Orbitrap (*m*/*z* 300−2000) with a resolution of 30,000 at *m*/*z* 400 and an automatic gain control (AGC) target at 10^6. The five most intense ions were selected for CID in the linear ion trap at an AGC target of 30,000. For identification of phosphopeptides, multistage activation was enabled for neutral loss masses of one, two, three and four phosphosites per doubly and triply charged peptide. Detection in the linear ion trap of previously selected ions was excluded for 30 s. Singly charged ions and ions with unrecognized charge state were excluded. Internal calibration of the Orbitrap was performed using the lock mass option (lock mass: *m*/*z* 445.120025 (Olsen et al., 2005).

LC-MS/MS for identification of acetylation sites

Proteins were separated by SDS-PAGE and digested as described (Shevchenko et al., 1996). Peptides were separated by nano-flow reversed-phase liquid chromatography (EASY nLC 1000, Thermo Scientific; mobile phase A, 0.1 % (v/v) formic acid (FA)/5 % (v/v) DMSO; mobile phase B, 100 % (v/v) ACN/0.1 % (v/v) FA/5 % (v/v) DMSO) coupled to a Q Exactive Orbitrap mass spectrometer (Thermo Scientific). Peptides were loaded onto a trap column (5 mm, PepMap RSLC, C18, 300 µm I.D. particle size 3 µm; Thermo Scientific) and separated with a flow rate of 200 nL/min on an analytical C18 capillary column (50 cm, PepMap RSLC, EASY-spray column, C18, 75 µm I.D. particle size 3 µm; Thermo Scientific), with a gradient of 7-30 % (v/v) mobile phase B over 30 min. Peptides were directly eluted into the mass spectrometer.

MS conditions were: spray voltage of 2.1 kV; capillary temperature of 320 °C. The Q Exactive Orbitrap was operated in data-dependent mode. Survey full scan MS spectra were acquired in the orbitrap (*m*/*z* 350−1500) with a resolution of 70,000 an AGC target at 3×10^6. The ten most intense ions were selected for HCD at an AGC target of 50,000.

Database search

Identification of cross-links. Potential cross-links were identified using the MassMatrix Database Search Engine (Xu et al., 2010). Search parameters were: Tryptic peptides with a maximum of two missed cleavage sites. Carbamidomethylation of cysteine and oxidation of methionine as variable modifications. Mass accuracy filter: 10 ppm for precursor ions, 0.8 Da for fragment ions. Minimum pp and pp2 values 5.0, minimum pptag 1.3. Maximum number of cross-links per peptide was 1. All searches were performed twice including deuterated (d4) and non-deuterated (d0) BS3, respectively. Cross-links were validated by (i) the presence of the peak pair in the MS spectra generated by the d4/d0-BS3-mixture, and (ii) by the quality of the MS/MS spectrum. For comparative cross-linking, extracted ion chromatograms (XICs) for the light and heavy cross-links, respectively, were generated. The two states of Hsp70 were compared by the area of the XICs.

Identification of phosphosites. Raw data were searched against NCBI non-redundant database with *Homo sapiens* taxonomy filter (248,775 sequences) using Mascot v2.4.1 search engine (Matrix Science). Mass accuracy filter: 15 ppm for precursor ions, 0.5 Da for MS/MS fragment ions. Tryptic peptides with maximal two missed cleavage sites. Carbamidomethylation of cysteine and oxidation of methionine as well as phosphorylation of serine, threonine and tyrosine as variable modifications.

Identification of acetylation sites. Raw data were searched against SwissProt database with *Homo sapiens* taxonomy filter (20,353 sequences) using the Mascot v2.4.1 search engine. Mass accuracy filter: 7 ppm for precursor ions, 0.005 Da for MS/MS fragment ions. Tryptic peptides with maximal two missed cleavage sites. Carbamidomethylation of cysteine and oxidation of methionine as well as acetylation of lysine and protein N-termini as variable modifications.

References

CHOUDHARY, C., KUMAR, C., GNAD, F., NIELSEN, M. L., REHMAN, M., WALTHER, T. C., OLSEN, J. V. & MANN, M. 2009. Lysine acetylation targets protein complexes and co-regulates major cellular functions. *Science,* 325**,** 834-40.

OLSEN, J. V., DE GODOY, L. M., LI, G., MACEK, B., MORTENSEN, P., PESCH, R., MAKAROV, A., LANGE, O., HORNING, S. & MANN, M. 2005. Parts per million mass accuracy on an Orbitrap mass spectrometer via lock mass injection into a C-trap. *Mol Cell Proteomics,* 4**,** 2010-21.

SHEVCHENKO, A., WILM, M., VORM, O. & MANN, M. 1996. Mass spectrometric sequencing of proteins silver-stained polyacrylamide gels. *Anal Chem,* 68**,** 850-8.

WU, C. C., NAVEEN, V., CHIEN, C. H., CHANG, Y. W. & HSIAO, C. D. 2012. Crystal structure of DnaK protein complexed with nucleotide exchange factor GrpE in DnaK chaperone system: insight into intermolecular communication. *J Biol Chem,* 287**,** 21461-70.

XU, H., HSU, P. H., ZHANG, L., TSAI, M. D. & FREITAS, M. A. 2010. Database search algorithm for identification of intact cross-links in proteins and peptides using tandem mass spectrometry. *J Proteome Res,* 9**,** 3384-93.

YANG, Y., FISKUS, W., YONG, B., ATADJA, P., TAKAHASHI, Y., PANDITA, T. K., WANG, H. G. & BHALLA, K. N. 2013. Acetylated hsp70 and KAP1-mediated Vps34 SUMOylation is required for autophagosome creation in autophagy. *Proc Natl Acad Sci U S A,* 110**,** 6841-6.
