# Supplementary material for: Hsp70 Forms Antiparallel Dimers Stabilized by Post-translational Modifications to Position Clients for Transfer to Hsp90
Source: Cell Rep. 2015 Apr 23;11(5):759–69. doi: 10.1016/j.celrep.2015.03.063 (PMC4431665; doi:10.1016/j.celrep.2015.03.063)
Supplement: Document S2. Article plus Supplemental Information [file mmc3.pdf]

Cell Reports

Article

Hsp70 Forms Antiparallel Dimers Stabilized by Post-translational Modifications to Position Clients for Transfer to Hsp90

Graphical Abstract

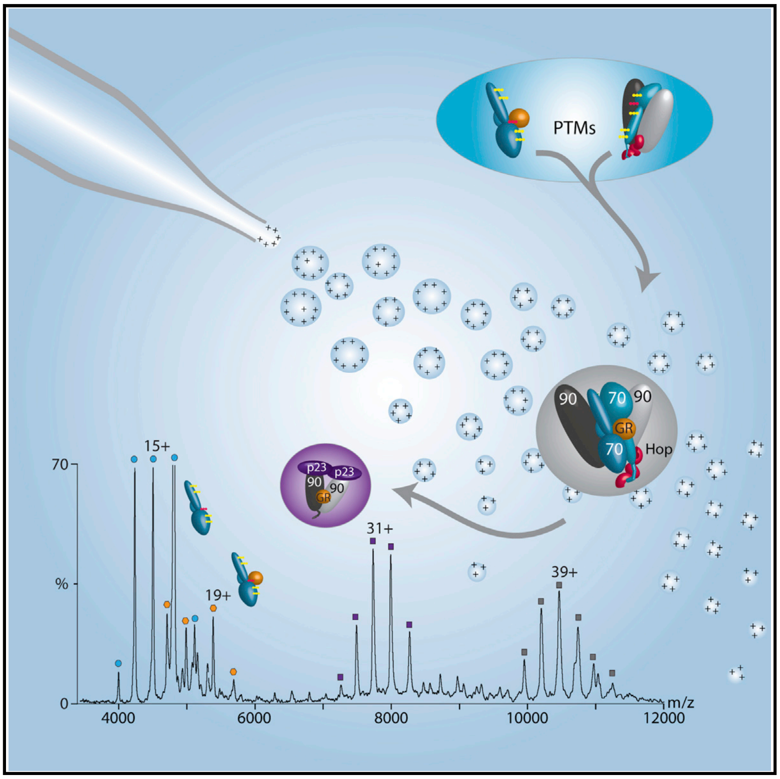

Authors

Nina Morgner, Carla Schmidt, ...,
David Agard, Carol V. Robinson

Correspondence

morgner@chemie.uni-frankfurt.de (N.M.),
carol.robinson@chem.ox.ac.uk (C.V.R.)

In Brief

Morgner et al. combine native mass spectrometry and chemical crosslinking to define the interactions of the Hsp70/90 chaperone system. They show that Hsp70 dimerization is antiparallel and stabilized by PTMs. They monitor the formation of chaperone complexes and discover a hexameric client-loading complex containing an Hsp70 dimer.

Highlights

- Antiparallel dimerization of Hsp70 is stabilized by PTMs
- Hsp40 catalyzes Hsp70 dimerization and client transfer to Hsp70
- Hsp70 antiparallel dimerization is maintained in the client-loading complex
- Addition of p23 induces transfer of GR onto Hsp90 and loss of Hop and Hsp70

Morgner et al., 2015, Cell Reports *11*, 759–769
May 5, 2015 ©2015 The Authors
http://dx.doi.org/10.1016/j.celrep.2015.03.063

OPEN ACCESS
CellPress

Hsp70 Forms Antiparallel Dimers Stabilized by Post-translational Modifications to Position Clients for Transfer to Hsp90

Nina Morgner,[1,5,*] Carla Schmidt,[1,5] Victoria Beilsten-Edmands,[1,5] Ima-obong Ebong,[1] Nisha A. Patel,[1] Eugenia M. Clerico,[2] Elaine Kirschke,[3] Soumya Daturpalli,[4] Sophie E. Jackson,[4] David Agard,[3] and Carol V. Robinson[1,*]
[1]Department of Chemistry, University of Oxford, South Parks Road, Oxford OX1 3QZ, UK
[2]Department of Biochemistry and Molecular Biology, University of Massachusetts, Amherst, MA 01003, USA
[3]Department of Biochemistry and Biophysics, Howard Hughes Medical Institute, University of California, San Francisco, San Francisco, CA 94158, USA
[4]Department of Chemistry, University of Cambridge, Lensfield Road, Cambridge CB2 1EW, UK
[5]Co-first author
*Correspondence: morgner@chemie.uni-frankfurt.de (N.M.), carol.robinson@chem.ox.ac.uk (C.V.R.)
http://dx.doi.org/10.1016/j.celrep.2015.03.063
This is an open access article under the CC BY license (http://creativecommons.org/licenses/by/4.0/).

SUMMARY

Protein folding in cells is regulated by networks of chaperones, including the heat shock protein 70 (Hsp70) system, which consists of the Hsp40 cochaperone and a nucleotide exchange factor. Hsp40 mediates complex formation between Hsp70 and client proteins prior to interaction with Hsp90. We used mass spectrometry (MS) to monitor assemblies formed between eukaryotic Hsp90/Hsp70/Hsp40, Hop, p23, and a client protein, a fragment of the glucocorticoid receptor (GR). We found that Hsp40 promotes interactions between the client and Hsp70, and facilitates dimerization of monomeric Hsp70. This dimerization is antiparallel, stabilized by post-translational modifications (PTMs), and maintained in the stable heterohexameric client-loading complex Hsp90$_2$Hsp70$_2$HopGR identified here. Addition of p23 to this client-loading complex induces transfer of GR onto Hsp90 and leads to expulsion of Hop and Hsp70. Based on these results, we propose that Hsp70 antiparallel dimerization, stabilized by PTMs, positions the client for transfer from Hsp70 to Hsp90.

INTRODUCTION

Hsp70 and Hsp90 are essential and abundant molecular chaperones in the eukaryotic cytosol and are involved in the folding and maturation of a myriad of protein substrates, including many cancer-causing proteins (Brychzy et al., 2003, Taipale et al., 2010). The Hsp70/90 system requires a cohort of cochaperones to provide specificity and regulation of the chaperone interactions with their client proteins (Li et al., 2011, Picard et al., 1990, Young et al., 2001). Hsp70 binds extended hydrophobic peptide sequences and acts at an early stage to recognize partially folded client proteins. Hsp90 is thought to interact with near-native conformations of its substrates, and its clients include protein kinases and steroid hormone receptors, the latter being the most extensively studied (Jackson, 2013).

Hsp90 forms defined binary or ternary complexes with cochaperones to facilitate the maturation of client proteins (reviewed in Prodromou, 2012). Structurally, it consists of a C-terminal dimerization region, a middle domain, and an N-terminal nucleotide-binding domain (NBD) connected by a charged linker that provides the necessary flexibility for domain rearrangements (Tsutsumi et al., 2012). Hsp90 binds at its C-terminal MEEVD sequence to tetratricopeptide repeat (TPR) cochaperones, including the "Hsc70/Hsp90 organizing protein" Hop (Young et al., 1998).

In contrast to Hsp90, Hsp70 is predominantly monomeric, and high-resolution structures of the full-length eukaryotic protein have remained elusive. By analogy to the different ADP-bound states of DnaK, the *E. coli* Hsp70 homolog, the picture that is emerging shows independent movements of the N-terminal NBD and C-terminal substrate-binding domain (SBD) (Bertelsen et al., 2009, Swain et al., 2007). These movements are thought to be lost upon ATP binding when Hsp70 rearranges into a domain-docked structure (Mapa et al., 2010). Together with a previous NMR study (Zhuravleva et al., 2012), these structures define the allosteric control mechanism between the NBD and SBD.

In previous studies, we established that Hsp70 was essentially monomeric under our solution conditions (Ebong et al., 2011), although dimerization has been reported previously in solution and X-ray structures of DnaK (Qi et al., 2013). Recently, specific mutations of DnaK were designed to disrupt the dimer interface observed crystallographically and to probe its functional significance (Sarbeng et al., 2015). Results showed that mutations on the dimer interface compromise both chaperone activity and Hsp40 interactions.

It is established that the Hsp40 cochaperones regulate ATP-dependent substrate binding of Hsp70 (Laufen et al., 1999, Mayer and Bukau, 2005) via interaction of the N-terminal J domains of Hsp40 with an acidic groove located in the NBD of

CrossMark

Hsp70 (Jiang et al., 2007). The C-terminal domain of Hsp40 from yeast contains an EEVD peptide-binding site (Li et al., 2006) and the dimerization domain (Li et al., 2003). Hsp40s are typically expressed at lower levels than Hsp70, consistent with their catalytic function and crucial role in Hsp70 function (Young, 2010). In addition, numerous reports have proposed an Hsp40-induced oligomerization of human Hsp70 or DnaK (Benaroudj et al., 1995, Cyr and Douglas, 1994, Hernández et al., 2002, Thompson et al., 2012). Participation of the Hsp70/Hsp40 chaperone system is also required to regulate client binding to Hsp90 (Pratt and Toft, 1997) and to load Hsp90 with a client protein (Hernández et al., 2002). Hop bridges the Hsp70 and Hsp90 chaperone systems (Chen and Smith, 1998), and also inhibits the ATPase activity of Hsp90, stabilizing the client-loading conformation and facilitating the handover of clients (Richter et al., 2003, Southworth and Agard, 2011).

For this study, we selected the transcription factor GR as the client protein because it is well characterized, particularly with respect to its associations with the Hsp90/Hsp70 systems (Sanchez, 2012). GR has to bind to Hsp90 as a prerequisite to attain a high-affinity ligand-binding conformation prior to its import into the nucleus (Dittmar et al., 1997, Picard et al., 1990, Smith, 1993). Here, we used a structure of its ligand-binding domain (LBD) in complex with the agonist dexamethasone (Bledsoe et al., 2002). During the course of this work, two cryo-electron microscopy (cryo-EM) studies (Alvira et al., 2014, Kirschke et al., 2014) revealed the formation of an Hsp90/Hsp70/Hop/GR complex proposing unfolding/inactivation of GR by Hsp70 and refolding/reactivation of GR by Hsp90, and revealing the location of GR with respect to Hop, which is located on the opposite side of the Hsp90. These complexes provide new insight into the location of GR, but also raise the question of whether they are primed for transfer of GR from Hsp70 to Hsp90.

In common with many chaperone systems, Hsp70/90-client interactions have proven challenging to study with traditional biophysical techniques due to their dynamic nature and compositional heterogeneity. For this reason, we applied mass spectrometry (MS) to probe the composition of stable complexes formed on these reaction pathways. The use of MS to study protein complexes is gaining momentum (Heck, 2008, Hilton and Benesch, 2012, Wyttenbach and Bowers, 2007). Pertinent to such studies is the ability of MS to determine the masses and hence the subunit stoichiometry of multi-protein complexes that form simultaneously within dynamic and heterogeneous assemblies (Benesch et al., 2006, Stengel et al., 2010). When coupled with chemical crosslinking (XL) strategies (Schmidt and Robinson, 2014), this approach is particularly powerful because it allows not only the stoichiometry but also the interaction interfaces to be defined (Schmidt et al., 2013).

By incubating subsets of proteins involved in the assembly of Hsp90/Hsp70/Hop/GR complexes, and by varying the order of addition and concentrations of the proteins and the level of nucleotides, we allow the Hsp90/Hsp70/Hsp40/GR complexes to assemble in solution. We then define their composition and interactions by recording mass spectra of the intact complexes. In addition, by employing chemical XL, we identify the interfaces between the complex components. Starting with Hsp70, we explore the extent of its oligomerization in solution with respect to its post-translational modification (PTM) status, and employ comparative chemical XL to compare dimeric interfaces in ATP- and ADP-bound states. Increasing the complexity to the Hsp70/Hsp40/GR system, we show that we can detect stable Hsp70-client complexes in the presence of catalytic quantities of Hsp40. We find that the predominant client-loading complex formed with Hsp90 and Hop contains dimeric Hsp70 and Hsp90, as well as one Hop and one GR. Chemical XL locates GR in close proximity to Hsp90 and enables us to identify roles for Hsp40 and Hsp70 in transferring clients to the Hsp90 cycle, and to propose a role for Hsp70 dimerization in this client-loading complex.

RESULTS

PTMs Stabilize Hsp70 Dimers

To investigate interactions in Hsp70 and to probe the existence of higher oligomeric states proposed previously (Aprile et al., 2013, King et al., 1999), we examined by MS the propensity of Hsp70 to form oligomers. We expressed and purified human Hsp70 in two expression systems: Sf9 insect cells and *E. coli.* We then compared the peak intensities of the oligomers in the same spectrum using Hsp70 expressed in *E. coli* ($Hsp70_{E.\ coli}$) labeled with heavy isotopes (^{13}C), separating it by mass from Hsp70 expressed in Sf9 cells ($Hsp70_{Sf9}$). A 1:1 mixture of the two proteins at a high protein concentration (8 μM) under experimental parameters designed to preserve non-covalent interactions (Hernández and Robinson, 2007) reveals dimerization (Figure 1A). Interestingly the mass spectrum shows that for the $Hsp70_{Sf9}$:$Hsp70_{E.\ coli}$ mixture, the $Hsp70_{Sf9}$ dimer has higher intensity than its *E. coli*-expressed counterpart, implying that the $Hsp70_{Sf9}$ dimer is more stable than $Hsp70_{E.\ coli}$ (Figure 1B). Given that the amino acid sequences of $Hsp70_{Sf9}$ and $Hsp70_{E.\ coli}$ are identical, but expression in *E. coli* does not allow for PTMs, we hypothesized that this enhanced interaction was due to acetylation and phosphorylation, which often combine to stabilize subunit interfaces (van Noort et al., 2012). We first investigated the occurrence of acetylation in $Hsp70_{Sf9}$ and identified seven lysine acetylation sites, two of which had been reported previously (Table S1). We then applied a phosphopeptide enrichment strategy and found a phosphosite (T504) in $Hsp70_{Sf9}$, at a known phosphorylation hotspot (Beltrao et al., 2012). This site is not phosphorylated in $Hsp70_{E.\ coli}$ and, together with the multiple acetylation sites, provides a plausible rationale for the enhanced stability of the $Hsp70_{Sf9}$ dimer.

As this particular phosphosite is highly conserved in different eukaryotic species (Beltrao et al., 2012), we probed its significance for dimerization and incubated the $Hsp70_{Sf9}$ dimer with a phosphatase. We prepared two aliquots containing a 1:1 solution of $Hsp70_{Sf9}$ and ^{13}C -labeled $Hsp70_{E.\ coli}$ (one with buffer and one with phosphatase). Following overnight incubation without phosphatase, $Hsp70_{Sf9}$ retained a population of dimers (Figures 1B and 1C). Interestingly, a mixed heterodimer, $Hsp70_{Sf9}$:$Hsp70_{E.coli}$, formed under these incubation conditions, consistent with subunit exchange occurring within a 16 hr timescale. By contrast, no $Hsp70_{E.\ coli}$ homodimer was observed, supporting enhanced dimerization of $Hsp70_{Sf9}$. Peak splitting of monomeric Hsp70 (Figures 1A and 1C) was attributed to *apo* and nucleotide-bound forms of the proteins, implying loss of

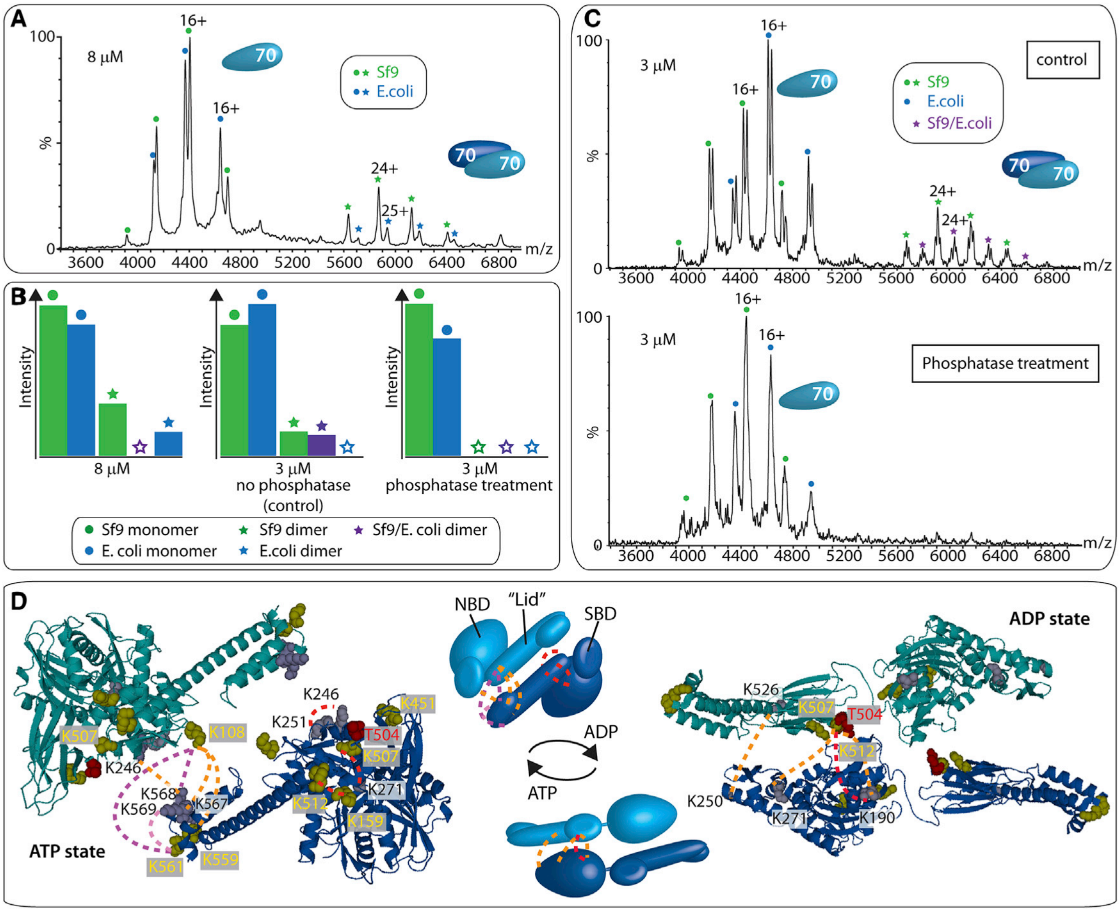

Figure 1. PTMs Promote Dimerization of Hsp70

(A) Mass spectrum of an 8 μM solution containing a 1:1 ratio of ^{13}C-labeled Hsp70$_{E.\ coli}$ and Hsp70$_{Sf9}$ with natural abundance isotopes. At these concentrations, both are predominantly monomeric, but a higher population of Hsp70 dimers is observed for the Sf9-expressed protein.

(B) Bar charts comparing the intensities of monomeric and dimeric Hsp70. Green, Sf9; blue, *E. coli*; purple, Sf9/*E. coli*. Monomers and dimers are labeled with circles and stars, respectively.

(C) Mass spectrum recorded at high backing pressure to promote dimer formation (Hernández and Robinson, 2007) of a 1:1 ratio of 3 μM ^{13}C- labeled Hsp70$_{E.\ coli}$ and Hsp70$_{Sf9}$ with natural abundance isotopes, incubated overnight in the absence of phosphatase ("control," top) or in the presence of phosphatase ("phosphatase treatment," bottom). Both spectra were acquired under the same conditions. Note the absence of dimer under the phosphatase condition.

(D) XLs are highlighted on structures of Hsp70 subunits in ATP and ADP states. The ADP state was manually aligned with the human Hsp70 sequence. Inter-subunit XLs are highlighted on antiparallel dimers of both states (shown schematically). In the ATP state, XLs K108-K561/569 (purple) orient two Hsp70 subunits in an antiparallel dimer. The phosphosite T504 in Hsp70$_{Sf9}$ (red) and the lysine-binding pockets are located both adjacent to and across the interface (gray and yellow). Comparative XL of the two Hsp70 proteins (expressed in *E. coli* and Sf9) shows that the K108-K569/561 is 5-fold more intense in Hsp70$_{Sf9}$ than in Hsp70$_{E.\ coli}$, consistent with the stronger interface in Hsp70$_{Sf9}$ (purple). Less dimerization for the *E. coli*-expressed Hsp70 is consistent with the observed 3-fold increase of the K569-561 intra-XL. Inter-subunit XLs (red) are consistent with the ATP state.

See also Figures S1–S4 and Tables S1, S2, and S3.

nucleotide and ATP turnover during the overnight incubation. The second aliquot, to which phosphatase was added, showed peak splitting due to the loss of nucleotide, but importantly, no dimers were observed (Figures 1B and 1C). To confirm that the Hsp70$_{E.\ coli}$ dimer was not affected by incubation with phosphatase, we repeated these experiments at a higher Hsp70$_{E.\ coli}$ concentration under which dimerization occurs. We found that the dimer was stable for 48 hr with phosphatase present (Figures S1A and S1B). We conclude that since the Hsp70$_{Sf9}$:Hsp70$_{E.\ coli}$ heterodimer and Hsp70$_{Sf9}$ homodimer are weakened by addition of phosphatase, the phosphorylation site in Hsp70$_{Sf9}$ not only stabilizes the homodimer but also promotes formation of the heterodimer.

Given the presence of PTMs and their potential role in stabilizing the dimer interface, we anticipated that the Hsp70$_{Sf9}$ dimer interface might be affected by changes in ionic interactions. Therefore, we recorded mass spectra for solutions containing 2 μM Hsp70$_{Sf9}$, 0.5 μM Hsp40, and 200 μM ATP with increasing ionic strength from 50 to 300 mM potassium chloride (Figure S2). The results show a decrease in the population of dimers at higher ionic strength, consistent with ionic interactions maintaining the dimer interface.

To confirm the proposed role of T504 in enhancing the dimer interface, we prepared a phosphomimic mutant, replacing T504 in Hsp70$_{E.\ coli}$ with a glutamic acid residue to form a T504E variant. A comparison of the mass spectra of the labeled wild-type Hsp70$_{E.\ coli}$ with those of the Hsp70$_{E.\ coli,T504E}$ variant shows an increase in the population of dimers for the phosphomimic when examined in a 1:1 ratio (Figure S1C). Together, these results show that the Hsp70$_{Sf9}$ dimer interface is strengthened by ionic interactions and that a key phosphosite, supported by multiple acetylation sites, contributes to its stability.

Chemical XL Defines an Antiparallel Hsp70 Dimer

We defined the interface of the Hsp70$_{Sf9}$ dimer by XL with bis(-sulfosuccinimidyl)suberate (BS3) first in the presence of excess ATP. SDS-PAGE analysis confirmed the presence of monomers and crosslinked dimers, showing that populations of dimers formed under these conditions (Figure S3A). Gel bands assigned to the monomer and dimer were then subjected to tryptic digestion and liquid chromatography-tandem MS (LC-MS/MS) analysis. We found 170 potential XLs by a database search. We manually validated 154 of these XLs, giving a false-discovery rate (FDR) of 9.61% (Table S2).

We considered a number of high-resolution structures for generation of homology models, including one in which the SBD binds to the hydrophobic linker (Chang et al., 2008) and the *Bos taurus* Hsc70 structure (Jiang et al., 2005). We also considered a model generated from *Geobacillus kaustophilus* (Chang et al., 2008, Wu et al., 2012). This model shows Hsp70 oligomerization via the SBD of one Hsp70 molecule binding to the flexible linker of a second Hsp70 (Figure S4A; Wu et al., 2012). To investigate this dimerization mechanism, we generated a substrate-binding-deficient variant of Hsp70$_{E.coli}$ (V438F), which also includes the phosphomimic T504E and was shown to be unable to bind substrates in DnaK and Hsp70 (Mayer et al., 2000, Rohrer et al., 2014). However, the V438F/T504E mutant was able to dimerize despite its substrate-binding deficiency, ruling out the substrate-binding model for dimerization (Figure S4B).

The two high-resolution structures that best accommodate our XLs are the ADP- and ATP-bound states of DnaK (PDB IDs 2KHO and 4B9Q, respectively (Bertelsen et al., 2009, Kityk et al., 2012). We generated a homology model for the ATP state using the structure 4B9Q as a template. For the ADP state, we could not obtain a good homology model, and therefore we manually aligned the human Hsp70 sequence with the solution structure of *E. coli* DnaK (Bertelsen et al., 2009; Figure 1D). Of the multiple intra-subunit XLs derived from the band assigned to the monomer, two were of interest: K190-K507 and K190-K512. Both of these XLs are accommodated better in the ADP state (Bertelsen et al., 2009) than in the ATP state (Kityk et al., 2012). We also observed two intra-XLs consistent with the ATP conformation, but not the ADP state: K159-K512 and K246-K271. The fact that both ATP and ADP conformations are satisfied by our XL restraints implies that ATP hydrolysis takes place, giving rise to the two nucleotide-bound forms that readily interconvert in solution.

Two of the 11 Hsp70 XLs derived from the dimer band, K569-K108 and K561-K108 (both identified with ADP, ATP, or ATPγS) were particularly intriguing. These XLs cannot be assigned to intra-subunit XLs due to the distances required to link the NBD with the lid of the SBD. These crosslinked regions define a dimer interface that can be accommodated in either the ATP or ADP state, and clearly define an antiparallel orientation via interactions between the NBD and SBD of different subunits.

Given that XL provides an average ensemble populated in solution, we need a direct readout of the differences between the two distinct nucleotide-bound conformations of Hsp70 in the dimer. Therefore, using a previously described comparative XL strategy (Schmidt and Robinson, 2014, Schmidt et al., 2013), we added BS3-d$_0$ and BS3-d$_4$ to ADP- and ATP-containing solutions of Hsp70, respectively. After XL, a 1:1 molar ratio of the two solutions was digested with trypsin prior to LC-MS/MS analysis. This allowed for a quantitative comparison of XLs under different nucleotide conditions (Table S2). We found that in the presence of ADP, the XL K190-K507 was enhanced, defining close interactions in the ADP state. In excess ATP, however, K159-K512 and K246-K271/251 were significantly increased relative to XLs observed in the presence of ADP. These three XLs can only be accommodated in the ATP state. Since it was not possible to obtain XLs exclusive to one conformation (docked or undocked) in the presence of excess ATP, ADP, or ATPγS, and given the likely hydrolysis of ATP, we conclude that the dimer exists in a dynamic equilibrium perturbed by nucleotides but without a single defined conformer.

If we place the PTMs defined above within the context of the antiparallel dimer, then we find that the phosphosite (T504) is close to the hinge region between the SBD β-domain and the SBD α-helical lid, in a lysine-rich pocket that orients it toward the subunit interface for interactions with multiple lysine residues (K561, K567, K568, and K569) in the ATP state (Figure 1D). This network of lysine interactions in the ADP state, close to the phosphosite, could also stabilize the ADP conformation in the antiparallel dimer. The seven acetylation sites identified here also align along the dimer interface in the ATP state, implying that they combine with the phosphosite for signal propagation. These hydrogen-bonding interactions provide a rationale for increased dimerization of Hsp70$_{Sf9}$ and the conservation of the phosphosite (Beltrao et al., 2012), and the correspondence of the amino acid residues involved in acetylation (http://www.uniprot.org) in eukaryotes suggests their functional relevance in vivo.

Transient Interactions with Hsp40 Promote Hsp70 Dimerization

We investigated the effects of Hsp40 on the extent of dimer formation in both Hsp70$_{E.\ coli}$ and Hsp70$_{Sf9}$ using a 1:1 ratio of the two Hsp70s and catalytic amounts of Hsp40 (Figure 2A). In the presence of Hsp40, an increase in the population of the non-covalent dimer was observed for Hsp70$_{Sf9}$ and even for Hsp70$_{E.coli}$, albeit at a lower intensity (Figures S5A and S5B). An Hsp70$_{E.\ coli}$:Hsp70$_{Sf9}$ heterodimer was observed as above, consistent with the greater propensity of Hsp70$_{Sf9}$ (compared with Hsp70$_{E.\ coli}$) to form dimers.

To probe potential differences in the dimer interfaces of Hsp70$_{Sf9}$ and Hsp70$_{E.\ coli}$, we employed the comparative XL strategy described above in the presence of excess ATP and co-chaperone Hsp40 to enhance dimer formation. To avoid subunit exchange and to compare directly the strengths of the different

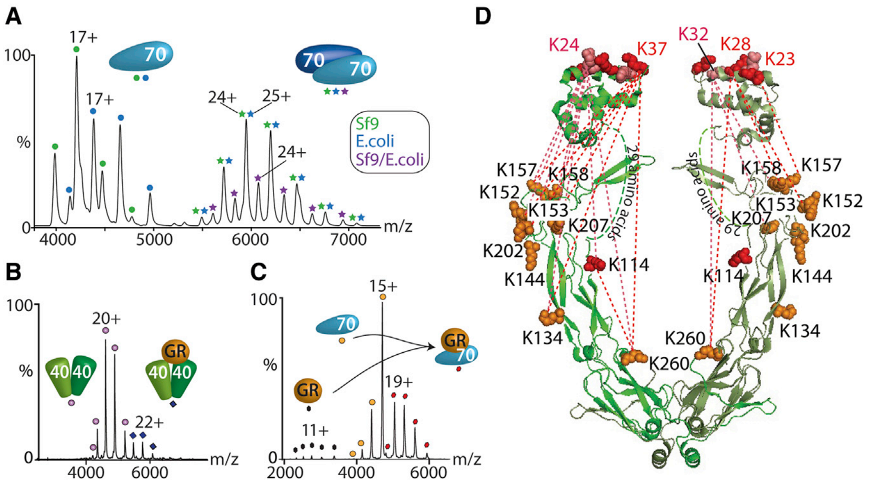

Figure 2. Hsp40 Promotes Interactions with Hsp70 and GR through Its J Domain

(A) Mass spectrum of a 1:1 ratio of ^{13}C-labeled $Hsp70_{E.\ coli}$ and $Hsp70_{Sf9}$ with natural abundance isotopes in the presence of Hsp40 and ATP. An increase in the population of the $Hsp70_{Sf9}$ dimer and formation of a heterodimer are observed.

(B and C) The mass spectrum of GR in the presence of Hsp40 shows only a low population of $Hsp40_2$GR (B), but a larger population of $Hsp70_{E.\ coli}$ GR is formed when catalytic amounts of Hsp40 are added (C). $Hsp70_{E.\ coli}$ contains a His-tag.

(D) Chemical XL highlights the dynamics of the Hsp40 dimer. Lysine residues in the region of 23–37 in the J domains make multiple interactions with the C-terminal and middle domains of Hsp40. For this symmetrical dimer, we expect XLs to be present on both subunits. For clarity, the two subunits are shown with different XLs.

See also Figure S5 and Table S2.

interfaces, we employed BS3-d_0 and $Hsp70_{Sf9}$ for $Hsp70_{E.\ coli}$ and BS3-d_4, respectively, and crosslinked them individually. Equal aliquots of the crosslinked proteins were pooled, digested, and analyzed by LC-MS/MS. We identified 466 XLs after a database search and validated 392 of these manually, giving an FDR of 15.9% (Table S2). Rejecting XLs with peptides of three or fewer amino acids results in 74 XLs (59 Hsp40-Hsp40, 12 Hsp70-Hsp70, and three Hsp40-Hsp70). Differences in subunit interactions can then be related to the extent of dimer formation by changes in the intensity ratio of the inter-protein XLs.

When we compared the intensities of the light and heavy crosslinked peptides, we found that two were noticeably different for the $Hsp70_{Sf9}$ and $Hsp70_{E.\ coli}$ dimers. The K569-K108 (assigned to the inter-subunit XL above) intensity ratio was 5.3:1.0 for the $Hsp70_{Sf9}$ and $Hsp70_{E.\ coli}$ peptides, respectively. This represents a 5-fold increase in the intensity of the crosslinked $Hsp70_{Sf9}$ dimer and is assigned to enhanced interactions in the $Hsp70_{Sf9}$ dimer interface. Interestingly, we identified a second XL, also involving K569 but this time crosslinked to K561, assigned to an intra-XL due to its close proximity. This XL showed a difference in the intensity ratio in the opposite direction, with an increase in $Hsp70_{E.\ coli}$:$Hsp70_{Sf9}$ to 3.4:1.0. Therefore, we conclude that a significant increase in intensity in the XL K569-K561 results from reduced dimer formation in $Hsp70_{E.\ coli}$, thereby promoting intra-molecular XL.

Hsp70 dimerization is significantly enhanced in the presence of sub-stoichiometric quantities of Hsp40, and yet no Hsp40-containing complexes were observed with low Hsp40 concentrations (Figure 2A). This supports the current view of a transient catalytic interaction (Kampinga and Craig, 2010). To address how this transient interaction with Hsp40 enhances dimerization, we first determined the oligomeric state of Hsp40 and observed a dimeric state (Figure S5C; Table S3). Given that Hsp70 and Hsp40 are known to promote client interactions (Kampinga and Craig, 2010), we reasoned that it might be necessary to include a client to stabilize the interactions between them. Using apo GR or apo MBP-GR (GR-fused maltose-binding protein to enhance solubility), both monomeric (Figure S5D), we probed client interactions with Hsp40. Interestingly Hsp40 with GR showed only minimal binding in a 1:1 stoichiometry, independent of the presence of nucleotides (Figure 2B). Incubating Hsp70 with GR in the presence of catalytic amounts Hsp40 and ATP revealed the formation of an Hsp70GR complex (Figure 2C). Significantly, Hsp40 was not incorporated into the Hsp70 complex, even in the presence of the client.

Dynamic Interactions between Hsp70 and Hsp40

Next, we probed interactions within and between Hsp40 and $Hsp70_{E.\ coli}$ using XL in the presence of GR and nucleotides. To eliminate stabilization by PTMs, we employed $Hsp70_{E.\ coli}$. We identified 57 intra-XLs, 42 of which were assigned to intra-Hsp40 interactions. Aligning the XLs with the structural elements of Hsp40 (Li et al., 2003; Table S4) places many of the interactions between the J domains (K23, K24, K28, K32, and K37) and the adjacent β-sheet region, which is linked by an unstructured 29 amino acid linker (Figure 2D). The very high level of inter-XL observed for Hsp40 (Figure 2D) indicates its open, flexible structure.

Seven inter-XLs define interactions between Hsp40 and $Hsp70_{E.\ coli}$. One XL is formed between the J domain and the Hsp70 SBD (K32-K524). The SBD of Hsp70 is further aligned via K512-K152 in Hsp40. An Hsp40 residue (K207) crosslinks to two distal residues (K77 and K550) on Hsp70 in the NBD and lid, respectively (Figure 3A). These XLs are not compatible with a single Hsp70-Hsp40, but are consistent with one Hsp70 bridging an Hsp40 dimer, allowing interactions with the two K207 residues in the Hsp40 dimer with both the lid and NBD of Hsp70. This is in accord with previous results that locate Hsp40 at the IEEVD motif of Hsp70 and between the Hsp40 J domains and an acidic groove in the NBD of Hsp70 in the ATP state (Qi et al., 2013; Figure 3A).

Given that Hsp40 stimulates the ATPase activity of Hsp70, and the observation of the J domain close to the nucleotide-binding site in Hsp70 (residues 24–246), we also considered Hsp70 in the undocked ADP form. Four XLs can be accommodated with

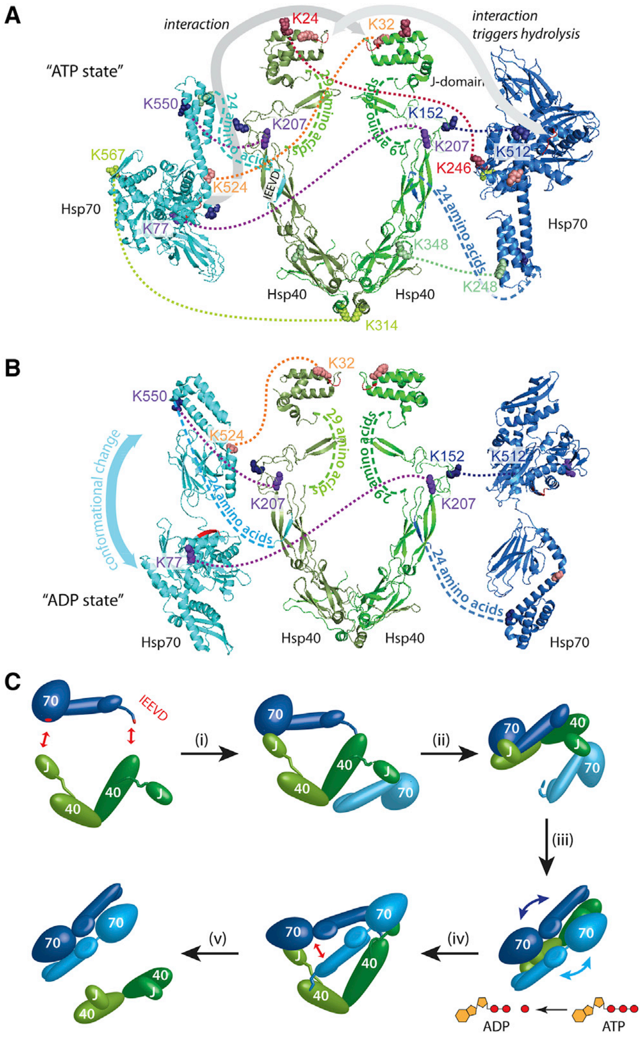

Figure 3. Proposed Dimerization Model of Hsp70 following Interactions with Hsp40

(A) XL reveals seven critical inter-subunit interactions that allow alignment of the Hsp70 dimer in an antiparallel ATP state with Hsp40 (missing C-terminal residues of Hsp70 are indicated). Following stimulation of the ATPase activity of Hsp70 through interactions with Hsp40 J domains, the predominant conformation is likely the undocked ADP state.

(B) The same XLs as in (A) can be rationalized in this structure, with the exception of Hsp70:40 XLs 567-314 and 248-348, which are better accommodated in the ATP state of Hsp70.

(C) Schematic of the J domains binding Hsp70 to bring the C-terminal IEEVD motif of Hsp70 into close proximity to the binding site on Hsp40, placing Hsp70 in a well-defined position across the Hsp40 dimer. The second Hsp40 J domain can bind to a second Hsp70, positioning it in close proximity to the first Hsp70 and triggering the formation of an antiparallel Hsp70 dimer. Hsp40 stimulates ATPase hydrolysis of Hsp70 and induces a conformational change from the docked to the undocked form. Following Hsp70 dimerization, the Hsp40 dimer dissociates from Hsp70.

Hsp70 molecules in the ADP conformation (Figure 3B). The fact that a subset of XLs can be accommodated in docked and undocked states of Hsp70 is consistent with the equilibrium that exists in solution. Moreover, the same inter-Hsp70 XLs defined above confirm the antiparallel Hsp70 dimer in both docked and undocked conformations (Table S2).

As a consequence of these interactions, movement is restricted, with two Hsp70 subunits held in an antiparallel orientation for dimerization. Overall, these data provide molecular details of the interactions involved in bringing Hsp70 subunits together, with the J domains of Hsp40 binding to the NBD of Hsp70, stimulating ATPase activity and inducing conformational changes necessary to prime Hsp70 for substrate binding (Figure 3C).

Antiparallel Dimers of Hsp70 Facilitate Client Transfer to Hsp90

A key question prompted by the antiparallel arrangement of Hsp70 subunits is whether they are an integral part of chaperone complexes involving Hsp90. To address this question, we compared the effect of PTMs on $Hsp70_{Sf9}$ versus $Hsp70_{E.\ coli}$ on interactions with Hop and Hsp90 in the absence of Hsp40. We found that the predominant heterocomplex for $Hsp70_{E.\ coli}$ is $Hsp90_2$Hop, with only a low incorporation of Hsp70 monomer, and a second species at very low intensity containing an additional Hop, $Hsp90_2Hsp70Hop_2$ (Figure 4A). It was not possible to form complexes containing more $Hsp70_{E.\ coli}$ than Hop subunits. For $Hsp70_{Sf9}$, there is clear evidence that two molecules of $Hsp70_{Sf9}$ were incorporated into the complex to form $Hsp90_2$-$Hsp70_2$Hop, indicating that $Hsp70_{Sf9}$ is likely incorporated as a dimer with only one Hop (Figure 4B). The formation of this complex is in accord with our earlier proposal that PTMs in $Hsp70_{Sf9}$ promote dimerization. This holds true even in the absence of Hsp40, in complexes with Hsp90 and Hop.

To determine whether Hsp70 dimerization plays a role in forming the client-loading complex, we investigated interactions with GR. We saw no evidence of direct binding of GR to $Hsp90_2$Hop, and therefore formed the Hsp70GR complex in the presence of Hsp40, as above, prior to incubation with the $Hsp90_2$Hop complex. With catalytic quantities of Hsp40 and equimolar ratios of Hsp90, Hop, Hsp70, and GR, we observed two new complexes, $Hsp90_2$Hsp70HopGR and $Hsp90_2Hsp70_2$HopGR, with the complex containing two Hsp70 molecules being predominant (Figure 4C). Less intense charge-state series were observed for complexes without the full cohort of subunits and assigned to intermediates populated during the assembly process. Our results confirm that pre-binding of GR to Hsp70 is favored over dimerization of Hsp70 in the presence of Hsp40, and is a prerequisite for binding GR to Hsp90. Since binding of monomeric Hsp70 to $Hsp90_2$Hop is also favored, however, the observation that the predominant GR-chaperone complex incorporates two Hsp70s implies that dimerization of Hsp70 plays a major role in client binding to the Hsp90 complex.

To test this hypothesis, we increased the concentration of Hsp70 such that two Hsp70s were present per $Hsp90_2$. Under these conditions, and after forming the Hsp70GR complex in the presence of Hsp40, we found that $Hsp90_2Hsp70_2$HopGR was formed almost exclusively (Figure 4D). No other sub-stoichiometric complexes were formed under these conditions. The observation of this highly stable complex, incorporating two copies of Hsp70 with Hop and $Hsp90_2$ together with a client, suggests that this is an important mechanistic step in priming the

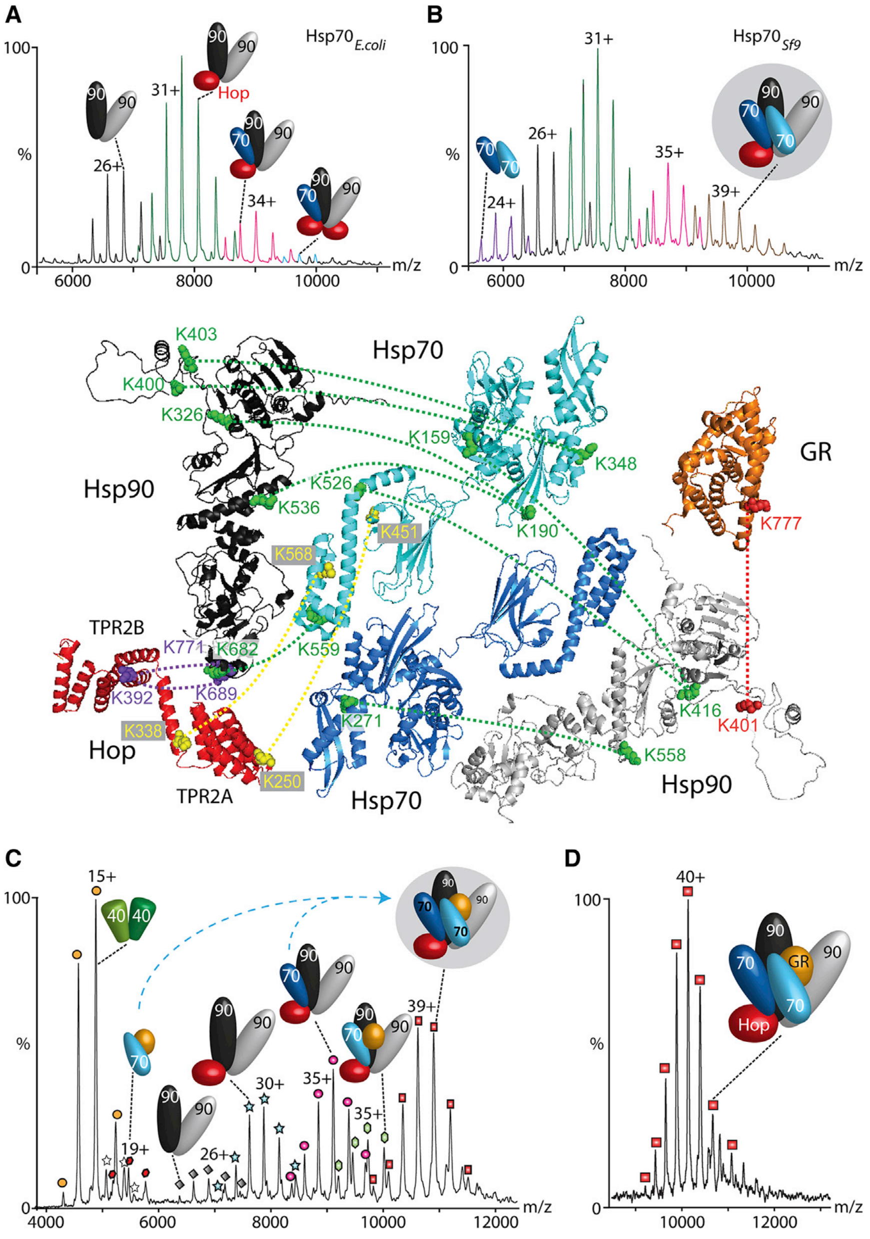

Figure 4. Hsp70 Binds Hsp90, GR, and Hop to Form a Highly Stable Heterohexamer

(A and B) Mass spectra of solutions containing Hsp90 and Hop interacting with either Hsp70$_{E.\ coli}$ (A) or Hsp70$_{Sf9}$ (B), respectively. In the absence of Hsp40, a larger population of the Hsp90$_2$-HopHsp70$_2$ complex is formed for the Sf9 protein.

(C) With catalytic amounts of Hsp40 and one molar equivalent of Hsp70$_{E.\ coli}$, a hexameric complex containing client protein is formed.

(D) When the molar equivalence of Hsp70 is increased, in line with the Hsp90 dimer, a stable client-loading complex is formed. XLs define an Hsp90-Hsp70 interface and additional XLs locate Hop, Hsp70, and Hsp90. Only one XL was observed for GR binding to Hsp90, attributed to its protected position within the client-binding cleft (central panel).

See also Figure S3 and Tables S2 and S3.

later stages of the cycle with a complex predisposed to transfer the client from Hsp70 to Hsp90.

To define the location of subunits within the client-loading complex, we performed XL experiments using BS3-d$_0$ and BS3-d$_4$. To reduce complexity, we separated the crosslinked complexes by SDS-PAGE prior to digestion and LC-MS/MS analysis (Figure S3B). We identified 679 XLs from all protein bands after a database search and validated 366 of these manually, giving an FDR of 46.1%. Rejecting XLs for peptides with fewer than four amino acids yields 102 unique XLs. Disregarding intra-XLs, this leaves 31 inter-XLs. In the absence of high-resolution structures for human Hsp90 and Hop, we generated homology models using Swiss-Model (Table S4). Given that there are no PDB entries for full-length Hop, we used a yeast template comprising the TPR2A- and TPR2B-binding sites, which are known to bind the EEVD motif of Hsp70 and the MEEVD motif of Hsp90, respectively (Scheufler et al., 2000). We then assembled models of the interacting subunits and used these to display the XL restraints (Figure 4, main panel).

Five XLs locate the N-terminal and middle domains of Hsp90 in close proximity to the NBD (K326-K190, K416-K159, K558-K271, and K536-K190) and SBD (K416-K526) of Hsp70. One additional XL between the C-terminal regions of both proteins (K689-K559) defines the vertical alignment. Strikingly, we observed an additional XL between Hsp70 and Hsp90 (K271-K558) that cannot be reconciled with only one Hsp70, and can only be rationalized by antiparallel subunit interactions of a second Hsp70. Two XLs formed between Hop and Hsp90 (K392-K689/771) support the positioning of Hop with upward- and downward-facing TPR2B- and TPR2A-binding motifs, respectively, as suggested previously (Schmid et al., 2012), allowing for simultaneous binding of Hsp90 and Hsp70. Therefore, these 14 XLs confirm earlier findings of an antiparallel Hsp70 dimerization.

In our study, only one XL was found to link Hsp90 and GR, positioning the GR in close proximity to Hsp90. This XL (K401-K777) connects an unstructured linker of Hsp90 to the C-terminal residue of GR. However, given the location of the client (sequestered within a binding-site cleft formed from two copies of both Hsp90 and Hsp70), access to XL reagents is likely to be restricted. According to our results, GR is located in close proximity to the SBD of Hsp70 and the middle domain of Hsp90. This active-site "cleft" for client binding is positioned for interaction

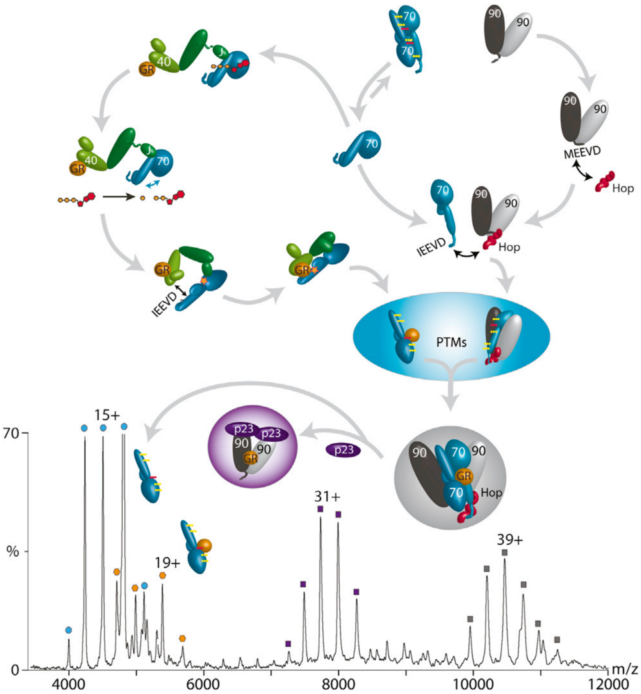

Figure 5. Joining of the Hsp70 and Hsp90 Cycles, and Addition of p23 to the Client-Loading Complex

Hsp40 promotes client transfer to Hsp70, which exists in a monomer-dimer equilibrium. One Hsp40 binds to GR, while the J domain of the second Hsp40 associates with an Hsp70 monomer. This stimulates ATPase activity and induces a conformational change from the docked to the undocked state. Hsp90 binds to Hsp70 via Hop. Hsp40 binding one Hsp70 can then bind the second Hsp70 to form the antiparallel dimer, which is stabilized by PTMs. The joining of the two cycles is mediated by Hsp40 binding only transiently to catalyze the Hsp70 dimerization. Formation of the stable heterohexameric client-binding complex $Hsp90_2Hsp70_2HopGR$ with antiparallel Hsp70 subunits primes the client for transfer to Hsp90. Subsequent interactions with p23, Aha, or immunophilins promote GR maturation and transport to the nucleus. The mass spectrum shows the effect of the addition of p23 to the client-loading complex. An $Hsp90_2GRp23_2$ complex is formed as Hop and Hsp70 are released, thus completing the transfer of GR to Hsp90. See also Tables S2 and S3.

with further cochaperones and for transfer of the client from Hsp70 to Hsp90.

To test whether this cleft is predisposed for handover of GR, we added stoichiometric amounts of the cochaperone p23 (Figure 5). Mass spectra revealed the formation of a new complex assigned as $Hsp90_2p23_2GR$. Interestingly, no Hop or Hsp70 remained in this complex. Therefore, our experiments reconstructed the ATP-driven transfer reaction of GR from Hsp70 to Hsp90, facilitated by the client-loading complex and effected by addition of the cochaperone p23.

DISCUSSION

We have shown that formation of the client-loading complex involves prior binding of Hsp40 and the client to Hsp70, both of which stimulate ATP hydrolysis to place monomeric Hsp70 in the undocked client-binding conformation. Binding of GR to Hsp40, and Hsp70 to the GR-bound Hsp40 dimer, positions GR and Hsp70 in close proximity, forming an Hsp70GR complex devoid of Hsp40. XL reveals that the Hsp40 dimer contacts an Hsp70 in two locations: (1) via its J domain with the acidic groove between the Hsp70 SBD and NBDs, as shown previously (Hennessy et al., 2005), and (2) close to K207 of the second Hsp40 molecule with the IEEVD binding motif of Hsp70. Up to two Hsp70 subunits can bind to an Hsp40 dimer in this way (Figure 5).

The fact that the Hsp70/Hsp40 heterotetramer is not observed and the cellular concentration of Hsp40 is significantly lower than that of Hsp70 implies that this complex has limited stability or is formed only transiently, as proposed previously (Young, 2010). Although a catalytic role of Hsp40 is widely recognized, the Hsp70 dimerization, binding across the two symmetric faces of Hsp40, provides new mechanistic insight. The effect of this binding is to orient the two Hsp70 subunits in such a way that an antiparallel dimer is formed, as evidenced by XL experiments and in agreement with a recent report of DnaJ, the bacterial homolog of Hsp40, and RepE forming a $DnaJ_2RepE_2$ complex (Cuéllar et al., 2013). In addition, antiparallel dimers of DnaK were shown to be important for interaction with Hsp40 (Sarbeng et al., 2015). However, our XL results offer the first molecular details of antiparallel Hsp70 dimerization catalyzed by Hsp40.

Enhanced dimerization of Hsp70 expressed in Sf9 cells instead of *E. coli* was unexpected and attributed to the presence of key PTMs in $Hsp70_{Sf9}$. This is supported by several experiments in which (1) dimer interactions were abolished during dephosphorylation, (2) increasing ionic strength disrupted the dimer interface, (3) multiple acetylation sites aligned along the interface, and (4) a phosphomimic mutant strengthened the dimer interface.

Antiparallel Hsp70 dimers have been reported in X-ray structures of the related yeast protein Hsp110 (Liu and Hendrickson, 2007) and for the *E. coli* protein DnaK (Qi et al., 2013), although rotation around the interface axis was required to satisfy our XL constraints in these structures. It is also noteworthy that we used a DnaK template in which the ATPase activity was abolished and modifications to prevent self-association were introduced. Therefore, the differences between our model and the X-ray structure could arise from the heterogeneity of ATP/ADP-bound states in solution or from the use of the full-length wild-type Hsp70.

We also considered a model involving recognition of the inter-domain linker by the SBD of a second Hsp70 (Figure S4A). Employing a substrate-binding-deficient mutant, we found that dimerization was not impaired, confirming that dimerization does not occur through the flexible linker. The sensitivity to ionic strength (Figure S2) and the fact that dimerization occurs for both undocked and docked states excludes associations in which the inter-domain linker is accessible.

We propose that Hsp70 plays a key role in the Hsp70/Hsp40/GR chaperone system. The transient interactions of Hsp40 with Hsp70 "prime" it for interaction either with a second subunit (Figure 3C) or with GR (Figure 5). A recent study showing that dimerization of Hsp70 is important for interactions with Hsp40 supports this proposal (Sarbeng et al., 2015). The absence of $Hsp70_2GR$ complexes implies that the substrate and interface-binding sites are mutually exclusive. During the formation of

the final client-loading complex defined here, $Hsp90_2Hsp70_2$-HopGR, the Hsp40 dimer orients the Hsp70GR complex onto the second Hsp70 within the $Hsp90_2$Hsp70Hop complex. We propose that it is this Hsp70 dimerization event, mediated by Hsp40, ATP, Hsp90, and Hop, that provides the driving force to join the two cycles (Figure 5).

The observation of an $Hsp90_2Hsp70_2$HopGR complex was at first surprising given early reports that only one Hsp70 is incorporated into the Hsp90/Hop complex (Dittmar et al., 1997, Pratt and Dittmar, 1998). Nonetheless, recent reports of $Hsp90_2$Hsp70HopGR complexes in EM studies and our own data confirm that such a species can also form. The client-loading complex is remarkably stable, and this stability likely arises from the fact that the $Hsp90_2Hsp70_2$ heterotetramer is stabilized not only by Hop but also by GR bridging, as revealed by XL. The stability of this complex is likely necessary to protect client proteins prior to their transfer to Hsp90, as supported by recent EM studies (Kirschke et al., 2014), XL of Hsp90GR, and disassembly of the complex when challenged with p23.

Considering that chaperone concentrations are increased in various diseases, with Hsp70 being twice as high as Hsp90 (Kundrat and Regan, 2010), and an enhanced PTM status being linked to cancer (Dutta et al., 2013), an antiparallel dimer becomes relevant. However, the transient nature of this Hsp70 dimer suggests that it plays a role in larger complexes. The high stability of $Hsp90_2Hsp70_2$HopGR implies that it serves as a holding place, since it is rapidly disassembled by interactions with p23.

The joining of the two cycles through Hsp70 dimerization suggests novel therapeutic approaches. Inhibition of Hsp90 interactions plays a role in cancer therapy (Barrott and Haystead, 2013), and maintaining high levels of Hsp70/Hsp40 is thought to protect against aggregation diseases such as Huntington's (Schaffar et al., 2004). Disrupting crucial interactions between these two cycles could therefore maintain high levels of Hsp70/Hsp40 while enabling inhibition of Hsp90. In this regard, signal propagation between the key acetyl and phospho sites could fine-tune the interface dynamics at the intracellular level. Preventing PTMs of the critical amino acids in the Hsp70 dimer interface could compromise formation of the client-loading complex. As such, inhibiting the respective kinase or acetylase could help regulate interactions within this vital interface, and therefore constitutes a promising avenue for therapeutic intervention.

EXPERIMENTAL PROCEDURES

Proteins

All protein sequences were human except for Hsp40, which was from yeast. Proteins were overexpressed in *E. coli* with an N-terminal His-tag, except when stated otherwise, and purified as previously described (Southworth and Agard, 2011). ^{13}C-labeled $Hsp70_{E.\ coli}$ and ^{15}N-labeled $Hsp70_{E.\ coli}$ were expressed in *E. coli* using M9 media. The GRLBD construct (residues 521–777) contained a phenylalanine F602S mutation to enhance solubility. MBP-GR was used where noted to improve the quality of MS spectra. GR constructs were expressed and purified in the presence of dexamethasone followed by extensive dialysis to remove the ligand. p23 was purified as previously described (McLaughlin et al., 2006). Yeast Hsp40 and the GRLBD construct are referred to as Hsp40 and GR, respectively.

Assembly of Hsp90-Client Complexes

Protein complexes were assembled in binding buffer (30 mM HEPES, 50 mM KCl, 2 mM dithiothreitol, pH 7.5). Individual proteins were analyzed in 100 mM ammonium acetate (AmAc; pH7.5). Binary complexes were formed at 1 μM final concentration unless otherwise stated. Nucleotides were added to a final concentration of 200 μM.

Ternary complexes were formed in binding buffer. Hsp70 and GR were added at equimolar concentrations of 1 μM to a solution containing 0.3 μM Hsp40 and 200 μM ATP-Mg, followed by incubation at room temperature for 45 min. Hsp90/Hsp70/Hop/GR complexes were assembled by incubating Hsp90, Hop, Hsp70, and GR, each at 1 μM with 0.3 μM Hsp40. For MS, the buffer was exchanged to 100 mM AmAc, pH 7.5, using micro Bio-Spin columns (Bio-Rad Laboratories) or Amicon 10 kDa MWCO (Millipore).

MS of Intact Complexes

Spectra were acquired on a QToF II mass spectrometer (Waters) modified for high mass transmission (Sobott et al., 2002). Then, 2.5 μl of the solution was introduced into the mass spectrometer using a gold-coated capillary needle prepared in house (Hernández and Robinson, 2007). Spectra were acquired in the positive ion mode and MS conditions were kept constant while concentration effects were monitored. For instrument parameters, see the Supplemental Experimental Procedures. Spectra were processed with MassLynx V4.1 with minimal smoothing and analyzed using *Mass*ign (Morgner and Robinson, 2012) and Unidec (Marty et al., 2015) software.

Phosphatase Treatment of Hsp70

A 1:1 mixture of 3 μM ^{13}C-labeled $Hsp70_{E.\ coli}$ and $Hsp70_{Sf9}$ with natural abundance isotopes was incubated with and without phosphatase (alkaline calf intestine phosphatase on agarose beads; Sigma-Aldrich) in 100 mM AmAc (pH 7.5), at 4°C for 16 hr. Phosphatase beads were removed by filtration.

Ionic Strength Titration

$Hsp70_{Sf9}$ (2 μM) was incubated with 0.5 μM Hsp40 and 200 μM ATP/Mg^{2+} in binding buffer supplemented with 50, 100, or 300 mM KCl for 1 hr at room temperature. The buffer was exchanged to 100 mM AmAc for MS analysis.

Tryptic Digestion

Proteins were digested with Trypsin in gel as previously described (Shevchenko et al., 1996).

Phosphopeptide Enrichment

Phosphopeptides were enriched using TiO_2. Eluted peptides were dried in a vacuum centrifuge and re-dissolved for LC-MS/MS analysis (Supplemental Experimental Procedures).

Chemical XL

Protein complexes were crosslinked with BS3 in binding buffer. The protein and crosslinker concentrations are listed in Table S2. For experimental details, see the Supplemental Experimental Procedures.

LC-MS/MS and Database Search

Peptides were separated by nano-flow liquid chromatography and directly eluted into an LTQ-Orbitrap XL hybrid mass spectrometer (Thermo Scientific). Potential XLs and phosphorylated/acetylated peptides were identified by searching the raw data against a database (see the Supplemental Experimental Procedures for details).

Generation of Models

We arranged all protein structures/homology models in a possible quaternary structure that satisfied the distance constraints of BS3: 11.4 Å; crosslinked lysine side chains: 6.5 Å (twice); and ≈10 Å for conformational dynamics, resulting in ≈35–40 Å as the maximal distance for two crosslinked residues. Homology models for Hsp90, Hsp70, and Hop were generated using the Swiss-Model Workspace (http://swissmodel.expasy.org/) with yeast and *E. coli* templates (Table S4). For other proteins, we used available PDB files (Table S4).

SUPPLEMENTAL INFORMATION

Supplemental Information includes Supplemental Experimental Procedures, five figures, and four tables and can be found with this article online at http://dx.doi.org/10.1016/j.celrep.2015.03.063.

AUTHOR CONTRIBUTIONS

V.B.-E. purified isotopically labeled proteins and prepared the V438F/T504E mutant. C.S. performed XL experiments. V.B.-E., I.-O.E., and N.A.P. performed MS experiments. N.M., C.S., V.B.-E., I.-O.E., N.A.P., E.M.C., and C.V.R. analyzed data. E.K., S.D., D.A., and S.E.J. contributed proteins and the phosphomimic variant. N.M. and C.V.R. designed research. N.M., C.S., V.B.-E., and C.V.R. wrote the paper with contributions from all authors.

ACKNOWLEDGMENTS

We thank Frances Kondrat (University of Oxford) and Min Yang (University College London) for help with protein preparation and useful discussions and Shabaz Mohammed (University of Oxford) for use of MS instruments. This work was funded by an EU Prospects grant (HEALTH-F4-2008-201648), the MRC, and the Wellcome Trust (WT008150 and WT099141). C.V.R. received funding from the ERC (IMPRESS). V.B.-E. and I.-O.E. received funding from the EPSRC. S.D. received funding from the BP Centenary Murray Edwards College Fund, the Cambridge Commonwealth Trust, and the Department of Chemistry, Cambridge University. C.V.R. is a Royal Society Professor.

Received: January 21, 2015
Revised: March 2, 2015
Accepted: March 24, 2015
Published: April 23, 2015

REFERENCES

Alvira, S., Cuéllar, J., Röhl, A., Yamamoto, S., Itoh, H., Alfonso, C., Rivas, G., Buchner, J., and Valpuesta, J.M. (2014). Structural characterization of the substrate transfer mechanism in Hsp70/Hsp90 folding machinery mediated by Hop. Nat. Commun. *5*, 5484.

Aprile, F.A., Dhulesia, A., Stengel, F., Roodveldt, C., Benesch, J.L., Tortora, P., Robinson, C.V., Salvatella, X., Dobson, C.M., and Cremades, N. (2013). Hsp70 oligomerization is mediated by an interaction between the interdomain linker and the substrate-binding domain. PLoS ONE *8*, e67961.

Barrott, J.J., and Haystead, T.A. (2013). Hsp90, an unlikely ally in the war on cancer. FEBS J. *280*, 1381–1396.

Beltrao, P., Albanèse, V., Kenner, L.R., Swaney, D.L., Burlingame, A., Villén, J., Lim, W.A., Fraser, J.S., Frydman, J., and Krogan, N.J. (2012). Systematic functional prioritization of protein posttranslational modifications. Cell *150*, 413–425.

Benaroudj, N., Batelier, G., Triniolles, F., and Ladjimi, M.M. (1995). Self-association of the molecular chaperone HSC70. Biochemistry *34*, 15282–15290.

Benesch, J.L., Aquilina, J.A., Ruotolo, B.T., Sobott, F., and Robinson, C.V. (2006). Tandem mass spectrometry reveals the quaternary organization of macromolecular assemblies. Chem. Biol. *13*, 597–605.

Bertelsen, E.B., Chang, L., Gestwicki, J.E., and Zuiderweg, E.R. (2009). Solution conformation of wild-type E. coli Hsp70 (DnaK) chaperone complexed with ADP and substrate. Proc. Natl. Acad. Sci. USA *106*, 8471–8476.

Bledsoe, R.K., Montana, V.G., Stanley, T.B., Delves, C.J., Apolito, C.J., McKee, D.D., Consler, T.G., Parks, D.J., Stewart, E.L., Willson, T.M., et al. (2002). Crystal structure of the glucocorticoid receptor ligand binding domain reveals a novel mode of receptor dimerization and coactivator recognition. Cell *110*, 93–105.

Brychzy, A., Rein, T., Winklhofer, K.F., Hartl, F.U., Young, J.C., and Obermann, W.M. (2003). Cofactor Tpr2 combines two TPR domains and a J domain to regulate the Hsp70/Hsp90 chaperone system. EMBO J. *22*, 3613–3623.

Chang, Y.W., Sun, Y.J., Wang, C., and Hsiao, C.D. (2008). Crystal structures of the 70-kDa heat shock proteins in domain disjoining conformation. J. Biol. Chem. *283*, 15502–15511.

Chen, S., and Smith, D.F. (1998). Hop as an adaptor in the heat shock protein 70 (Hsp70) and hsp90 chaperone machinery. J. Biol. Chem. *273*, 35194–35200.

Cuéllar, J., Perales-Calvo, J., Muga, A., Valpuesta, J.M., and Moro, F. (2013). Structural insights into the chaperone activity of the 40-kDa heat shock protein DnaJ: binding and remodeling of a native substrate. J. Biol. Chem. *288*, 15065–15074.

Cyr, D.M., and Douglas, M.G. (1994). Differential regulation of Hsp70 subfamilies by the eukaryotic DnaJ homologue YDJ1. J. Biol. Chem. *269*, 9798–9804.

Dittmar, K.D., Demady, D.R., Stancato, L.F., Krishna, P., and Pratt, W.B. (1997). Folding of the glucocorticoid receptor by the heat shock protein (hsp) 90-based chaperone machinery. The role of p23 is to stabilize receptor.hsp90 heterocomplexes formed by hsp90.p60.hsp70. J. Biol. Chem. *272*, 21213–21220.

Dutta, A., Girotra, M., Merchant, N., Nair, P., and Dutta, S.K. (2013). Evidence of multimeric forms of HSP70 with phosphorylation on serine and tyrosine residues—implications for roles of HSP70 in detection of GI cancers. Asian Pac. J. Cancer Prev. *14*, 5741–5745.

Ebong, I.O., Morgner, N., Zhou, M., Saraiva, M.A., Daturpalli, S., Jackson, S.E., and Robinson, C.V. (2011). Heterogeneity and dynamics in the assembly of the heat shock protein 90 chaperone complexes. Proc. Natl. Acad. Sci. USA *108*, 17939–17944.

Heck, A.J. (2008). Native mass spectrometry: a bridge between interactomics and structural biology. Nat. Methods *5*, 927–933.

Hennessy, F., Nicoll, W.S., Zimmermann, R., Cheetham, M.E., and Blatch, G.L. (2005). Not all J domains are created equal: implications for the specificity of Hsp40-Hsp70 interactions. Protein Sci. *14*, 1697–1709.

Hernández, H., and Robinson, C.V. (2007). Determining the stoichiometry and interactions of macromolecular assemblies from mass spectrometry. Nat. Protoc. *2*, 715–726.

Hernández, M.P., Chadli, A., and Toft, D.O. (2002). HSP40 binding is the first step in the HSP90 chaperoning pathway for the progesterone receptor. J. Biol. Chem. *277*, 11873–11881.

Hilton, G.R., and Benesch, J.L. (2012). Two decades of studying non-covalent biomolecular assemblies by means of electrospray ionization mass spectrometry. J. R. Soc. Interface *9*, 801–816.

Jackson, S.E. (2013). Hsp90: structure and function. Top. Curr. Chem. *328*, 155–240.

Jiang, J., Prasad, K., Lafer, E.M., and Sousa, R. (2005). Structural basis of interdomain communication in the Hsc70 chaperone. Mol. Cell *20*, 513–524.

Jiang, J., Maes, E.G., Taylor, A.B., Wang, L., Hinck, A.P., Lafer, E.M., and Sousa, R. (2007). Structural basis of J cochaperone binding and regulation of Hsp70. Mol. Cell *28*, 422–433.

Kampinga, H.H., and Craig, E.A. (2010). The HSP70 chaperone machinery: J proteins as drivers of functional specificity. Nat. Rev. Mol. Cell Biol. *11*, 579–592.

King, C., Eisenberg, E., and Greene, L.E. (1999). Interaction between Hsc70 and DnaJ homologues: relationship between Hsc70 polymerization and ATPase activity. Biochemistry *38*, 12452–12459.

Kirschke, E., Goswami, D., Southworth, D., Griffin, P.R., and Agard, D.A. (2014). Glucocorticoid receptor function regulated by coordinated action of the Hsp90 and Hsp70 chaperone cycles. Cell *157*, 1685–1697.

Kityk, R., Kopp, J., Sinning, I., and Mayer, M.P. (2012). Structure and dynamics of the ATP-bound open conformation of Hsp70 chaperones. Mol. Cell *48*, 863–874.

Kundrat, L., and Regan, L. (2010). Balance between folding and degradation for Hsp90-dependent client proteins: a key role for CHIP. Biochemistry *49*, 7428–7438.

Laufen, T., Mayer, M.P., Beisel, C., Klostermeier, D., Mogk, A., Reinstein, J., and Bukau, B. (1999). Mechanism of regulation of hsp70 chaperones by DnaJ cochaperones. Proc. Natl. Acad. Sci. USA *96*, 5452–5457.

Li, J., Qian, X., and Sha, B. (2003). The crystal structure of the yeast Hsp40 Ydj1 complexed with its peptide substrate. Structure *11*, 1475–1483.

Li, J., Wu, Y., Qian, X., and Sha, B. (2006). Crystal structure of yeast Sis1 peptide-binding fragment and Hsp70 Ssa1 C-terminal complex. Biochem. J. *398*, 353–360.

Li, J., Richter, K., and Buchner, J. (2011). Mixed Hsp90-cochaperone complexes are important for the progression of the reaction cycle. Nat. Struct. Mol. Biol. *18*, 61–66.

Liu, Q., and Hendrickson, W.A. (2007). Insights into Hsp70 chaperone activity from a crystal structure of the yeast Hsp110 Sse1. Cell *131*, 106–120.

Mapa, K., Sikor, M., Kudryavtsev, V., Waegemann, K., Kalinin, S., Seidel, C.A., Neupert, W., Lamb, D.C., and Mokranjac, D. (2010). The conformational dynamics of the mitochondrial Hsp70 chaperone. Mol. Cell *38*, 89–100.

Marty, M.T., Baldwin, A.J., Marklund, E.G., Hochberg, G.K., Benesch, J.L., and Robinson, C.V. (2015). Bayesian Deconvolution of Mass and Ion Mobility Spectra: From Binary Interactions to Polydisperse Ensembles. Anal. Chem., Published online March 23, 2015. http://dx.doi.org/10.1021/acs.analchem.5b00140.

Mayer, M.P., and Bukau, B. (2005). Hsp70 chaperones: cellular functions and molecular mechanism. Cell. Mol. Life Sci. *62*, 670–684.

Mayer, M.P., Schröder, H., Rüdiger, S., Paal, K., Laufen, T., and Bukau, B. (2000). Multistep mechanism of substrate binding determines chaperone activity of Hsp70. Nat. Struct. Biol. *7*, 586–593.

McLaughlin, S.H., Sobott, F., Yao, Z.P., Zhang, W., Nielsen, P.R., Grossmann, J.G., Laue, E.D., Robinson, C.V., and Jackson, S.E. (2006). The co-chaperone p23 arrests the Hsp90 ATPase cycle to trap client proteins. J. Mol. Biol. *356*, 746–758.

Morgner, N., and Robinson, C.V. (2012). Massign: an assignment strategy for maximizing information from the mass spectra of heterogeneous protein assemblies. Anal. Chem. *84*, 2939–2948.

Picard, D., Khursheed, B., Garabedian, M.J., Fortin, M.G., Lindquist, S., and Yamamoto, K.R. (1990). Reduced levels of hsp90 compromise steroid receptor action in vivo. Nature *348*, 166–168.

Pratt, W.B., and Dittmar, K.D. (1998). Studies with purified chaperones advance the understanding of the mechanism of glucocorticoid receptor-hsp90 heterocomplex assembly. Trends Endocrinol. Metab. *9*, 244–252.

Pratt, W.B., and Toft, D.O. (1997). Steroid receptor interactions with heat shock protein and immunophilin chaperones. Endocr. Rev. *18*, 306–360.

Prodromou, C. (2012). The 'active life' of Hsp90 complexes. Biochim. Biophys. Acta *1823*, 614–623.

Qi, R., Sarbeng, E.B., Liu, Q., Le, K.Q., Xu, X., Xu, H., Yang, J., Wong, J.L., Vorvis, C., Hendrickson, W.A., et al. (2013). Allosteric opening of the polypeptide-binding site when an Hsp70 binds ATP. Nat. Struct. Mol. Biol. *20*, 900–907.

Richter, K., Muschler, P., Hainzl, O., Reinstein, J., and Buchner, J. (2003). Sti1 is a non-competitive inhibitor of the Hsp90 ATPase. Binding prevents the N-terminal dimerization reaction during the atpase cycle. J. Biol. Chem. *278*, 10328–10333.

Rohrer, K.M., Haug, M., Schwörer, D., Kalbacher, H., and Holzer, U. (2014). Mutations in the substrate binding site of human heat-shock protein 70 indicate specific interaction with HLA-DR outside the peptide binding groove. Immunology *142*, 237–247.

Sanchez, E.R. (2012). Chaperoning steroidal physiology: lessons from mouse genetic models of Hsp90 and its cochaperones. Biochim. Biophys. Acta *1823*, 722–729.

Sarbeng, E.B., Liu, Q., Tian, X., Yang, J., Li, H., Wong, J.L., Zhou, L., and Liu, Q. (2015). A functional DnaK dimer is essential for the efficient interaction with heat shock protein 40 kDa (Hsp40). J. Biol. Chem. *290*, 8849–8862.

Schaffar, G., Breuer, P., Boteva, R., Behrends, C., Tzvetkov, N., Strippel, N., Sakahira, H., Siegers, K., Hayer-Hartl, M., and Hartl, F.U. (2004). Cellular toxicity of polyglutamine expansion proteins: mechanism of transcription factor deactivation. Mol. Cell *15*, 95–105.

Scheufler, C., Brinker, A., Bourenkov, G., Pegoraro, S., Moroder, L., Bartunik, H., Hartl, F.U., and Moarefi, I. (2000). Structure of TPR domain-peptide complexes: critical elements in the assembly of the Hsp70-Hsp90 multichaperone machine. Cell *101*, 199–210.

Schmid, A.B., Lagleder, S., Gräwert, M.A., Röhl, A., Hagn, F., Wandinger, S.K., Cox, M.B., Demmer, O., Richter, K., Groll, M., et al. (2012). The architecture of functional modules in the Hsp90 co-chaperone Sti1/Hop. EMBO J. *31*, 1506–1517.

Schmidt, C., and Robinson, C.V. (2014). A comparative cross-linking strategy to probe conformational changes in protein complexes. Nat. Protoc. *9*, 2224–2236.

Schmidt, C., Zhou, M., Marriott, H., Morgner, N., Politis, A., and Robinson, C.V. (2013). Comparative cross-linking and mass spectrometry of an intact F-type ATPase suggest a role for phosphorylation. Nat. Commun. *4*, 1985.

Shevchenko, A., Wilm, M., Vorm, O., and Mann, M. (1996). Mass spectrometric sequencing of proteins silver-stained polyacrylamide gels. Anal. Chem. *68*, 850–858.

Smith, D.F. (1993). Dynamics of heat shock protein 90-progesterone receptor binding and the disactivation loop model for steroid receptor complexes. Mol. Endocrinol. *7*, 1418–1429.

Sobott, F., Hernández, H., McCammon, M.G., Tito, M.A., and Robinson, C.V. (2002). A tandem mass spectrometer for improved transmission and analysis of large macromolecular assemblies. Anal. Chem. *74*, 1402–1407.

Southworth, D.R., and Agard, D.A. (2011). Client-loading conformation of the Hsp90 molecular chaperone revealed in the cryo-EM structure of the human Hsp90:Hop complex. Mol. Cell *42*, 771–781.

Stengel, F., Baldwin, A.J., Painter, A.J., Jaya, N., Basha, E., Kay, L.E., Vierling, E., Robinson, C.V., and Benesch, J.L. (2010). Quaternary dynamics and plasticity underlie small heat shock protein chaperone function. Proc. Natl. Acad. Sci. USA *107*, 2007–2012.

Swain, J.F., Dinler, G., Sivendran, R., Montgomery, D.L., Stotz, M., and Gierasch, L.M. (2007). Hsp70 chaperone ligands control domain association via an allosteric mechanism mediated by the interdomain linker. Mol. Cell *26*, 27–39.

Taipale, M., Jarosz, D.F., and Lindquist, S. (2010). HSP90 at the hub of protein homeostasis: emerging mechanistic insights. Nat. Rev. Mol. Cell Biol. *11*, 515–528.

Thompson, A.D., Bernard, S.M., Skiniotis, G., and Gestwicki, J.E. (2012). Visualization and functional analysis of the oligomeric states of Escherichia coli heat shock protein 70 (Hsp70/DnaK). Cell Stress Chaperones *17*, 313–327.

Tsutsumi, S., Mollapour, M., Prodromou, C., Lee, C.T., Panaretou, B., Yoshida, S., Mayer, M.P., and Neckers, L.M. (2012). Charged linker sequence modulates eukaryotic heat shock protein 90 (Hsp90) chaperone activity. Proc. Natl. Acad. Sci. USA *109*, 2937–2942.

van Noort, V., Seebacher, J., Bader, S., Mohammed, S., Vonkova, I., Betts, M.J., Kühner, S., Kumar, R., Maier, T., O'Flaherty, M., et al. (2012). Cross-talk between phosphorylation and lysine acetylation in a genome-reduced bacterium. Mol. Syst. Biol. *8*, 571.

Wu, C.C., Naveen, V., Chien, C.H., Chang, Y.W., and Hsiao, C.D. (2012). Crystal structure of DnaK protein complexed with nucleotide exchange factor GrpE in DnaK chaperone system: insight into intermolecular communication. J. Biol. Chem. *287*, 21461–21470.

Wyttenbach, T., and Bowers, M.T. (2007). Intermolecular interactions in biomolecular systems examined by mass spectrometry. Annu. Rev. Phys. Chem. *58*, 511–533.

Young, J.C. (2010). Mechanisms of the Hsp70 chaperone system. Biochem. Cell Biol. *88*, 291–300.

Young, J.C., Obermann, W.M., and Hartl, F.U. (1998). Specific binding of tetratricopeptide repeat proteins to the C-terminal 12-kDa domain of hsp90. J. Biol. Chem. *273*, 18007–18010.

Young, J.C., Moarefi, I., and Hartl, F.U. (2001). Hsp90: a specialized but essential protein-folding tool. J. Cell Biol. *154*, 267–273.

Zhuravleva, A., Clerico, E.M., and Gierasch, L.M. (2012). An interdomain energetic tug-of-war creates the allosterically active state in Hsp70 molecular chaperones. Cell *151*, 1296–1307.

Cell Reports

Supplemental Information

Hsp70 Forms Antiparallel Dimers Stabilized by Post-translational Modifications to Position Clients for Transfer to Hsp90

Nina Morgner, Carla Schmidt, Victoria Beilsten-Edmands, Ima-obong Ebong, Nisha A. Patel, Eugenia M. Clerico, Elaine Kirschke, Soumya Daturpalli, Sophie E. Jackson, David Agard, and Carol V. Robinson

Supplemental Figures

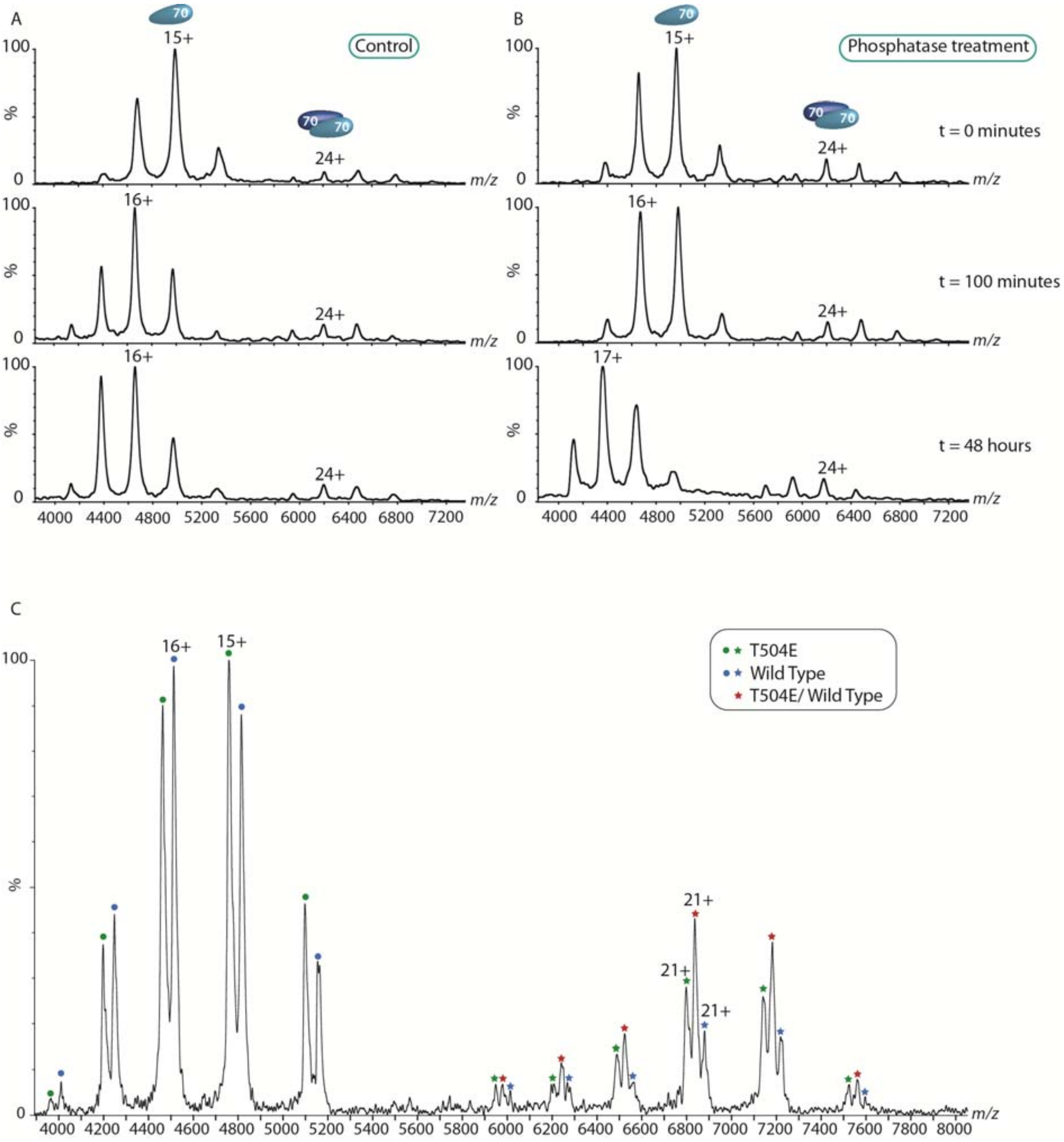

Figure S1 (related to Figure 1): Phosphatase treatment of Hsp70$_{E. coli}$ and dimerization of the phosphomimic variant of Hsp70$_{E. coli}$ T504E. Phosphatase treatment does not reduce dimerization in Hsp70$_{E. coli}$ over a 48 hour time period. 6 µM ^{13}C-labeled wild-type Hsp70$_{E. coli}$ incubated without phosphatase **(A)** or in the presence of phosphatase **(B)**. **(C)** The T504E variant was incubated in a 1:1 ratio with the ^{15}N-labeled wild type protein in the presence of ATP to compare directly the intensities of the Hsp70-dimer. The phosphomimic variant T504E shows a greater proportion of dimer compared to the wild type protein.

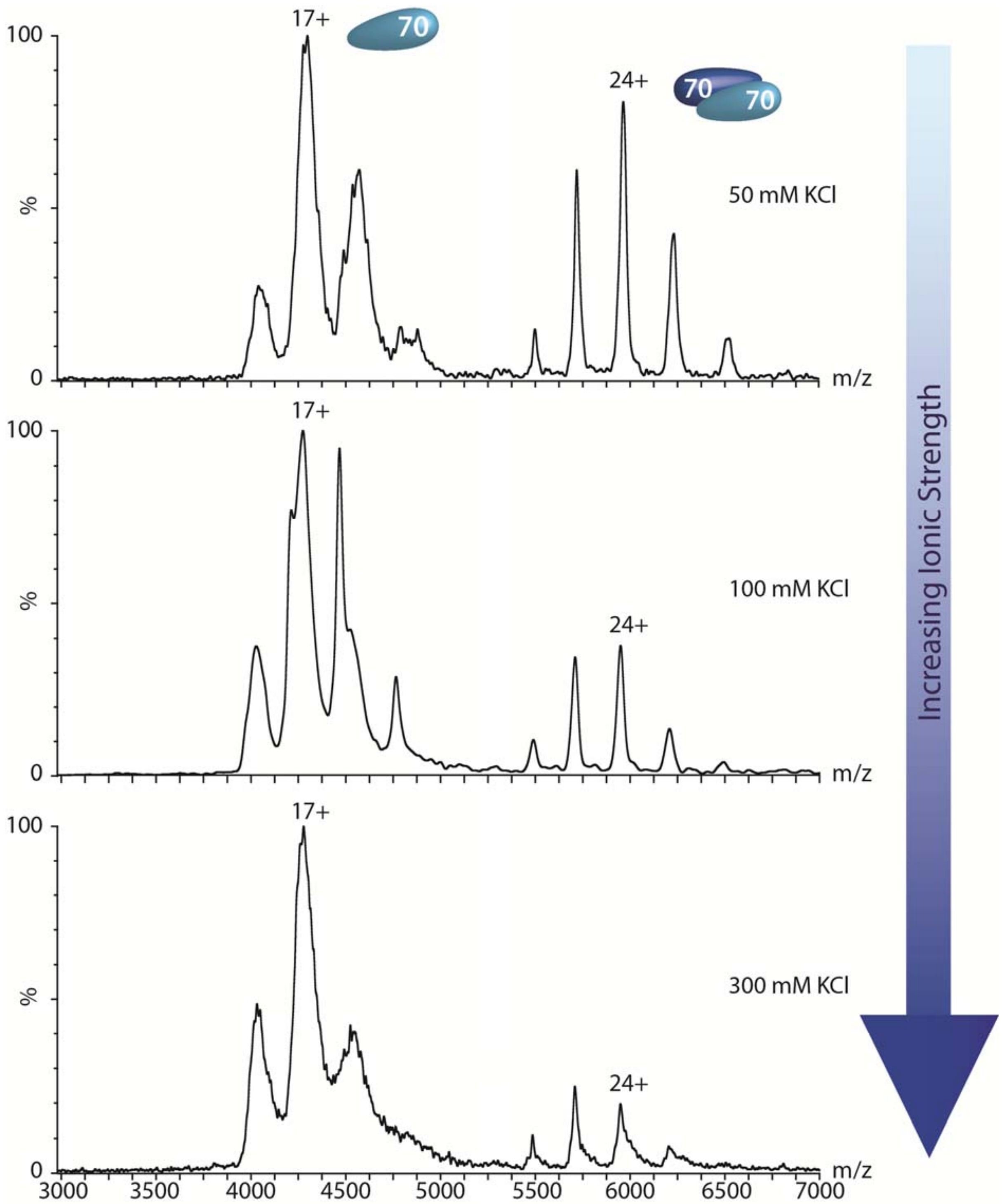

Figure S2 (related to Figure 1): The Hsp70-dimer is sensitive to increasing ionic strength. 2 µM $Hsp70_{Sf9}$ with 0.5 µM Hsp40 and ATP was analysed by mass spectrometry from three different solution conditions containing different ionic strength of the binding buffer (from 50 mM to 300 mM KCl). The mass spectra were recorded at high pressure to promote dimer formation. The intensity of the Hsp70-dimer peaks decreases at higher ionic strength.

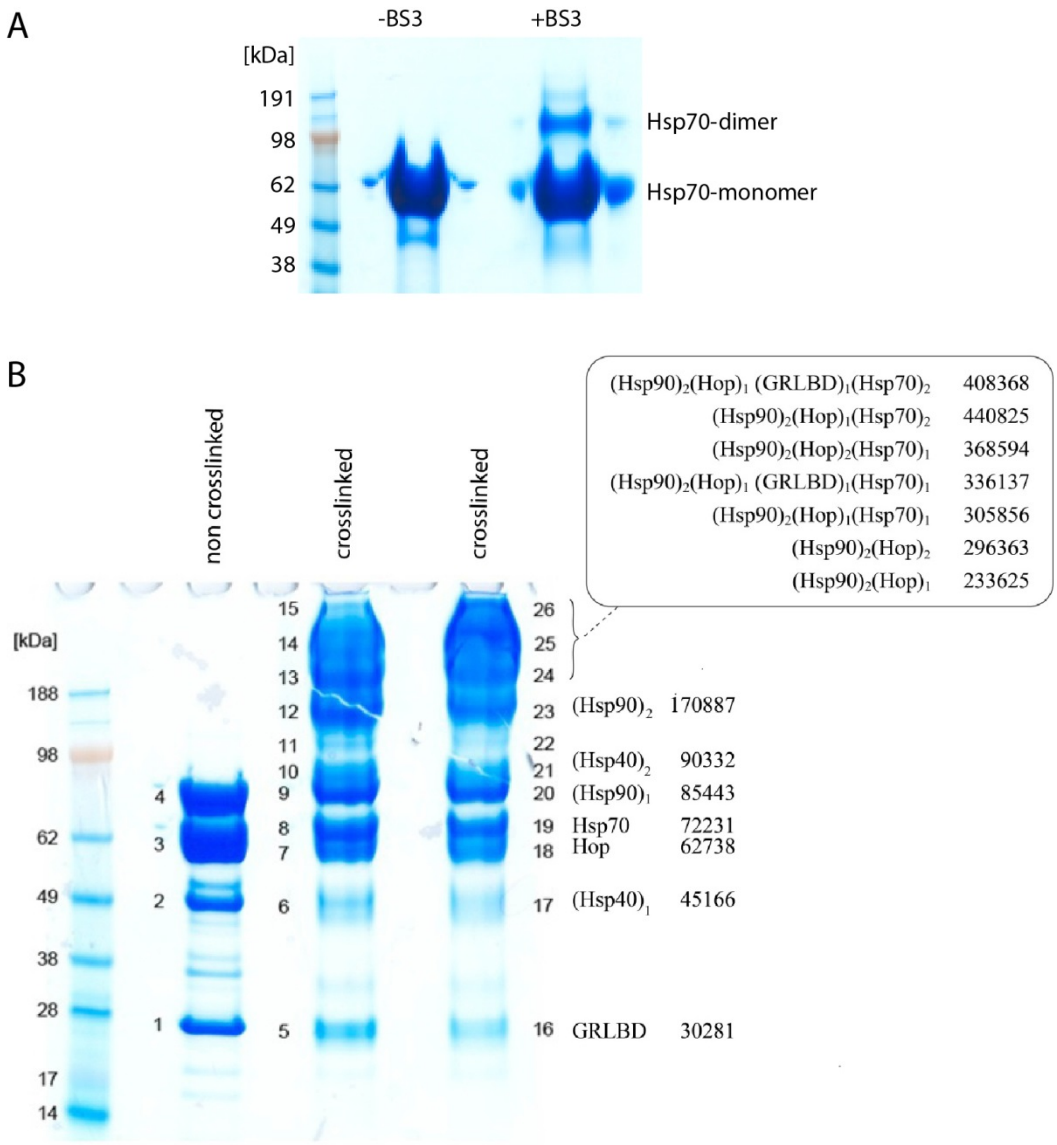

Figure S3 (related to Figures 1 and 4): SDS-PAGE of dimeric Hsp70$_{Sf9}$ and the client-loading complex. (A) Hsp70$_{Sf9}$ was incubated with or without BS3-d0/d4 and analysed by SDS-PAGE. The gel shows monomeric Hsp70$_{Sf9}$ in the control sample (-BS3) and monomeric and dimeric Hsp70$_{Sf9}$ in the cross-linked sample (+BS3). **(B)** Two different concentrations of BS3 cross-linker were used. Additional protein bands are visible after crosslinking. Bands 1-26 were excised for LC-MS/MS and cross-linked peptides were identified as described. Protein masses in Da are given for single protein subunits* as well as possible (sub-) complexes.

*Hsp70$_{E. coli}$ was used for this experiment.

A

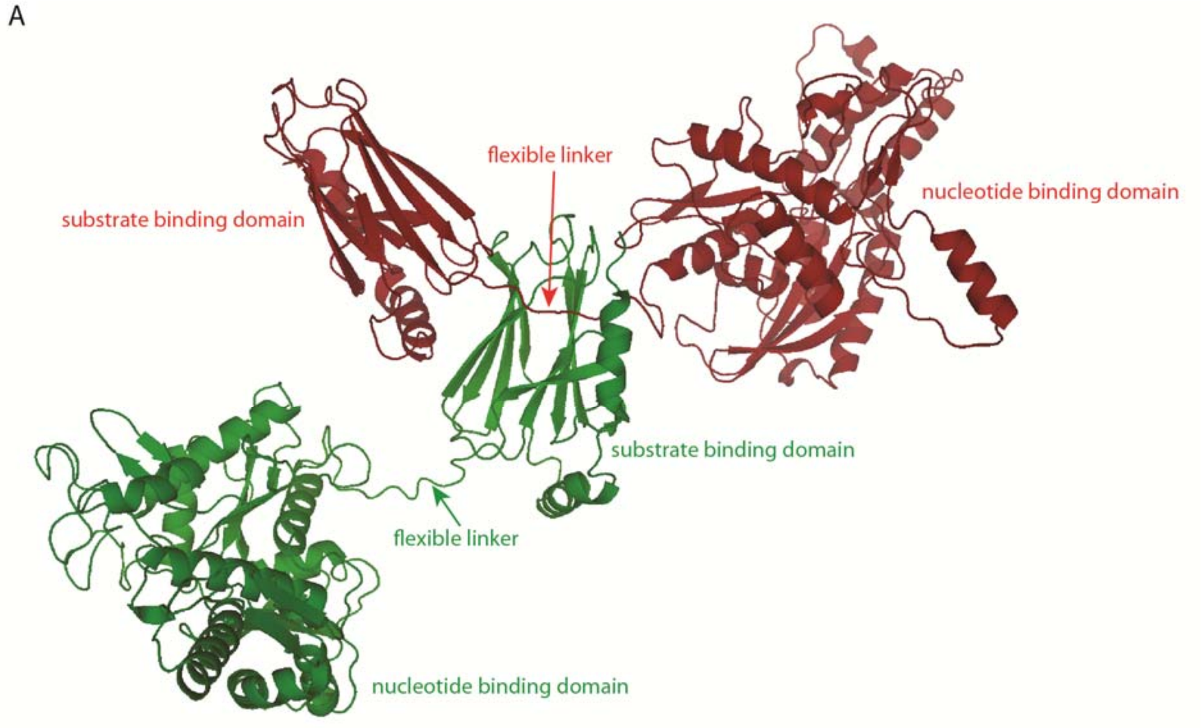

B

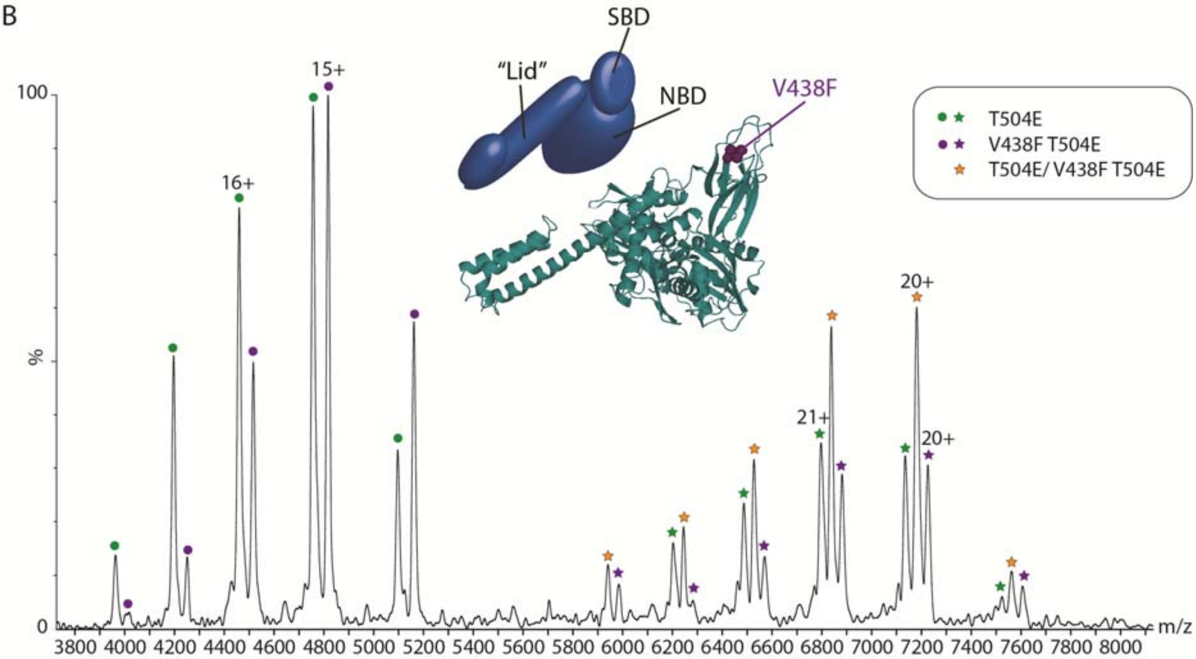

Figure S4 (related to Figure 1): Dimerization of Hsp70 in a substrate-like fashion and dimerization of the substrate binding-deficient variant of Hsp70$_{E.\ coli}$ V438F T504E. (A) One Hsp70 (green) is binding the linker between substrate and nucleotide binding domain of a second Hsp70 (red). The second Hsp70 (red) thus represents a substrate. PDB ID 4ANI (Wu et al., 2012). **(B)** The T504E variant was incubated with the ^{15}N-labeled V438F/T504E variant in the presence of ATP to compare directly the intensities of the Hsp70-dimer. The substrate binding-deficient variant dimerizes and this dimerization is at a similar level compared with the phosphomimic variant.

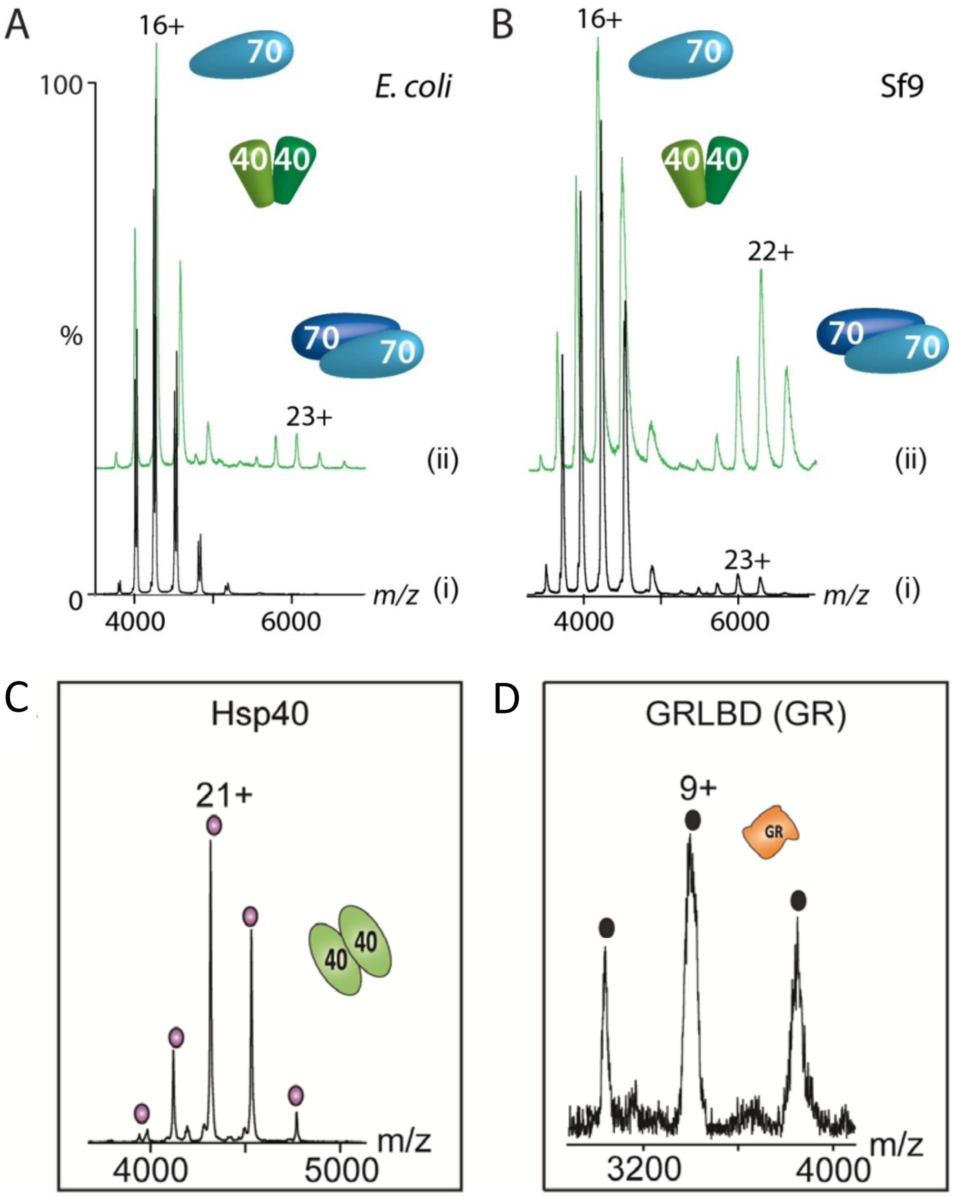

Figure S5 (related to Figure 2): Dimerization of Hsp70$_{E.\ coli}$ and Hsp70$_{Sf9}$ in the presence of Hsp40 and oligomeric states of Hsp40 and GR. (A) and **(B):** (i) Without Hsp40, (ii) in the presence of catalytic amounts of Hsp40. **(A)** Hsp70$_{E.coli}$ dimer is observed*. **(B)** Hsp70$_{Sf9}$ dimer intensity is increased. **(C)** and **(D):** The oligomeric states of Hsp40 and GRLBD were studied using mass spectrometry. Hsp40 was found to be predominantly dimeric under these conditions **(C)** while GRLBD is monomeric **(D)**.

*Hsp70$_{E.\ coli}$ contains His-tag

Supplemental Tables

Table S1 (related to Figure 1): Acetylation sites identified in ($Hsp70_{Sf9}$). Seven acetylation sites were identified by LC-MS/MS. Two of these sites were reported previously (Choudhary et al., 2009, Yang et al., 2013). The site, the peptide sequence, the number of spectra observed and the highest Mascot score for each acetylation site are given.

Acetylation site	Peptide sequence	# Spectra	Highest Mascot score	Identified previously
108	VQVSYK*GETK	8	44.57	Yes[1]
159	QATK*DAGVIAGLNVLR	7	89.72	Yes[2]
451	AMTK*DNNLLGR	40	58.48	No
507	ITITNDK*GR	4	40.27	No
512	LSK*EEIER	46	39.74	No
559	SAVEDEGLK*GK	3	60.40	No
561	GK*ISEADKK	4	36.74	No

Table S2 (related to Figures 1, 2, 3 and 4): Chemical cross-linking of Hsp70/Hsp90 complexes and subcomplexes. For every experiment in this study the protein concentration and volume, concentration and volume of the cross-linker (BS3), the number of cross-linked spectra, the number of unique cross-links and the FDR are listed. The peptide sequences of cross-linked di-peptides, the respective protein names and amino acid residues as well as the number of observed spectra and the maximum MassMatrix (MM) peptide score are given.

Table S3 (related to Figures 1, 2, 4 and 5). Masses of protein subunits and protein complexes observed in this study. Theoretical (expected) and experimentally measured masses of proteins and protein complexes are given in Da.

**Protein subunits**	**Expected mass (Da)**	**Measured mass (Da)**
GRLBD	30281	30291 ± 15
$(Hsp40)_2$	90332	90622 ± 13
$(Hsp90)_1$	85443	85521 ± 86
$(Hsp90)_2$	170887	170960 ± 70
$Hsp70_{E. coli}$*	72231	72656 ± 15
$Hsp70_{Sf9}$	70009	70922 ± 35
$Hsp70_{E. coli}$ ^{13}C-labeled**	73774	73843 ± 6
$Hsp70_{E. coli}$ ^{15}N-labeled	71500	72170 ± 19
$Hsp70_{T504E}$	70667	71256 ± 17
$Hsp70_{T504E\ V438F}$ ^{15}N-labeled	71576	72250 ± 4
**Binary complexes**	**Expected mass (Da)**	**Measured mass (Da)**
$(Hsp70_{E. coli})_2$*	144462	145435 ± 33
$(Hsp70_{Sf9})_2$	140018	141822 ± 20
$(Hsp70_{E. coli}$ ^{13}C-labeled$)_2$	147548	148844 ± 35
$(Hsp70_{E. coli}$ ^{15}N-labeled$)_2$	142999	144447 ± 35
$(Hsp70_{T504E})_2$	141334	142724 ± 24
$(Hsp70_{T504E\ V438F}$ ^{15}N-labeled$)_2$	143151	144517 ± 14
$(Hsp70_{Sf9})(Hsp70_{E. coli}$ ^{13}C-labeled)	143783	145010 ± 23
$(Hsp70_{T504E})(Hsp70_{T504E\ V438F}$ ^{15}N-labeled)	142243	143611 ± 13
$(Hsp70_{T504E})(Hsp70_{E. coli}$ ^{15}N-labeled)	142167	143539 ± 43
$(Hsp90)_2(Hop)_1$	233625	233825 ± 55
**Ternary complexes**	**Expected mass (Da)**	**Measured mass (Da)**
$(Hsp90)_2(Hop)_1(Hsp70_{E. coli})_1$*	305856	306587 ± 70
$(Hsp90)_2(Hop)_1(Hsp70_{Sf9})_1$	304253	305253 ± 40
$(Hsp90)_2(Hop)_1(Hsp70_{Sf9})_2$	374891	375422 ± 33
$(Hsp90)_2(Hop)_2(Hsp70_{E. coli})_1$*	368594	370065 ± 61
**GR complexes**	**Expected mass (Da)**	**Measured mass (Da)**
$(Hsp70_{E. coli})_1(GRLBD)_1$*	102512	103004 ± 20
$(Hsp40)_2(GRLBD)_1$	120613	121084 ± 19
$(Hsp90)_2(Hop)_1(GRLBD)_1(Hsp70_{E. coli})_1$*	336137	337294 ± 62
$(Hsp90)_2(Hop)_1(GRLBD)_1(Hsp70_{E. coli})_2$*	408368	410063 ± 89
$(Hsp90)_2(Hop)_1(GRLBD)_1(Hsp70_{Sf9})_2$	405172	405963 ± 50

*$Hsp70_{E. coli}$ contains His-tag

**$Hsp70_{E. coli}$ nucleotide free-form

Table S4 (related to 'Methods'): Template PDB IDs used to generate homology models and PDB IDs of used high-resolution structures.

Protein	Template PDB ID (organism)	Sequence identity with template
Hsp90	2CG9 (*S. cerevisiae*)	58 %
Hsp70 (ATP state)	4B9Q (*E. coli*)	48 %
Hop (TPR2A and B)	3UQ3 (*S. cerevisiae*)	45 %
**Protein**	**PDB ID (organism)**	
Hsp70 (ADP state)	2KHO (*E. coli*)	
Hsp40	1NLT (*S. cerevisiae*)	
Hsp40 dimerization domain	1XAO (*S. cerevisiae*)	
J-domain of Hsp 40	2O37 (*S. cerevisiae*)	
GRLBD	3E7C (*H. sapiens*)	

Supplemental Methods

MS instrument parameters for intact protein complexes

Individual proteins: capillary voltage 1.7 kV, cone voltage 60 V, extractor 5 V, collision voltage 40 V, backing pressure 3.8 x 10^{-3} - 6.2 x 10^{-3} mbar.

Hsp70-Hsp40-GR complexes: capillary voltage 1.8 kV, cone voltage 100 V, extractor 5 V, collision voltage 80V, backing pressure 1.2 x 10^{-2} mbar.

Hsp90 complexes with Hop, Hsp70 and GR: capillary voltage 1.7-1.8 kV, cone voltage 100 V, extractor 5 V, collision voltage 100V, backing pressure 9.65 x 10^{-3} mbar.

For MSMS experiments, collision voltages varied up to 200 V.

Phosphopeptide enrichment

Phosphopeptides were enriched using titanium dioxide (TiO_2). Enrichment columns were packed into pipette tips using TiO_2 material (GL Sciences). The columns were washed with 5 % TFA/80 % ACN and reconstituted in 20 % 2,5-dihydroxybenzoic acid (DHB)/5 % TFA/80 % ACN. Peptides were dissolved in 20 % DHB/5 % TFA/80 % ACN and loaded onto the material. After washing with 20 % DHB/5 % TFA/80 % ACN and 5 % TFA/80 % ACN, phosphopeptides were eluted with 0.3 N ammonia solution (pH > 10.5). Eluted peptides were dried in a vacuum centrifuge for LC-MS/MS analysis.

Chemical XL

Protein complexes were cross-linked with BS3 in binding buffer. The protein and cross-linker concentrations are stated in Table S2. XL reactions were incubated for 1 hr at 25°C and 450 rpm in a thermomixer. Proteins were separated by SDS-PAGE and digested with trypsin in-gel as described (Shevchenko et al., 1996) or were precipitated with ethanol and digested in-solution using RapiGest SF Surfactant (Waters) according to manufacturer's protocols. Peptides obtained from in-solution digestion were re-dissolved in 20 % ACN, 4 % FA and further separated by cation exchange chromatography using SCX stage tips (Thermo Scientific) according to the manufacturer's protocol. Peptides were eluted with different concentrations of ammonium acetate (50 mM, 100 mM, 200 mM and 500 mM) and dried in a vacuum centrifuge. The mixture of cross-linked and non-cross-linked peptides was analysed by LC-MS/MS.

LC-MS/MS for identification of cross-linked peptides and phosphosites

Tryptic peptides were separated by nano-flow reversed-phase liquid chromatography (DionexUltiMate 3000 RSLC nano System, Thermo Scientific; mobile phase A, 0.1 % (v/v) formic acid (FA); mobile phase B, 80 % (v/v) ACN/0.1 % (v/v) FA) coupled to an LTQ-Orbitrap XL mass spectrometer (Thermo Scientific). Peptides were loaded onto a trap column (HPLC column Acclaim® PepMap100, C18, 100 µm I.D. particle size 5µm; Thermo scientific) and separated with a flow rate of 300 nL/min on an analytical C18 capillary column (50 cm, HPLC column Acclaim® PepMap100, C18, 75 µm I.D. particle size 3 µm; Thermo Scientific), with a gradient of 5-80 % (v/v) mobile phase B over 74 min. Peptides were directly eluted into the mass spectrometer.

MS conditions were: spray voltage of 1.8 kV; capillary temperature of 180 °C; normalized collision energy of 35% at an activation of q = 0.25 and an activation time of 30 ms. The LTQ-Orbitrap XL was operated in data-dependent mode. Survey full scan MS spectra were acquired in the Orbitrap (*m*/*z* 300−2000) with a resolution of 30,000 at *m*/*z* 400 and an automatic gain control (AGC) target at 10^6. The five most intense ions were selected for CID in the linear ion trap at an AGC target of 30,000. For identification of phosphopeptides, multistage activation was enabled for neutral loss masses of one, two, three and four phosphosites per doubly and triply charged peptide. Detection in the linear ion trap of previously selected ions was excluded for 30 s. Singly charged ions and ions with unrecognized charge state were excluded. Internal calibration of the Orbitrap was performed using the lock mass option (lock mass: *m*/*z* 445.120025 (Olsen et al., 2005).

LC-MS/MS for identification of acetylation sites

Proteins were separated by SDS-PAGE and digested as described (Shevchenko et al., 1996). Peptides were separated by nano-flow reversed-phase liquid chromatography (EASY nLC 1000, Thermo Scientific; mobile phase A, 0.1 % (v/v) formic acid (FA)/5 % (v/v) DMSO; mobile phase B, 100 % (v/v) ACN/0.1 % (v/v) FA/5 % (v/v) DMSO) coupled to a Q Exactive Orbitrap mass spectrometer (Thermo Scientific). Peptides were loaded onto a trap column (5 mm, PepMap RSLC, C18, 300 µm I.D. particle size 3 µm; Thermo Scientific) and separated with a flow rate of 200 nL/min on an analytical C18 capillary column (50 cm, PepMap RSLC, EASY-spray column, C18, 75 µm I.D. particle size 3 µm; Thermo Scientific), with a gradient of 7-30 % (v/v) mobile phase B over 30 min. Peptides were directly eluted into the mass spectrometer.

MS conditions were: spray voltage of 2.1 kV; capillary temperature of 320 °C. The Q Exactive Orbitrap was operated in data-dependent mode. Survey full scan MS spectra were acquired in the orbitrap (*m*/*z* 350−1500) with a resolution of 70,000 an AGC target at 3×10^6. The ten most intense ions were selected for HCD at an AGC target of 50,000.

Database search

Identification of cross-links. Potential cross-links were identified using the MassMatrix Database Search Engine (Xu et al., 2010). Search parameters were: Tryptic peptides with a maximum of two missed cleavage sites. Carbamidomethylation of cysteine and oxidation of methionine as variable modifications. Mass accuracy filter: 10 ppm for precursor ions, 0.8 Da for fragment ions. Minimum pp and pp2 values 5.0, minimum pptag 1.3. Maximum number of cross-links per peptide was 1. All searches were performed twice including deuterated (d4) and non-deuterated (d0) BS3, respectively. Cross-links were validated by (i) the presence of the peak pair in the MS spectra generated by the d4/d0-BS3-mixture, and (ii) by the quality of the MS/MS spectrum. For comparative cross-linking, extracted ion chromatograms (XICs) for the light and heavy cross-links, respectively, were generated. The two states of Hsp70 were compared by the area of the XICs.

Identification of phosphosites. Raw data were searched against NCBI non-redundant database with *Homo sapiens* taxonomy filter (248,775 sequences) using Mascot v2.4.1 search engine (Matrix Science). Mass accuracy filter: 15 ppm for precursor ions, 0.5 Da for MS/MS fragment ions. Tryptic peptides with maximal two missed cleavage sites. Carbamidomethylation of cysteine and oxidation of methionine as well as phosphorylation of serine, threonine and tyrosine as variable modifications.

Identification of acetylation sites. Raw data were searched against SwissProt database with *Homo sapiens* taxonomy filter (20,353 sequences) using the Mascot v2.4.1 search engine. Mass accuracy filter: 7 ppm for precursor ions, 0.005 Da for MS/MS fragment ions. Tryptic peptides with maximal two missed cleavage sites. Carbamidomethylation of cysteine and oxidation of methionine as well as acetylation of lysine and protein N-termini as variable modifications.

References

CHOUDHARY, C., KUMAR, C., GNAD, F., NIELSEN, M. L., REHMAN, M., WALTHER, T. C., OLSEN, J. V. & MANN, M. 2009. Lysine acetylation targets protein complexes and co-regulates major cellular functions. *Science,* 325**,** 834-40.

OLSEN, J. V., DE GODOY, L. M., LI, G., MACEK, B., MORTENSEN, P., PESCH, R., MAKAROV, A., LANGE, O., HORNING, S. & MANN, M. 2005. Parts per million mass accuracy on an Orbitrap mass spectrometer via lock mass injection into a C-trap. *Mol Cell Proteomics,* 4**,** 2010-21.

SHEVCHENKO, A., WILM, M., VORM, O. & MANN, M. 1996. Mass spectrometric sequencing of proteins silver-stained polyacrylamide gels. *Anal Chem,* 68**,** 850-8.

WU, C. C., NAVEEN, V., CHIEN, C. H., CHANG, Y. W. & HSIAO, C. D. 2012. Crystal structure of DnaK protein complexed with nucleotide exchange factor GrpE in DnaK chaperone system: insight into intermolecular communication. *J Biol Chem,* 287**,** 21461-70.

XU, H., HSU, P. H., ZHANG, L., TSAI, M. D. & FREITAS, M. A. 2010. Database search algorithm for identification of intact cross-links in proteins and peptides using tandem mass spectrometry. *J Proteome Res,* 9**,** 3384-93.

YANG, Y., FISKUS, W., YONG, B., ATADJA, P., TAKAHASHI, Y., PANDITA, T. K., WANG, H. G. & BHALLA, K. N. 2013. Acetylated hsp70 and KAP1-mediated Vps34 SUMOylation is required for autophagosome creation in autophagy. *Proc Natl Acad Sci U S A,* 110**,** 6841-6.
